# Keratins coordinate tissue spreading by balancing spreading forces with tissue material properties

**DOI:** 10.1038/s41467-026-72366-z

**Published:** 2026-05-16

**Authors:** Suyash Naik, Yann-Edwin Keta, Kornelija Pranjic-Ferscha, Édouard Hannezo, Silke Henkes, Carl-Philipp Heisenberg

**Affiliations:** 1 Institute of Science and Technology Austria (ISTA), Klosterneuburg, Austria; 2 Laboratoire de Physique et Mécanique des Milieux Hétérogènes (PMMH), UMR 7636 CNRS, ESPCI Paris - PSL, Sorbonne Université, Université Paris Cité, Paris, France; 3 Lorentz Institute for Theoretical Physics, LION, Leiden University, Leiden, The Netherlands

**Keywords:** Gastrulation, Computational biophysics, Cellular motility

## Abstract

For tissues to spread, they must deform while staying intact. How spreading tissues balance flexibility with integrity is not yet well understood. Here, we show that keratin intermediate filaments adapt tissue mechanical resilience to the stresses arising in epithelial tissues during spreading. By analyzing the expansion of the enveloping cell layer (EVL) over the yolk cell in zebrafish embryos in vivo, we find that keratin network maturation in EVL cells is promoted by stresses building up within the spreading tissue. Through genetic interference and tissue rheology experiments, complemented by a vertex model with mechanochemical feedback, we demonstrate that stress-induced keratin network maturation in the EVL increases tissue viscosity, to prevent tissue rupture. Further, keratins are required in the yolk cell for mechanosensitive actomyosin network contraction and flow, the forces pulling the EVL. These dual mechanosensitive functions of keratins enable a balance between pulling force production and EVL mechanical resilience, ensuring uniform and robust tissue spreading.

## Abstract (web summary)

The contrasting roles of epithelial tissues in providing flexibility while maintaining integrity are not yet well understood. Here, the authors show that this need can be fulfilled by keratin intermediate filaments cross talking to other cytoskeletal machinery across the developing epithelium.

## Introduction

Epithelial cell layer spreading is a core feature of multiple developmental and disease-related processes. In *Drosophila* development, for instance, the spreading of the epidermis leads to the closure of an opening at the dorsal side of the embryo^1,2^. Likewise, in wound healing, the epidermal cell layer spreads, and fusion closes the wound^3,4^. Various cellular processes have been proposed to contribute to epithelial cell layer spreading, including cell spreading, cell migration, oriented cell division, and cell intercalation^5,6^. These processes can generate the mechanical forces driving active tissue expansion and determine the aptitude of tissues to undergo spreading.

**Fig. 1:**
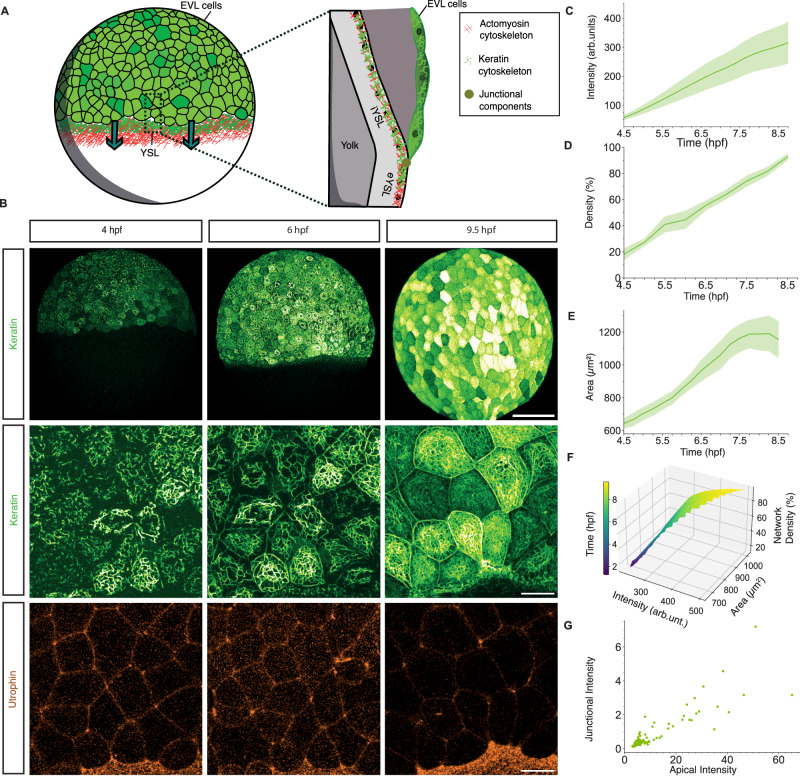
Keratin network maturation in the EVL. **A** Left: schematic representation of a zebrafish embryo at 60% epiboly stage (6 hpf) showing the expression and localization of keratin within the EVL and YSL (together with actin). Arrows mark the direction of EVL epiboly movements. Right: cross-section of the region outlined in the left panel marking the expression and localization of keratin, actin (within the YSL) and junctional components linking the margin of the EVL to the YSL. **B** Plot of maximum intensity projection images of keratin (top and middle rows) and actin (Utrophin; bottom row) expression in Tg*(krt18:Krt18-GFP)* (keratin) and Tg*(acbt2:Utrophin-mcherry)* (actin) embryos showing the progression of keratin expression within the EVL and YSL during epiboly (4–9.5 hpf). Scale bar: 100 µm (top row) and 25 µm (middle and bottom row). **C** Plot of averaged keratin intensity within the EVL in Tg*(krt18:Krt18-GFP)* embryos as a function of time during epiboly (4–9.5 hpf). *N* = 3 experiments, *n* = 5 embryos. Data are presented as mean line ± standard error of the mean (SEM) ribbon. **D** Plot of averaged density of the keratin network in Tg*(acbt2:Utrophin-mcherry, krt18:Krt18-GFP)* embryos as a function of time during epiboly (4–9.5 hpf). *N* = 3, *n* = 6 embryos. Data are presented as mean line ± SEM ribbon. **E** Plot of averaged apical cell area of individual EVL cells in Tg*(acbt2:Utrophin-mcherry, krt18:Krt18-GFP)* embryos as a function of time during epiboly (4–8.5 hpf). *N* = 3, *n* = 4 embryos. Data are presented as mean line ± SEM ribbon. **F** 3-dimensional (3D) plot of keratin intensity, network density, and EVL cell area as a function of time during epiboly. *N* = 3, *n* = 4 embryos. Data are presented as a surface whose width represents data spread, indicating their variability (standard deviation (SD) of density, intensity and area). **G** Plot of junctional versus apical keratin intensity measured in individual EVL cells in Tg*(acbt2:Utrophin-mcherry, krt18:Krt18-GFP)* embryos at 60% epiboly stage (6 hpf) *N* = 3, *n* = 3 embryos. Data are presented as scatterplot with points corresponding to individual cells. Source data are provided as a Source Data file.

Zebrafish embryo morphogenesis is initiated by the spreading of the blastoderm over the large yolk cell in a process named epiboly^7^. During epiboly, the EVL, a simple squamous epithelial cell layer, formed at the surface of the blastoderm, undergoes massive spreading to eventually engulf the entire yolk cell at the end of epiboly (Fig. 1A)^8,9^. EVL spreading has been shown to depend on the formation and contraction of a large actomyosin band positioned within a thin cytoplasmic layer at the surface of the yolk cell, the yolk syncytial layer (YSL)^10^. This actomyosin band within the YSL forms around the entire circumference of the yolk cell close to where the leading edge of the EVL contacts the YSL, and its contraction and flow are thought to generate the mechanical forces pulling the EVL over the yolk cell (Fig. 1A)^10,11^. Both active spreading of EVL cells and oriented EVL cell divisions have been implicated in facilitating EVL spreading by modulating EVL surface tension^12,13^. However, EVL morphogenesis not only relies on the generation and transmission of active forces within the EVL and YSL, but also on changes in the material properties of the tissue determining its deformation in response to such forces^14^. Yet, the molecular and cellular mechanisms that determine EVL material properties and how they are spatiotemporally coupled to changes in active force production remain unclear. Understanding these mechanisms requires a closer examination of the cytoskeletal components within epithelial cells, particularly those that have been implicated in modulating tissue material properties.

Keratin intermediate filaments are the most abundant and diverse cytoskeletal components in epithelial cells^15,16^. They form bundled filaments from keratin type I and type II heterodimers laterally associating in an antiparallel fashion into apolar tetramers that again align and anneal longitudinally into unit-length filaments^17,18^. Keratin filaments are semi-flexible, stable and highly elastic, different from the rather stiff and rigid actin and microtubule filaments^19,20^. They can self-assemble into intricate subcellular networks, the precise organization of which depends on their functional adaptation in different cell types^21^. Generally, the intracellular keratin network can organize into a rim network, supporting plasma membrane integrity and connecting desmosomal contacts, and a spoke network, surrounding the nucleus and transferring information from the cell exterior to the nucleus^22–24^. In polarized simple epithelia, such as the EVL, keratin networks are typically positioned near the apex where they are thought to function in resisting mechanical and chemical stresses and maintaining epithelial apicobasal polarity^25–27^. Keratins have been shown to be sensitive to mechanical forces by reorganizing and changing their mechanical properties upon stress application^19,28,29^. Yet, how keratin network mechanosensitivity functions in epithelial tissue morphogenesis remains unsettled^30,31^.

The role of epithelial keratins in development and disease is only beginning to be understood^32,33^. Keratin mutations can cause diseases that lower the resilience of epidermal tissues to mechanical stress^34^. Moreover, studies in mouse embryos have shown that keratins are required for trophectoderm specification and extra-embryonic tissue growth and expansion^30,31^. Here we show that keratin intermediate filaments are required for EVL spreading during zebrafish epiboly. They function in this process by balancing EVL tissue viscosity with the external pulling forces mediating EVL spreading. This balancing function of keratins enables the EVL to undergo uniform spreading without rupturing.

## Results

### Keratins are specifically expressed within the EVL and YSL during gastrulation

To explore the function of keratins during early zebrafish embryogenesis, we analyzed the expression of different keratins in embryos from early blastula to late gastrula stages (4.5–8.5 hpf). Previous studies have shown that 13 type I keratins and 3 type II keratins are specifically expressed within the developing EVL during this period^35,36^. To identify the temporal keratin expression profiles within the EVL during epiboly, we used RT-qPCR to map the expression of three keratin type II (*keratin 4, 5, 8*) and one keratin type I (*keratin 18*), abundantly expressed within the EVL^36,37^. We found all of these keratins to be expressed already at early blastula stages, with their expression continuously increasing until the end of gastrulation (Supplementary Fig. 1A). The expression was restricted to EVL progenitor cells at the blastoderm surface as evidenced by fluorescence in situ hybridization for *keratin 8* mRNA (Supplementary Fig. 1B). To determine the spatial distribution of keratins within the epibolizing embryo, we took advantage of *Tgkrt18:krt18-GFP* embryos expressing GFP-tagged keratin 18 under its endogenous promoter. Keratin 18 expression was first detected in EVL progenitor cells within the early gastrula (4.0 hpf) arranged in short, bundled and unconnected filaments located predominantly at the apical surface of these cells (Fig. 1B, Supplementary Video 1 and Supplementary Fig. 1C). Keratin 18 continued to be selectively expressed within EVL cells until mid-gastrulation (5.5 hpf), when some additional expression was also detected within the forming YSL directly adjacent to the place where the leading edge of the EVL contacts the YSL (Fig. 1B). During this period and continuing until the end of gastrulation, the apical network of keratin 18 filaments within EVL cells became increasingly dense and interconnected (Fig. 1B), as evidenced by a continuous increase in keratin 18-GFP intensity and network density between 4.5 and 8.5 hpf (Fig. 1C, D, and Supplementary Video 1). This increase in keratin network intensity and density was accompanied by EVL cells increasing the apical area during epiboly (Fig. 1E), suggesting a close temporal correlation between keratin network maturation and EVL cell spreading (Fig. 1F). Initially, keratin filaments were predominantly localized to the apical surface of the EVL cells (Fig. 1B and Supplementary Fig. 1C), but from 6 hpf onwards, additional keratin 18 expression was detected in a bundle-like configuration along the apical junction of EVL cells (Fig. 1B, G), consistent with previous observation of keratins showing both junctional (“rim”) and apical (“spoke”) localization^22,28,30,31^.

Finally, to check whether keratins in EVL cells can organize into networks different from t he keratin 18 network, we ubiquitous expressed a mCherry-tagged form of *keratin 4* by mRNA injection at the 1-cell-stage. Interestingly, keratin 4-mCherry, previously shown to form different keratin dimers than keratin 18^21^, was selectively expressed within EVL cells and its subcellular expression pattern entirely co-localized with the keratin 18 network (Supplementary Fig. 1D), suggesting that different keratin dimers are part of the same network within EVL cells. Of note, EVL cells typically showed different levels of keratin expression both on the mRNA and protein level, with cells initially expressing higher levels also showing an earlier network maturation (Fig. 1B, G and Supplementary Fig. 1B). This eventually resulted in the EVL at later stages of gastrulation being composed of cells displaying keratin networks at clearly different stages of maturation (Fig. 1B, G).

Collectively, these findings suggest that keratins are predominantly expressed within the EVL and adjacent YSL, forming an increasingly dense apical and junctional network from early blastula to late gastrula stages accompanying EVL cell spreading.

### Keratin network maturation is mechanosensitive

**Fig. 2:**
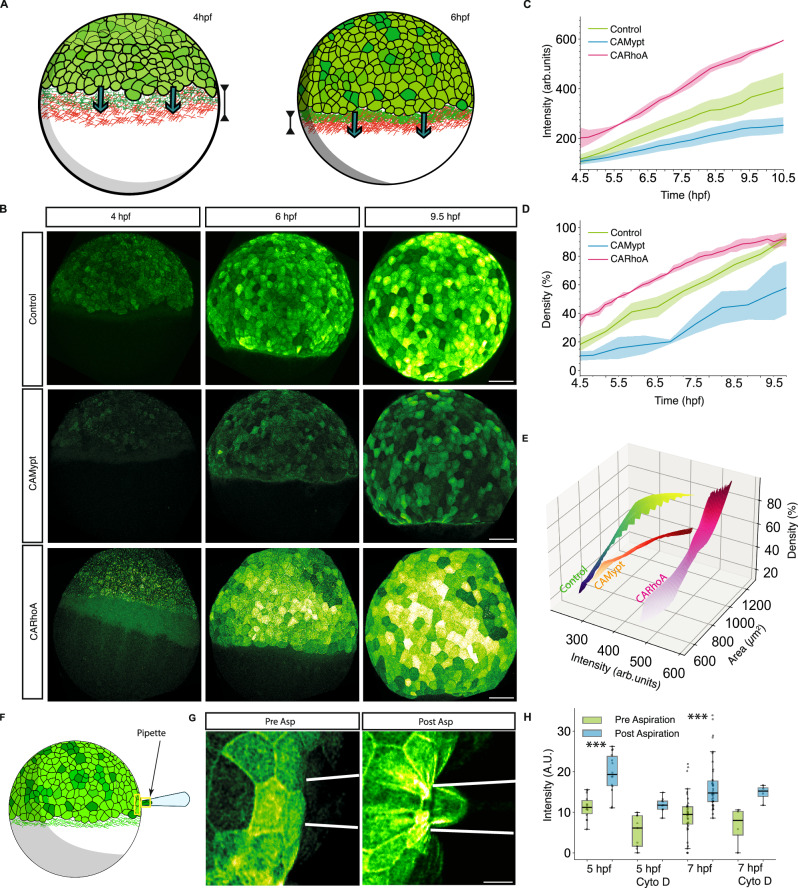
Pulling forces promote keratin expression within the EVL. **A** Schematic showing actomyosin contraction and flows within the YSL providing the mechanical forces pulling the EVL over the yolk cell during epiboly (for details see ref. ^10^) in a zebrafish embryo at 4 hpf (left) and 6 hpf (right). Green arrows, EVL epiboly movements; black arrows, actomyosin contraction within the YSL. **B** Maximum intensity projection images of keratin expression in representative Tg*(acbt2:Utrophin-mcherry, krt18:Krt18-GFP)* control embryos (YSL injection of 0.2% phenol red, top row) and embryos injected with 100 pg *CAMypt* (middle row) and 50 pg *CARhoA* (bottom row) into either the YSL at 3.3 hpf (*CAMypt*) or marginal cells at 3.3 hpf (*CARhoA*) at sphere stage (4 hpf, left column), shield stage (6 hpf, right column), and 90% epiboly stage (9.5hpf, right column). Scale bar: 100 µm. **C** Plot of average keratin intensity as a function of time (hpf) in Tg*(krt18:KrtGFP)* control (green, *N* = 3, n = 5 embryos), *CAMypt* (blue, *N* = 3, *n* = 5 embryos) and *CARhoA* mRNA injected embryos (pink, *N* = 3, *n* = 4 embryos) as described in (**B**). Data are presented as mean line ± standard error of the mean (SEM) ribbon. **D** Plot of average density of keratin network as a function of time (hpf) in Tg*(actb2:Utrophin-mcherry, krt18:Krt18-GFP)* control (green, *N* = 3, *n* = 6 embryos), *CAMypt* (blue, *N* = 3, *n* = 4 embryos) and *CARhoA* mRNA injected embryos (pink, *N* = 3, *n* = 3 embryos) as described in (**B**). Data are presented as mean line ± SEM ribbon. **E** 3-dimensional (3D) plot of keratin intensity, network density and EVL cell area as a function of time (hpf) in Tg*(actb2:Utrophin-mcherry, krt18:Krt18-GFP)* control (viridis, *N* = 3, *n* = 3 embryos), *CAMypt* (orange, *N* = 3, *n* = 3 embryos) and *CARhoA* mRNA injected embryos (pink, *N* = 3, *n* = 3 embryos) as described in (**B**). Data are presented as a surface whose width represents data spread, indicating their variability (standard deviation (SD) of density, intensity and area). **F** Schematic showing pipette aspirations of the EVL of a 70% epiboly (7 hpf) stage embryo where the regions of interest within the pipette and outside of it are marked as yellow boxes. **G** Maximum intensity projection images of keratin localization and intensity within the EVL before (left) and after (right) aspiration with a pipette in a representative Tg*(krt18:KrtGFP)* embryo. White lines outline the boundary of the pipette. Scale bar: 10 µm. **H** Box plot overlaid with scatter of keratin intensity within the pipette before (green) and after (blue) EVL aspiration in control (*N* = 4, *n* = 33 embryos), and Cytochalasin D (CytoD; 25 nM)-treated (*N* = 4, *n* = 25 embryos) Tg*(actb2:Utrophinmcherry, krt18:Krt18GFP)* embryos at 5 and 7 hpf. Box plots show the distribution with median (center line), interquartile range (box), and whiskers extending to values within 1.5 × IQR. (*p* values: ***<0.001; ANNOVA C(control versus CytoD treatment) = 3.883936e-05, ANNOVA C(pre- versus post-aspiration) = 3.045997e-14, ANNOVA interaction of C(stage)xC(control versus CytoD treatment) = 3.045997e-14). Source data are provided as a Source Data file.

EVL epiboly movements are driven by a large actomyosin cable forming within the YSL and pulling the margin of the EVL over the yolk cell^10^. Given that keratin networks can reorganize under stress^29,38^, we speculated that the observed maturation of the keratin network within EVL cells might be facilitated by EVL tissue tension building up during the course of epiboly^10,39^. To test this possibility, we analyzed whether and how EVL network maturation is affected in embryos where EVL tension is either increased or decreased. To modulate EVL tension, we expressed a constitutive active form of RhoA (CARhoA) specifically within the YSL, promoting YSL actomyosin contractility and pulling, or a constitutive active form of Myosin phosphatase (CAMypt) leading to reduced YSL contractility and pulling^10,11^. In CARhoA-expressing embryos, keratin 18 expression intensity and network density in EVL cells prematurely increased during the course of epiboly, while in CAMypt-expressing embryos, keratin expression and network maturation were delayed (Fig. 2A–D, Supplementary Videos 2–5 and Supplementary Fig. 2B). This keratin mechanosensitivity was detectable both when network maturation was analyzed as a function of developmental time or degree of epiboly progression (Fig. 2A–D and Supplementary Fig. 2B, C), suggesting that the effect of EVL tension on keratin network maturation is not just a secondary consequence of changes in EVL epiboly movements (Supplementary Fig. 2D).

To more directly assess the effect of EVL tension on keratin network maturation, we locally increased EVL tension in 5 and 7 hpf embryos by aspirating the EVL using a micropipette and analyzing resultant changes in EVL network maturation. We found that keratin filaments showed increased accumulation in EVL cells upon aspiration (Fig. 2F–H, Supplementary Video 8 and Supplementary Fig. 2F), further supporting the notion that keratin network maturation within the EVL cells is promoted by EVL tension building up during the course of epiboly.

To investigate the mechanisms underlying keratin network mechanosensitivity, we examined whether keratin mRNA expression within the EVL is responsive to reduced pulling forces from the YSL. Using qPCR, we compared *keratin 4, 5, 8,* and *18* expression levels in control embryos and embryos expressing CAMypt within the YSL, which attenuates actomyosin contraction-mediated pulling on the EVL^10^. Notably, this comparison revealed no significant differences in *keratin 4, 5, 8,* and *18* expression (Supplementary Fig. 2I), arguing against a force-dependent transcriptional regulation of keratin within the EVL.

Interestingly, in our micropipette aspiration assay, increased keratin expression in the EVL was spatiotemporally accompanied by a marked enhancement of actin network assembly (Supplementary Fig. 2E, F). Given the well-established mechanosensitivity of the actomyosin cytoskeleton and its known physical interactions with the keratin network^11,21,40,41^, this observation raises the possibility that keratin mechanosensitivity in the EVL may depend on actin network dynamics.

To test this hypothesis, we treated embryos with low concentrations of Cytochalasin D (CytoD) to impair actin network assembly in EVL cells and assessed keratin upregulation upon micropipette aspiration. Strikingly, CytoD-treated embryos showed a markedly reduced keratin response to aspiration (Fig. 2H), suggesting that proper actin network assembly is required for keratin mechanosensitivity in the EVL.

To more directly assess whether actomyosin contractility affects keratin network assembly, we injected CAMypt mRNA into a single blastomere at the 64-cell stage to generate EVL clones with reduced contractility. We found that downregulation of actomyosin contractility resulted in a corresponding decrease in keratin network assembly within these clones (Fig. 2A–D and Supplementary Fig. 2G, H), supporting a causal role of actomyosin tension in promoting keratin assembly.

Together, these findings demonstrate that the keratin cytoskeleton in EVL cells is mechanosensitive and that this mechanosensitivity is dependent on the mechanosensitive assembly of the actin network.

### Keratins regulate EVL tissue properties

Keratins have previously been implicated in resisting mechanical stresses in epithelial cells. Thus, the mechanosensitive coupling between EVL keratin network maturation and pulling force generation within the YSL might constitute a mechanism to protect the EVL tissue against excessive deformations by mechanically strengthening it proportionally to the force exerted on it.

**Fig. 3:**
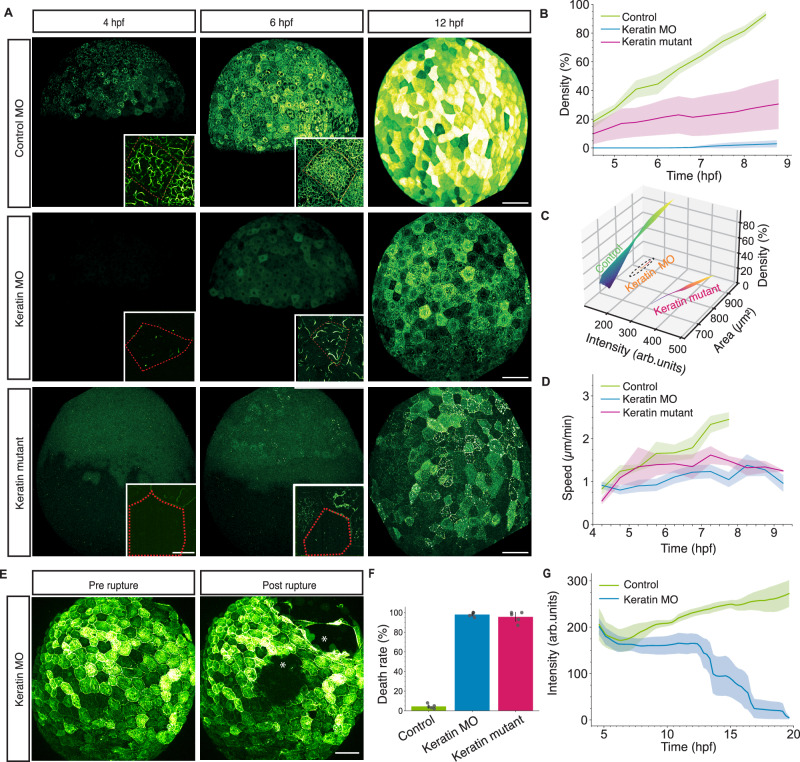
Loss of keratin expression diminishes EVL epiboly movements. **A** Maximum intensity projection images of keratin expression in representative Tg*(krt18: Krt18GFP)* embryos at sphere stage (4 hpf, left column), shield stage (6phf, middle column) and bud stage (10 hpf, right column) - with insets on the right lower corner showing single cells with their boundary marked by a red line - injected at the one-cell stage with 2 ng control MO (top row), 1 ng *keratin 4* plus 1 ng *keratin 8* MO (middle row), or with TraCr *keratin 4* and *keratin 8* gRNA (*keratin 4/8* crispant F0; bottom row). Scale bar: 100 µm (main panel) and 10 µm (inset). **B** Plot of averaged density of keratin network in individual EVL cells as a function of time (hpf) during epiboly in Tg*(actb2:Utrophinmcherry, krt18:Krt18GFP)* embryos injected at the one-cell stage with 2 ng control MO (top row; green, *N* = 4, *n* = 4 embryos), 1 ng *keratin 4* plus 1 ng *keratin 8* MO (middle row; blue, *N* = 4, *n* = 4 embryos), or with TraCr *keratin 4* and *keratin 8* gRNA (*keratin 4/8* crispant F0; bottom row; pink, *N* = 2, *n* = 6 embryos). Data are presented as mean line ± standard error of the mean (SEM) ribbon. **C** 3-dimensional (3D) plot of keratin intensity, network density and area of EVL cells as a function of time (hpf) during epiboly in Tg*(actb2:Utrophinmcherry,krt18:Krt18GFP)* embryos injected at the one-cell stage with 2 ng control MO (top row; green, *N* = 4, *n* = 4 embryos), 1 ng *keratin 4* plus 1 ng *keratin 8* MO (middle row; orange, *N* = 4, *n* = 4 embryos), or with TraCr *keratin 4* and *keratin 8* gRNA (*keratin 4/8* crispant F0; bottom row; pink, *N* = 2, *n* = 6 embryos). Data are presented as a surface whose width represents data spread, indicating their variability (standard deviation (SD) of density, intensity and area). **D** Plot of EVL epiboly movement speed as a function of time (hpf) during epiboly starting at sphere stage (4 hpf) until late epiboly stages (9 hpf) in Tg*(actb2: Utrophin-mcherry, krt18:Krt18GFP)* embryos injected at the one-cell stage with 2 ng control MO (top row; green, *N* = 3, *n* = 4 embryos), 1 ng *keratin 4* plus 1 ng *keratin 8* MO (middle row; blue, *N* = 3, *n* = 4 embryos), or with TraCr *keratin 4* and *keratin 8* gRNA (*keratin 4/8* crispant F0; bottom row; yellow, *N* = 2, *n* = 4 embryos). Data are presented as mean line ± SEM ribbon. **E** Maximum intensity projection images of the EVL at 13.5 hpf in Tg(*actb2: Utrophin-mcherry, krt18:Krt18GFP)* embryos injected with 1 ng *keratin 4* plus 1 ng *keratin 8* MO at the one-cell stage before (left) and after rupture (right). Scale bar:100  µm. **F** Bar plot of death rate with overlaid scatter points representing individual measurements in Tg(*actb2:Utrophinmcherry, krt18:Krt18GFP)* embryos injected at the one-cell stage with 2 ng control MO (green, *N* = 4, *n* = 4 embryos), 1 ng *keratin 4* plus 1 ng *keratin 8* MO (blue, *N* = 4, *n* = 4 embryos), or with TraCr *keratin 4* and *keratin 8* gRNA (*keratin 4/8* crispant F0; yellow, *N* = 2, *n* = 6 embryos). Data are represented as bar plots showing mean values, with error bars representing standard deviation (SD). **G** Plot of average E-cadherin intensity as function of time in control MO (green, *N* = 3, *n* = 6) and 1 ng *keratin 4* plus 1 ng *keratin 8* MO (blue, *N* = 3, *n* = 5 embryos) injected Tg(*cdh1-mlanYFP)xt17* embryos. Data are presented as mean line ± SEM ribbon. Source data are provided as a Source Data file.

To test this possibility, we knocked down/out the expression of the two keratin type II genes (*keratin 4* and *8*) primarily expressed within EVL cells^36^, reasoning that in the absence of keratin type II expression, no keratin dimers can be formed within the EVL and thus keratin network formation should be defective. Ubiquitous knock-down/out of *keratin 4* and *8* expression by using morpholinos and Crispr-Cas9 or interference with keratin network formation by overexpressing a dominant negative version of *keratin 18* (DNkeratin18)^41^, led to strongly diminished keratin 18 expression and network formation within EVL cells (Fig. 3A–C, Supplementary Videos 6–7 and Supplementary Fig. 3A, B). Loss of the keratin network in all these perturbations led to delayed EVL epiboly movements (Fig. 3D, Supplementary Videos 6 and 7 and Supplementary Fig. 3C) and frequent rupturing of the EVL towards the end of gastrulation, ultimately causing embryo lethality (Supplementary Fig. 3E, F). This phenotype could be partially rescued by co-injecting the *keratin 8* morpholino - eliciting an epiboly phenotype similar but slightly weaker than the phenotype observed when injecting both *keratin 4* and *8* morpholinos - with *keratin 8* mRNA not recognized by the morpholino (Supplementary Fig. 3F–H, Supplementary Video 8). Rupture of the EVL in keratin deficient embryos was typically preceded by a reduction in junctional localization of E-cadherin (adherens junctions), Occludin b (tight junctions), and junctional plakoglobin a (desmosomal junctions) (Fig. 3G, Supplementary Videos 12–14 and Supplementary Fig. 3D, E), suggesting that keratins are essential for maintaining EVL tissue integrity by enabling proper formation and stabilization of cell-cell junctions between EVL cells.

**Fig. 4:**
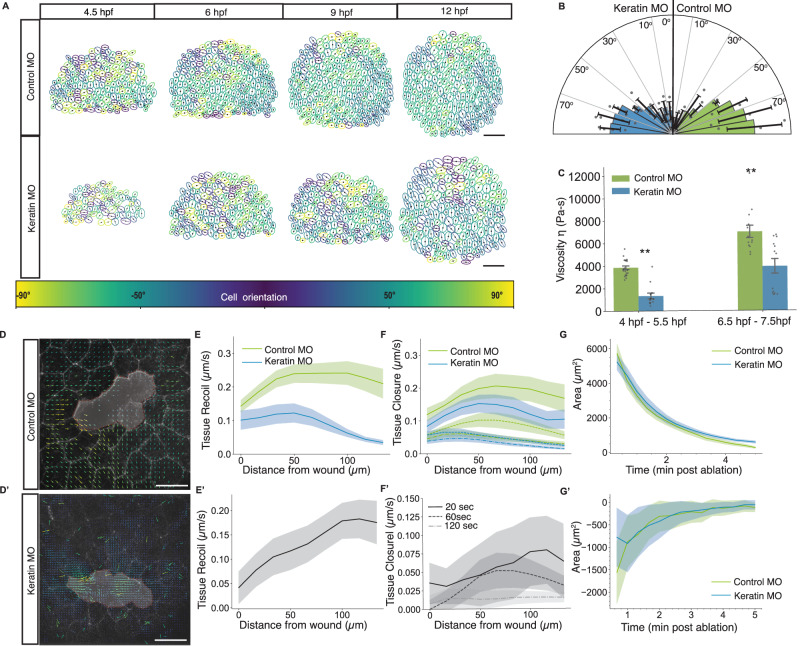
Mechanical force percolation within the EVL is dependent on keratin expression. **A** Plots of EVL cell orientations with ellipses representing shape descriptors (long and short axis) of individual EVL cells with the line in the middle marking the orientation of the long axis at consecutive stages during epiboly (4.5, 6, 9, 12 hpf) in curvature corrected Tg*(actb2:Utrophin-mcherry, krt18:Krt18GFP)* embryos injected at the one-cell stage with 2 ng control MO (top row) or 1 ng *keratin 4* plus 1 ng *keratin 8* MO (middle row). Each cell is color-coded according to the orientation of the axis as shown in the color bar (viridis) at the bottom (Green: AV axis orientation, blue: dorsoventral/DV orientation). Scale Bar: 100 µm. **B** Rose plot overlaid with scatter of bin means of EVL cell orientations in Tg*(actb2:Utrophinmcherry,krt18:Krt18GFP)* embryos at 6 hpf injected at the one-cell stage with 2 ng control MO (*N* = 4, *n* = 93 cells) or 1 ng *keratin 4* plus 1 ng *keratin 8* MO (*N* = 4, *n* = 84 cells). **C** Bar plots with overlaid scatter points representing individual embryo measurements of tissue viscosity measured at the EVL margin using micropipette aspiration at 4–5.5 hpf and 6.5–7.5 hpf in Tg*(actb2: Utrophin-mcherry, krt18:Krt18GFP)* embryos injected at the one-cell stage with 2 ng control MO (*N* = 5, *n* = 37 embryos) or 1 ng *keratin 4* plus 1 ng *keratin 8* MO (C; *N* = 5, *n* = 33 embryos). Data are represented as bar plots showing mean values, with error bars representing SD. (*p* value: **<0.01; *t*-test *p*(4-5.5.hpf) = 2.797231e-12 and  *p* (6.5-7.5hpf) = 1.792669e-08) **D** Representative quiver plots of EVL tissue recoil flow velocities after cell ablation/wounding in Tg*(actb2:Utrophinmcherry)* embryos at 6 hpf injected with 2 ng control MO (top) or 1 ng *keratin 4* plus 1 ng *keratin 8* MO (bottom). The arrows show the local velocity colored according to the magnitude (viridis). Scale bar: 25 µm. **E** Plot of averaged EVL tissue recoil velocity directly following cell ablation plotted as a function of distance from the wound center (0 µm) in Tg*(actb2:Utrophinmcherry)* embryos injected with 2 ng control MO (green, *N* = 4, *n* = 23 embryos) or 1 ng *keratin 4* plus 1 ng *keratin 8* MO (blue, *N* = 4, *n* = 17 embryos) immediately after EVL cell ablation. Data are presented as mean line ± standard error of the mean (SEM) ribbon. **E’** Plot of the difference of averaged EVL tissue recoil velocity directly following ablation plotted as a function of distance from the wound center (0 µm) in Tg*(actb2:Utrophinmcherry)* embryos injected with 2 ng control MO versus 1 ng *keratin 4* plus 1 ng *keratin 8* MO. Data are presented as mean line ± SEM ribbon. **F** Plot of averaged EVL tissue flow velocity during wound closure after EVL cell ablation plotted as a function of distance from the wound center (0 µm) in Tg*(actb2:Utrophinmcherry)* embryos injected with 2 ng control MO (green, *N* = 4, *n* = 23 embryos) or 1 ng *keratin 4* plus 1 ng *keratin 8* MO (blue, *N* = 4, *n* = 17 embryos) at successive timepoints after EVL cell ablation (20 s, solid; 60 s, dotted; 120 s, dotted line with points). Data are presented as mean line ± SEM ribbon. **F’** Plot of the difference of averaged EVL tissue flow velocity during wound closure after EVL cell ablation plotted as a function of distance from the wound center (0 µm) in Tg*(actb2:Utrophinmcherry)* embryos injected with 2 ng control MO versus 1 ng *keratin 4* plus 1 ng *keratin 8* MO at successive timepoints after the ablation (20 s, solid; 60 s, dotted; 120 s, dotted line with points). Data are presented as mean line ± SEM ribbon. **G** Plot of wound area as a function of time after EVL cell ablation in Tg*(actb2:Utrophinmcherry)* embryos injected with 2 ng control MO (green, *N* = 4, *n* = 23 embryos) or 1 ng *keratin 4* plus 1 ng *keratin 8* MO (blue, *N* = 4, *n* = 17 embryos). Data are presented as mean line ± SEM ribbon. (**G’**) Plot of the change of wound area as a function of time after EVL cell ablation in Tg*(actb2:Utrophinmcherry)* embryos injected with 2 ng control MO (green, *N* = 4, *n* = 23 embryos) or 1 ng *keratin 4* plus 1 ng *keratin 8* MO (blue, *N* = 4, *n* = 17 embryos). Data are presented as mean line ± SEM ribbon. Source data are provided as a Source Data file.

To understand how keratins function in EVL epiboly movements, we analyzed changes in EVL cell shapes and rearrangement in response to the pulling forces from the YSL during epiboly. Comparing wild-type with *keratin 4/8* morphant embryos revealed that EVL cells in wild-type embryos coordinately elongated along the animal-vegetal (AV) axis of the gastrula, the axis of EVL spreading, while no such coordinated cell elongation was detectable in morphant embryos (Fig. 4A, B). This points at the possibility that tissue material properties, determining force transduction and mechanical resilience, might be altered by keratin network formation.

To investigate this possibility, we measured the material properties of EVL tissue at early and mid-gastrulation (5 and 7 hpf) using micropipette aspiration (Supplementary Fig. 4E)^42,43^. Analysis of the flow profile of the EVL into the pipette revealed a linear response, consistent with viscous properties, which allowed us to assess the viscosity of EVL tissue. Comparing the viscosity of EVL tissue between early and mid-gastrulation stages showed a significant increase in viscosity at mid-gastrulation, coinciding with the maturation of the keratin network (Fig. 4C, Supplementary Video 9 and Supplementary Fig. 2F). This suggests that keratin network maturation during epiboly contributes to the increase in EVL tissue viscosity. To test this function of keratins more directly, we performed EVL micropipette aspiration experiments in wild-type and *keratin 4/8* morphant embryos. At early gastrulation, when differences in keratin network formation between wild-type and morphant embryos were still relatively small (Fig. 1B), we observed a slight reduction in viscosity in *keratin 4/8* morphant embryos compared to wild-type (Fig. 4C). In contrast, at mid-gastrulation (Fig. 4C), when the differences in keratin network organization between morphant and wild-type embryos became more pronounced (Fig. 1B), viscosity was significantly reduced in the morphant embryos. This supports the idea that keratin network maturation enhances EVL tissue viscosity.

To test whether this effect is due to keratin expression specifically within EVL cells, we knocked down *keratin 4/8* expression within the YSL by injection of *keratin 4/8* morpholinos directly into the forming YSL at the high (3.5 hpf) stage. Interestingly, this did not cause any detectable changes in coordinated EVL cell elongation along the AV axis compared to wild-type embryos, suggesting that keratins control EVL cell shape changes in a tissue-autonomous manner (Supplementary Fig. 4A, B).

To further challenge this conclusion, we developed an EVL wound healing assay, allowing us to monitor autonomous EVL spreading during wound closure^44,45^. For wounding the EVL, we ablated 3–5 neighboring cells at random positions within the EVL in mid-gastrulation stage embryos (7 hpf) and observed how the coordinated spreading and movements of the neighboring cells extruded and replaced the ablated cells. In control embryos upon ablation, a supracellular actin cable formed at the leading edge of EVL cells neighboring the ablated cells (Fig. 4D, Supplementary Videos 10, 11). This was accompanied by the highly coordinated movement and spreading of EVL cells towards the site of cell ablation, eventually leading to the extrusion of the ablated cells and closure of the wounding site (Fig. 4F-F’). During this process, not only did leading-edge EVL cells elongate and move toward the wounding site, but cells located further away from the ablation site also responded, resulting in highly coordinated long-range tissue movements detected by particle image velocimetry (PIV) flow analysis (Fig. 4D–G). In contrast, EVL tissue movements were largely restricted to EVL cells directly neighboring the wounding site in *keratin 4/8* morphant and crispant embryos (Fig. 4D–G, Supplementary Video 11 and Supplementary Fig. 4C, D). Similar differences in the spatial extent of tissue flows between wild-type and morphant/crispant embryos were observed when analyzing tissue recoil immediately following cell ablation, prior to initiation of wound closure by formation of the supracellular actin cable at the EVL leading edge (Supplementary Fig. 4C, D). Collectively, these findings support the notion that keratin expression within the EVL increases tissue viscosity, thereby extending the hydrodynamic length over which the EVL tissue deforms in response to forces applied at its margin.

### Keratins are required for mechanosensitive actomyosin contraction within the YSL

Given that keratin expression within the EVL is mechanosensitive, we hypothesized that this behavior might constitute a feedback mechanism balancing EVL tissue viscosity with the forces pulling on its margin, thereby setting the rate of EVL epiboly movements. To address this hypothesis, we developed a vertex model^46,47^ of the EVL, enabling us to relate EVL tissue spreading dynamics to mechanosensitive keratin expression. Mechanical heterogeneity in epiboly has already been established within the context of a vertex model^48^. To this end, we wrote the energy $U={\sum }_{i}\frac{{\Gamma }_{i}}{2{A}_{0}}{({A}_{i}-{A}_{i,0})}^{2}+\frac{{\varGamma }_{i}}{2}{({P}_{i}-{P}_{i,0})}^{2}$ which penalizes deviations of the areas ${A}_{i}$ and perimeters ${P}_{i}$ of the cells $i$, with cell stiffness constants ${\varGamma }_{i}$, from their target values ${A}_{i,0}$ and ${P}_{i,0}$, respectively. In order to make the tissue viscoelastic, we relaxed the target area ${A}_{i,0}$ over a characteristic viscous relaxation timescale ${\tau }_{i}$ following ${\tau }_{i}\frac{{{dA}}_{i,0}}{{dt}}=-({A}_{i,0}-{A}_{i})$, while enforcing a minimum area ${A}_{0}$, and scaled ${P}_{i,0}$ to work at a constant shape parameter ${s}_{0}=\frac{{P}_{i,0}}{\sqrt{{A}_{i,0}}}=3.72$, a value where the tissue is predicted to be still rigid^46^. Our model incorporates tissue spreading in 3D implicitly through a conserved cell volume ${V}_{0}={h}_{i}{A}_{i}$, where ${h}_{i}$ is the height of the cell (see SI, section 3 for details).

**Fig. 5:**
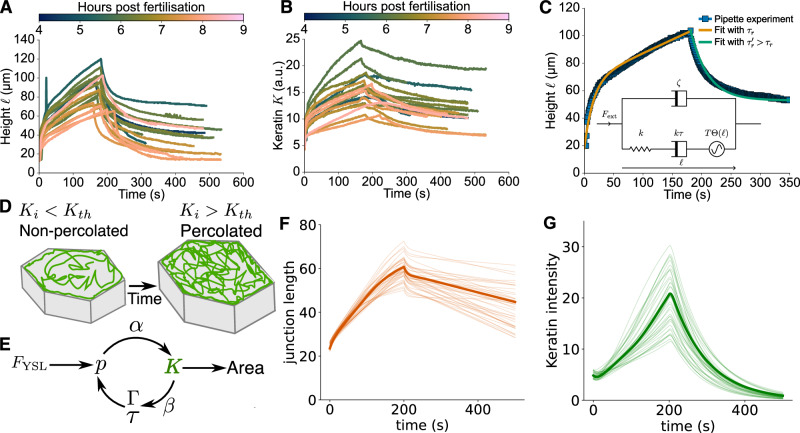
Model of tissue with a mechanosensitive feedback loop for keratin. **A** Plot of extension length of the aspirated EVL tissue in Tg(*krt18:Krt18GFP)* embryos as a function of time upon micropipette aspiration. Colormap represents developmental time starting at the beginning of epiboly (4 hpf, blue) to later stages (8 hpf, orange). **B** Plot of keratin intensity measured inside the pipette in the aspirated EVL tissue in Tg(*krt18:Krt18GFP)* embryos as a function of time upon micropipette aspiration. Colormap represents developmental time starting at the beginning of epiboly (4 hpf, blue) to later stages (8 hpf, orange). **C** Representative plot of extension length as a function of time fit to the modified viscoelastic Maxwell model (inset, schematic representing the model) used to measure parameters for the vertex model. **D** Schematic representing a singular model EVL cell showing keratin filaments (green) at two distinct states of the network activity, dependent on the keratin ${K}_{i}$ formation: unpercolated (left, ${K}_{i} < {K}_{{th}}$, mechanically inactive) and percolated (right, ${K}_{i} > {K}_{{th}}$, mechanically active) with ${K}_{{th}}$ being the threshold value of *K* above which the keratin mechanochemical feedback loop becomes activated. **E** Schematic diagram of the mechanochemical feedback loop built into the model representing the feedback from pulling forces from the YSL (${F}_{{YSL}}$) stretching the tissue leading to increased pressure $p$ in the tissue that enhances keratin $K$ formation. This increase in keratin $K$ feedback into the area $A$ of the cells and increases tissue stiffness $\varGamma$ and relaxation time $\tau$. This increase in tissue stiffness $\varGamma$ and relaxation time $\tau$, in turn, feeds back on the pressure $p$ in the tissue. **F** Plot of model aspirated tissue heights as a function of time measured in the mean field model. Mean tissue height (dark red line) is represented as a solid line over curves representing estimated parameter variability shown as lines with higher transparency (light red lines). **G** Plot of model keratin intensities in aspirated tissues as a function of time in the mean field model. Mean keratin intensity (dark green line) is represented as a solid line over curves representing estimated parameter variability shown as lines with higher transparency (light green lines). Source data are provided as a Source Data file and in the Data Archive.

We next incorporated keratin into our model (Fig. 5D). As illustrated in Fig. 1B, keratin forms an intracellular network that is initially floppy, but reaches a percolation threshold at approximately ${K}_{{th}}=150$. Capturing the full complexity of its behavior would require simulating the coupled dynamics of actin and keratin networks, including keratin polymerization and depolymerization as well as actomyosin contractility at the subcellular level. To simplify, we instead implemented a mesoscale approximation in which the keratin network is acting in parallel with other mechanical components of the cell. Above the percolation threshold, keratin therefore contributes proportionally to both the cell’s effective stiffness and relaxation time with ${\varGamma }_{i}=\varGamma (1+\beta \varDelta {K}_{i})$ and ${\tau }_{i}=\tau (1+\beta \varDelta {K}_{i})$, respectively, where $\varDelta {K}_{i}=\max \left(0,{K}_{i}-{K}_{{th}}\right)$ and $\beta$ is a mechanical feedback constant.

To include keratin mechanosensitivity, we further assumed that its assembly from the cytosol increases with the stress applied on the tissue. To account for this, we used a simple, linear model of the evolution of the keratin concentration ${\tau }_{K}\frac{{{dK}}_{i}}{{dt}}=\alpha \,\max (0,{p}_{i})-{K}_{i}$, where keratin dissociates with time scale ${\tau }_{K}$ and $\alpha$ is a coupling constant (note that other nonlinear rheology models have also been used to model the effect of keratin^29^). Mechanosensitivity in our model arises through biaxial stress, or equivalently, the in-plane pressure ${p}_{i}$ defined as ${p}_{i}=\frac{1}{{V}_{0}}(\frac{{\varGamma }_{i}}{{A}_{0}}({A}_{i}-{A}_{i,0}){A}_{i}+{\varGamma }_{i}({P}_{i}-{s}_{0}\sqrt{{A}_{i,0}}){P}_{i})$ (see SI, section 3). This formulation captures the pressure response arising from deviations in both cell area and perimeter relative to their preferred values. In this framework, EVL spreading corresponds to a substantial increase in cell area ${A}_{i}$, accompanied by dramatic thinning of individual cells. At steady state, this mechanosensitive feedback yields a linear relationship between keratin concentration and pressure, expressed as ${K}_{i}=\alpha {P}_{i}$ under the assumption of positive pressure. A summary of this mechanochemical feedback loop is presented in Fig. 5E.

To estimate the parameters for simulating EVL wound closure and epiboly, we used the EVL aspiration experiments described above (Fig. 5A, B, Figs. 2F–H and 4C), where we took the initial and pulled states as a proxy for the tissue at low and high keratin expression, respectively. In a first step, we fitted the low-keratin initial response and the high-keratin release curves separately for tissue height and keratin expression using a modified viscoelastic Maxwell model, allowing us to obtain estimates of the dissociation timescale ${\tau }_{K}$, pressure coupling $\alpha$, cell stiffness constant $\varGamma$, and relaxation timescale $\tau$ (Fig. 5C, Supplementary Fig. 7A–D and SI section 1). This model seeks to explicitly understand the local rheology at the length scale of a couple of cells and the time scale of minutes and is mathematically equivalent to the standard aspiration model of tissue spheroids^42,48^(see SI section 2 for a detailed derivation).

In a second step, we developed a mean-field version of the full model that includes feedback (see SI section 5). We used this framework to compute tissue height and keratin expression in aspirated wild-type EVL tissue (Fig. 5F, G and Supplementary Fig. 9A–D), and to estimate the remaining coupling constant, $\beta$, which represents the mechanical contribution of keratin. We obtained $\beta=0.005$, such that increasing keratin levels from ${K}_{i}=0$ to ${K}_{i}=550$, with a percolation threshold ${K}_{{th}}=150,$ results in a 2-fold increase of $\varGamma$ and $\tau$ (Supplementary Fig. 9G,H). This parameter choice yields consistency with both the pipette aspiration measurements and the experimentally observed viscosity ratio between keratin-deficient and control embryos (Fig. 4C). The full set of fitted parameters is provided in Table S1.

For keratin deficient model tissues, we instead set $\beta=0$, thereby eliminating any mechanical contribution from keratin. This choice, rather than the experimentally more realistic scenario $\alpha=0$ (corresponding to the absence of keratin production), was motivated by the ability to monitor the role of feedback arising from keratin expression while keeping the feedback loop formally open. Aside from this distinction, the two parameter choices ($\beta=0$ and $\alpha=0$) are mathematically identical.

**Fig. 6:**
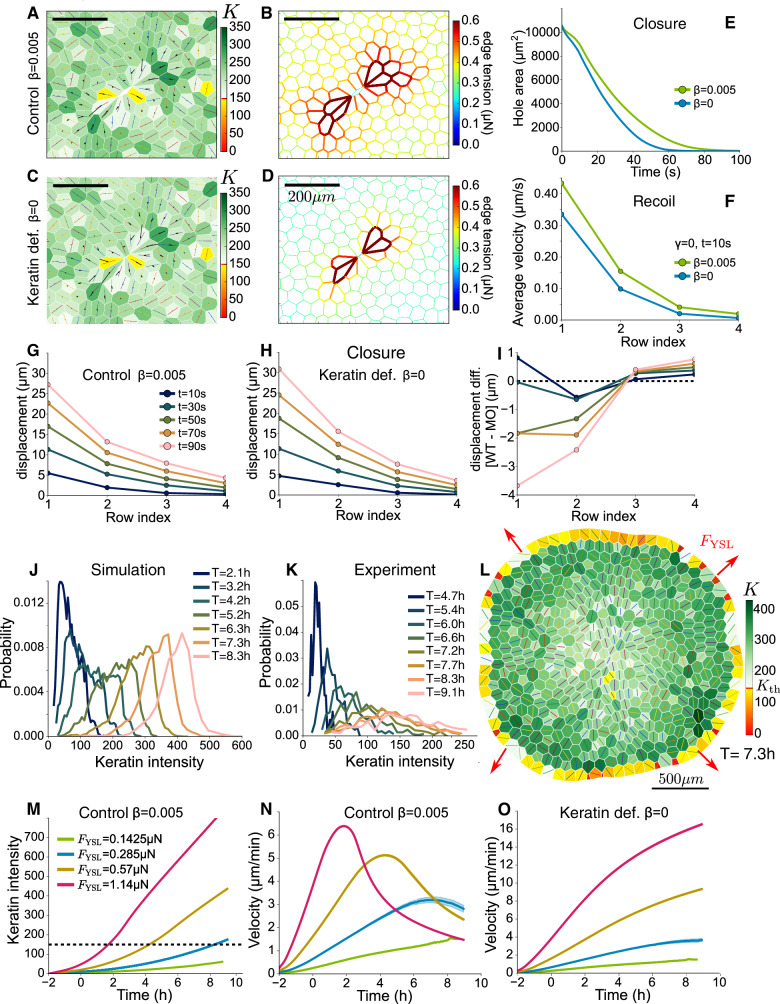
Vertex model of the EVL for tissue ablation and epiboly in the presence and absence of keratin mechanosensitivity. **A** Representative model tissue ablation showing keratin expression at $t=70s$ in an EVL model tissue with periodic boundary conditions and mechanochemical feedback $(\beta=0.005)$. At $t=0s$ 6 cells are removed to create a hole at the center of the tissue. A tension of 2 µN is applied on the edges surrounding the removal site to simulate a wound healing process. Colormap on the side represents the keratin intensities of the cells as a function of keratin $K$ with percolated keratin network (${K}_{i} > {K}_{{th}}$) appearing in green and unpercolated (${K}_{i} < {K}_{{th}}$) in red-yellow. Orientation of the cell elongation is shown as a line through the cell center, and total displacement vectors following cell removal (wounding) are shown as arrows. **B** Plot of edge tension at $t=70s$ around the ablated wound site in control ($\beta=0$.005) EVL model tissue. Colormap on the right represents the edge tension. **C** Tissue ablation for keratin deficient $(\beta=0)$ EVL model tissues, otherwise as in (**A**). **D** Plot of edge tension for keratin deficient $(\beta=0)$ EVL model tissues, otherwise as in (**A**). **E** Plot of wound area as a function of time after ablation in keratin-deficient ($\beta=0$; blue) and wild-type with ($\beta=0$.005; green) EVL model tissues. **F** Recoil velocity at $t=10s$ in control ($\beta=0$.005; green) and keratin deficient ($\beta=0$; blue) EVL model tissues after wounding, simulated as in (**A**) but without tension on the edges surrounding the cell removal site, representing the initial absence of the actomyosin cable during tissue recoil. Rows are counted outward from the (asymmetric) initial wound shape. **G**–**I** Total displacement of cells from their initial positions during simulated wound closure (with tension on the edges surrounding the cell removal site) as a function of time and distance from the wound counted in cell rows outward from the initial wound. **G** Control EVL model tissue with $\beta=0$.005. **H** Keratin deficient EVL model tissue with $\beta=0$. **I** Difference between control and keratin deficient simulations. **J** Histograms of keratin intensity distributions in simulated tissues as a function of simulated developmental time shown as a colormap of the lines (2.1 h navy to 8.3 h rose) **K** Histograms of keratin intensity distributions observed within the individual EVL cells in a representative Tg*(krt18: Krt18GFP)* embryo as a function of development time during epiboly shown as a colormap of the lines (4.7 hpf navy to 9.1 hpf rose). **L** Representative vertex model of the EVL tissue at mid epiboly depicting intensity of keratin $K$ (green, percolated and red-yellow, unpercolated) and the orientation of the cell elongation shown as a line through the cell center. The pulling force by the YSL applied at the edge is represented as red arrows pointing outwards. Stage of pulling corresponds to approximately 7.3 hpf in the zebrafish developing embryo. **M** Plot of mean keratin intensity in the model EVL tissue as a function of time at increasing pulling forces from the YSL ${F}_{{YSL}}\,$(color-coded). The threshold level of keratin ${K}_{{th}}$ is represented as a dotted line above which the mechanochemical feedback becomes active in the model. **N** Plot of mean tissue edge velocity in the presence of wild-type pulling force ${F}_{{YSL}}$ as a function of time in wild-type control $\beta=0$.005 EVL model tissues. **O** Plot of mean tissue edge velocity in keratin deficient $(\beta=0)$ EVL model tissues. Source data are provided in the Data archive.

Using these parameters in our extended vertex model (see simulation library)^49^, we first simulated EVL wound closure behavior in wild-type and keratin-deficient embryos (Fig. 6A–F). To this end, we initialized a disordered tissue patch under tension and generated a model wound by deleting several cells and adding a contractile cable along the wound perimeter (Fig. 6A–D, Supplementary Video 15; see SI section 4 for details). Wound closure in the presence of a cable with tension $\gamma=2$ was similar in both wild-type (*β* = 0.005) and keratin-deficient (*β* = 0) tissues, matching our experimental observations (Fig. 4G-G’).

Interestingly, our simulations also predicted that junctional tension, ${t}_{i}=\,{\varGamma }_{i}({P}_{i}-{P}_{i,0})$, is both higher in magnitude and propagates further into the tissue surrounding the wound in wild-type compared to keratin-deficient conditions (Fig. 6B, D). These predictions are consistent with our experimental findings that tissue flows - initially directed away from the wound during tissue recoil (prior to formation of the supracellular actin cable at the wound perimeter) and subsequently toward the wound during closure - extend further into the tissue in wild-type than in keratin deficient embryos (Fig. 4E–G).

To test whether our model reproduces these flow patterns, we analyzed simulated cell flows during both recoil and closure under wild-type and keratin-deficient conditions. To examine recoil, we simulated wounding in the absence of a tension cable at the wound perimeter (Supplementary video 17), and quantified the early response. This response was both larger in magnitude and propagated further into the tissue in wild-type than in keratin-deficient simulations (Fig. 6F). During wound closure, simulated in the presence of a supracellular tension cable, cell displacements were again consistently larger at distances away from the wound in the wild-type case, although they were reduced in the immediate vicinity of the wound (Fig. 6G–I).

Overall, the close agreement between experimental measurements and model predictions supports the hypothesis that keratin enhances long-range tension propagation within the EVL tissue. Notably, the experimentally observed differences in force percolation between wild-type and keratin-deficient EVL tissue were substantially larger than those predicted by the model. This discrepancy likely arises because keratin-deficient embryos are mechanically softer than predicted by the parameters that we fitted from control embryos with low keratin expression during the initial phase of the aspiration experiments (Fig. 5C, Supplementary Fig. 7A–D and SI section 2).

To test whether we can also simulate the behavior observed for EVL spreading during epiboly in wild-type and keratin-deficient embryos, we simulated epiboly by representing the EVL tissue as a disordered circular packing of *N* = 529 cells, where the outer vertices are pulled outwards (Fig. 6L, Supplementary Fig. 10A–C and Supplementary Video 16). As the actual embryo is spherical, we defined the effective height of the model tissue as $z=\frac{(\mathop{\sum }_{i}{A}_{i})}{(2\pi R)}$, which is the height of its projection on a sphere of radius ${R}$ = 350 µm, and we defined the tissue’s velocity ${v}_{z}$ as a derivative of this height with respect to time. To mimic the pulling of the EVL margin by actomyosin contraction and flow within the YSL, we applied an outward force ${F}_{{YSL}}$ on the edge of the simulated tissue with an amplitude which increases linearly with time, following the experimentally determined pulling force evolution within the YSL^10^. We then measured the mean keratin expression $K$ and edge velocity ${v}_{z}$ as a function of time and pulling forces (Fig. 6M–O). By ramping the pulling force from $0$ at the beginning to $0.57$ ${{\mathrm{\mu N}}}$ (a value equivalent to the pulling force used in the aspiration experiments) at the end of the simulation, we obtained keratin expression values and tissue edge velocities matching our experimental observations (Figs. 1C, 3D and 6M). We also found, similar to our experimental observations (Fig. 6K), that keratin expression increased to heterogeneous levels in individual EVL cells in our simulations (Fig. 6J, Supplementary Figs. 11 and 12), suggesting that this heterogeneous keratin expression is mechanically regulated. In this simple model, the level of heterogeneity (measured by the width of keratin expression distributions) does not quantitatively match our experimental observations (Fig. 6K). However, as shown in SI section 9 (Supplementary Fig. S11), the level of keratin heterogeneity strongly depends on ${s}_{0},$ the vertex model shape parameter, being higher for the rigid disordered systems with states of self-stress^46^ encountered when ${s}_{0}\ll 3.81$. They are also enhanced by other sources of cell-intrinsic heterogeneity e.g., in mechanosensitivity, captured by parameter α, possibly due to differences in the actin cytoskeleton and the stress it generates (Supplementary Fig. 12) between individual EVL cells. Regardless of the exact mechanisms determining the level of keratin heterogeneity between EVL cells, our model supports the general conclusion that the origin of the heterogeneous keratin expression is mechanical.

To determine whether our model can also account for the experimentally observed changes in EVL dynamics when pulling forces within the YSL were either increased or decreased, we analyzed the response of keratin expression and tissue edge velocity to variations in pulling forces (${F}_{{YSL}}$) in our simulations (Fig. 6M–O). Consistent with experimental observations, mean keratin expression increased when ${F}_{{YSL}}$ was upregulated and decreased when ${F}_{{YSL}}$ was downregulated (Fig. 6M). Moreover, edge velocity in our simulations did not react strongly to changes in ${F}_{{YSL}}$ in the wild-type (*β* = 0.005) condition, in stark contrast to the keratin deficient (*β* = 0) scenario where it increases linearly (Fig. 6O). The origin of this rheostat-like behavior is that the fixed point of the nonlinear keratin-strain rate dynamics is nearly independent of the applied stress, thus leading to similar tissue expansion rates at increasing applied forces, due to increasing keratin values stiffening the tissue (see SI section 5). However, these predictions did not align with our experimental findings at later stages of gastrulation, where both increasing and decreasing ${F}_{{YSL}}$ - by modulating actomyosin contractility within the YSL - slowed EVL epiboly movements (Supplementary Fig. 2D). This suggests that changes in keratin expression, and consequently EVL tissue viscosity, cannot fully compensate for alterations in ${F}_{{YSL}}$ throughout gastrulation. A similar discrepancy between model predictions and experimental results emerged when comparing tissue edge velocity in wild-type and keratin-deficient embryos. While our simulations predicted a significant increase in edge velocity in keratin-deficient embryos compared to wild-type (Fig. 6N–O) - as expected for a less viscous and more deformable EVL - experimental data showed a decrease in edge velocity in keratin-deficient embryos (Fig. 3D).

**Fig. 7:**
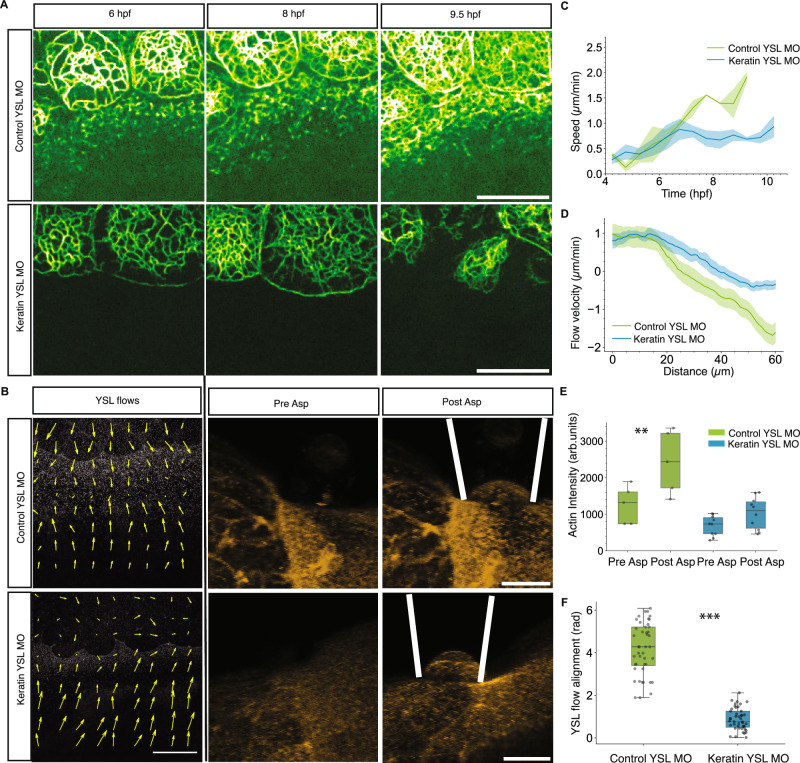
Actin flow alignment within the YSL is dependent on keratin expression. **A** Maximum intensity projections of the keratin network at the EVL-YSL boundary of Tg*(krt18:Krt18GFP)* embryos at shield (left column), 75% epiboly (middle column) and 95% epiboly (right column) injected with 2 ng control MO (top row) or 1 ng *keratin 4* plus 1 ng *keratin 8* MO (bottom row) into the YSL at sphere stage (3.3 hpf). Scale bar: 25 µm. **B** Left column: Representative maximum intensity projections of actin in Tg(*actb2:Utrophin-mcherry,krt18:Keratin18GFP*) at 6 hpf injected with 2 ng control MO (top panel) or 1 ng *keratin4* MO plus 1ng *keratin8* MO (bottom panel). Images are overlaid with quiver plots of retrograde actin flows within the YSL. Scale bar: 50 µm. Middle and right columns: Maximum intensity projections of the actin network at the EVL-YSL boundary of Tg*(actb2:Utrophinmcherry,krt18:KeratinGFP)* embryos at 6 hpf injected with 2 ng control MO (middle column) or 1 ng *keratin 4* plus 1 ng *keratin 8* MO (right column) into the YSL at sphere stage (3.3 hpf) before (Pre Asp, top row) and after (Post Asp, bottom row) micropipette aspiration. Scale bar: 25 µm. **C** Plot of EVL epiboly movement speed as a function of time (hpf) during epiboly in Tg*(actb2: Utrophin-mcherry, krt18:Krt18GFP)* embryos injected with 2 ng control MO (green) (*N* = 4, *n* = 8 embryos) or 1 ng *keratin 4* plus 1 ng *keratin 8* MO (blue) (*N* = 4, *n* = 7 embryos) into the YSL at sphere stage (3.3 hpf). Data are presented as mean line ± standard error of the mean (SEM) ribbon. **D** Plot of retrograde actin flow velocity in the YSL as a function of distance from the EVL-YSL boundary in Tg*(actb2: Utrophin-mcherry, krt18:Krt18GFP)* embryos at shield stage (6 hpf) injected with 2 ng control MO (green)(*N* = 3, *n* = 6 embryos) or 1 ng *keratin 4* plus 1 ng *keratin 8* MO (blue) (*N* = 3, *n* = 6 embryos) into the YSL at sphere stage (3.3 hpf). Data are presented as mean line ± SEM ribbon. **E** Box plot of actin intensity in a fixed region of interest (ROI) within the YSL close to the point of micropipette aspiration before (Pre Asp) and after (Post Asp) aspiration in Tg*(actb2: Utrophin-mcherry, krt18:Krt18GFP)* embryos at 3.3 hpf injected with 2 ng control MO (green boxes, *N* = 3 *n* = 10 embryos) or 1 ng *keratin 4* plus 1 ng *keratin 8* MO (blue boxes *N* = 3 *n* = 13 embryos) into the YSL at sphere stage (3.3 hpf). *N* = 4, *n* = 13 embryos. Box plots show the distribution with median (center line), interquartile range (box), and whiskers extending to values within 1.5 × IQR. (*p* values: ***<0.001, **<0.01; paired *t* test *p* (control) = 0.002867,  *p* (keratin MO) = 0.04717). **F** Box plot of actin flow alignment within the YSL at 6 hpf in Tg*(actb2: Utrophin-mcherry, krt18:Krt18GFP)* embryos injected with 2 ng control MO (green box, *N* = 3 *n* = 6 emrbyos) or 1 ng *keratin 4* plus 1 ng *keratin 8* MO (blue box, *N* = 3 *n* = 6 embryos) into the YSL at sphere stage (3.3 hpf). Box plots show the distribution with median (center line), interquartile range (box), and whiskers extending to values within 1.5 × IQR. (p values: ***<0.001; Mann Whitney test *p* = 8.3e-16). Source data are provided as a Source Data file.

Interestingly, in our simulations, the reduction in EVL tissue edge velocity observed in keratin-deficient embryos could only be explained by a decrease in the pulling force ${F}_{{YSL}}$ (Fig. 6O and supplementary video 18). This led us to speculate that keratins may have additional functions within the YSL in regulating ${F}_{{YSL}}$. Supporting this hypothesis, both our findings and previous studies indicate that keratins are also expressed within the YSL (Fig. 7A and Supplementary Fig. 5A) and play a role in actomyosin network organization and mechanosensation^50,51^. To test this possibility, we analyzed dynamic actomyosin reorganization within the YSL in the presence and absence of keratins. In wild-type embryos, retrograde flows of actin and myosin within the YSL led to the formation of a contractile actomyosin band, previously shown to generate the pulling forces that drive EVL spreading^10^. However, in embryos where keratin 4 and 8 were specifically knocked down within the YSL - achieved by injecting *keratin 4/8* morpholinos directly into the forming YSL at the 512-cell stage - these actomyosin flows were severely diminished and less aligned (Fig. 7A, D, F).

To investigate the functional interaction between keratins and the actomyosin network within the YSL, we examined whether tension generated at the EVL-YSL boundary - and propagating into both layers - not only promotes keratin network formation within the EVL, but also enhances actomyosin contraction and flow within the YSL. To test this possibility, we analyzed the coordination of actin and keratin network dynamics within the YSL during EVL epiboly movements.

Using PIV analysis, we observed both actin and keratin retrograde flows within the YSL, with keratin flows being more spatially confined to the EVL-YSL boundary where the keratin network was predominantly localized (Supplementary Fig. 5B–C’). Moreover, actin flows further away from the EVL-YSL boundary were strongly reduced when reaching the region of keratin localization close to the boundary, suggesting that actomyosin contraction-driven flows compress the keratin network by physically interacting with it as previously reported^40^.

To assess whether this interaction impacts force transmission within the actin network - potentially by keratins providing a rigid scaffold that enhances actin network crosslinking - we performed UV-laser ablation of the YSL actin network in embryos with or without keratin expression in the YSL. Strikingly, the actin recoil velocity and hydrodynamic length following laser cuts was significantly reduced in *keratin 4/8* morphants compared to controls (Supplementary Fig. 5D–E), indicating decreased force transmission within the actin network in the absence of keratin.

Given this keratin-dependent effect on actin network rheology, and previous reports linking keratins to actomyosin mechanosensation^51,52^, we next asked whether keratin within the YSL might be required for the YSL actin network responding to tension generated at the EVL-YSL boundary. To address this, we performed micropipette aspiration of the YSL and monitored mechanosensitive changes in the actomyosin network in both wild-type and keratin deficient embryos. In wild-type embryos, pulling on the YSL induced a local increase in actin intensity near the site of aspiration (Fig. 7B, E), suggesting that YSL actomyosin contraction and flow is upregulated by tension generated at the EVL-YSL boundary. In contrast, embryos with specific knockdown of *k*e*ratin 4/8* in the YSL failed to show this upregulation (Fig. 7B, E), suggesting that this mechanosensitive response of the actomyosin network depends on keratin network formation within the YSL. In these embryos with YSL-specific knockdown of *keratin 4/8*, upon UV-laser ablation of the YSL actin network lead to a decreased actin recovery over the ablated actin cortex (Supplementary Fig. 5F).

Collectively, these findings suggest that keratins are required for the generation of tension within the YSL that drives EVL epiboly movements, likely by supporting actin network crosslinking and its mechanosensitive response to applied forces.

## Discussion

Our findings suggest that keratins function in EVL spreading during epiboly by coupling tissue contractility and tension to tissue viscosity and connectivity. During epiboly, actomyosin contraction and flow within the YSL generate the forces pulling the margin of the EVL towards the vegetal pole of the gastrula. These pulling forces not only induce stress within the EVL, but also within the YSL to which the EVL is mechanically coupled at its margin. In the EVL, build-up of stress triggers keratin network maturation, which again increases EVL tissue viscosity and promotes cell-cell junction formation and maintenance, thereby ensuring that the tissue remains intact when stress rises during epiboly. In the YSL, keratins are essential for transmitting forces within the actin network, promoting stress-dependent actin accumulation, and enabling efficient contractions required to pull the EVL over the yolk cell. By fulfilling this dual mechanosensitive role - linking EVL viscosity to YSL contractility - keratins ensure robust and efficient EVL spreading during epiboly.

Keratins have previously been shown to be stress-responsive and display important functions in mechanical tissue resilience and spreading^34,52–54^. While spreading necessitates malleability, mechanical resilience is facilitated through properties such as tensile strength. Various studies have linked keratins to both these functions in culture cells and mouse embryos^34,55^; however, it remains unclear exactly how keratins could mediate these contrasting roles in a systemic manner. Our findings suggest a mechanism by which to reconcile the functions of keratins in ensuring tissue integrity^32,53,56^ and promoting tissue spreading^31,57^. The ability of keratins to resist mechanical stress increases with the progressive transformation of keratin organization from an immature disconnected form to a dense, interconnected cellular network which finally organizes into a supracellular tissue-scale network through desmosomal junctions between cells^22^. Keratin association to the desmosomal junctions leads to stabilization and reduced turnover of desmosomal proteins^23,58,59^. To ensure that this increasing resistance against deformation of the EVL does not stall EVL spreading and epiboly movements, keratins also facilitate tension-dependent actin network accumulation within the YSL, thereby adapting mechanical pulling force production within the YSL to EVL tissue viscosity resisting its deformation.

Previous studies have suggested that keratins can interact with the actomyosin cytoskeleton^52,60^, although the biochemical basis and functional significance of this interaction during early development remain poorly understood. Keratin-actin interactions are thought to occur either directly^41,52^ or indirectly via large cytolinker proteins such as plakins and plectin^61,62^. Disruption of the actin cytoskeleton has been shown to impair keratin network organization and stability^40^ and actomyosin contractility helps to direct keratin remodeling^15^. Conversely, keratin loss can perturb actin network architecture during wound healing^55^, as well as impair actin stress fiber formation and cell polarization in response to local mechanical forces acting on C-cadherins^63^. Moreover, Plectin-mediated coupling lets actin-generated forces be borne and redistributed by keratin IFs, stabilizing cell–cell and cell–matrix adhesions under stress^64^. Our results provide direct evidence that keratin and actin networks interact functionally: keratins are required for actin mechanosensation within the YSL, and conversely, actin is necessary for keratin mechanosensation within the EVL. Whether these interactions are purely mechanical - for example, providing structural support for network assembly and remodeling - or also involve biochemical signaling pathways that regulate polymerization and architecture remains to be determined.

Notably, keratin intermediate filaments are thought to be absent in multiple insect species^7^, suggesting that tissue morphogenesis and spreading in these animals can occur in the absence of keratin function. In embryogenesis of the insect *Tribolium castaneum*, for instance, the extraembryonic serosa, a simple squamous epithelial cell layer, undergoes massive spreading during epiboly^65^. Similar to EVL epiboly movements in zebrafish, serosa spreading is mediated by forces pulling on its leading edge^7,65^. Interestingly, this pulling leads to a regionalization of the serosa tissue into a solid-like dorsal portion with little cell rearrangements and a fluid-like ventral portion consisting of cells undergoing intercalations^65^. In contrast, no such clear regionalization can be observed in the zebrafish EVL with very little cell intercalations occurring throughout the tissue except some cells at the EVL margin withdrawing from the leading edge at very late stages of EVL epiboly. This different response of the EVL and serosa tissues to pulling forces might be due to the presence and absence of keratin expression within the respective tissues, pointing to the intriguing possibility that the function of keratins for homogeneous tissue spreading has become dispensable in insects. How this function of keratins in epithelial tissues has been adapted to specific organismal settings, and why keratins became expendable in many insect species remains to be explored.

While keratins belong to the most abundant and diverse cytoskeletal components in epithelial cells, remarkably little is yet known about the mechanisms by which they function in epithelial tissues. Our findings identify a critical role of keratins in promoting tissue viscosity and contractility in response to tissue tension. This ensures robust tissue spreading by balancing tissue integrity and expansion.

## Methods

### Experimental model and subject details

Zebrafish (*Danio rerio*) were maintained in the aquatics facility at ISTA, and embryos were collected, raised at 28–31 °C and staged as previously described^7^. The following wild-type (WT) and transgenic lines were used in this study: TL and AB wild-type and Tg*(krt18:Krt18GFP)*, Tg*(actb2:Utrophin-mcherry)*, Tg*(actb2:Lifeact-GFP)*, and Tg*(acbt2:Utrophin-mcherry, krt18:Krt18GFP)* transgenic strains. All experiments were carried out according to the local regulations (Breeding 2023-0.288.351), and all procedures were approved by the Ethics Committee of ISTA regulating animal care and usage.

### qPCR

For developmental *keratin* expression profiling, total RNA was extracted from 20 embryos at 1k (3.3 hpf), sphere (4 hpf), shield (6 hpf) and bud stage (9.5 hpf) using 750 µl Trizol (Invitrogen). For determining mechanosensitivity of *keratin* expression, total RNA from 20 embryos injected with either 100 pg *CAMypt* mRNA together with 0.2% phenol red or phenol red alone (control) into the YSL at high stage (3.5 hpf) was extracted at shield (6 hpf) and bud stage (9.5 hpf). DNA-free™ DNA Removal Kit (Thermo Fisher Scientific) was used to clear the Genomic DNA following the manufacturer’s protocol. 3 µg of RNA for each sample was taken as starting material to produce cDNA. NoRT control samples were produced with the Maxima H Minus First Strand cDNA Synthesis Kit following the manufacturer’s protocol. Linear amplification for the primers was tested using a series of dilutions for the cDNA to generate a standard curve. A 1:10 cDNA dilution was then chosen for the qPCR amplifications. For normalization, GAPDH, as a housekeeping gene, was amplified^66^. The following primers for *keratin(krt)4, 5*, *8* and *18 were used:*

*krt4 forward: GCAGTCTATGAGGCTGAACTCC*

*krt4 reverse: CTCAGCCTTTGTTGAGCGGA*

*krt5 forward: ACT TCC TTC AAA ACC TTC ACC*

*krt5 reverse: CCA GAT CCT GCT CCA AAA C*

*krt8 forward: CCA CCT ACA GCA AGA AAA CC*

*krt8 reverse: AGAGATGAAGCCACTACCAC*

*krt18 forward: GTAACATCCAGCATCAGACG*

*krt18 reverse: CACAACCTTTCCATCCACC*

The qPCR runs were performed on the Bio-Rad C1000 Thermal Cycler in triplicates using the Luna qPCR master mix(NEB).

### CRISPR/Cas9 mutant generation

To generate F0 crispants/mutants, Alt-R Cirspr-Cas9 kit was adapted to use a triple guide-mediate knockout of both *keratin4* and *keratin8* genes as described^67^. For each guide RNA, 1 μl crRNA 200 μM together with 1 μl tracrRNA 200 μM were annealed in 1.5 μL Duplex buffer (IDT) by heating to 95 °C for 5 min and subsequent cooling on ice. 3 gRNAs were designed against 3 distinct exons to target the whole locus of *keratin4* and *keratin8*, each. RNPs were generated by annealing the gRNAs with Cas9 protein at 37 °C for 15 min.

For *keratin4*/*keratin8* mutants, 3 cas9 RNPs at 28.5 fmol (1000 pg) total gRNA together with 28.5 fmol (4700 pg) Cas9 protein (1 Cas9 to 1 gRNA) (IDT) against each gene were injected into one-cell stage embryos. The phenotype and survival rate were scored after each injection to test the efficacy of the knockouts after each injection. The following guide sequences were used:

*keratin8*:

#1: GCCACGATTGACACCTCCAT

#2: GATCAAGGACACCTCAGTCG

#3: TCAATCTTGGACTACTTCAG

*keratin4*:

#1: CCAGAGGGCCAAGCAAGACA

#2: GAACATGCAAGGCCTGGTTG

#3: CCACGATGGAGTCCATATCC

### Cloning of expression constructs

Total RNA was extracted from 20 WT embryos at 4 and 8 hpf after dechorionation using 750 µl Trizol (Invitrogen). The cDNA library was generated with the Superscript III reverse transcription kit following the manufacturer’s instructions. The coding region of zebrafish *keratin18* was isolated using the following primers:

forward: 5′- GGGGACAAGTTTGTACAAAAAAGCAGGCTTAATGAGTCTGAGAACAAGCTACAGCG-3′

reverse: 5′- GGGGACCACTTTGTACAAAGAAAGCTGGGTTTTAAAGTTTCCTCTCCTTGGTTTCTGTGC −3′.

The dominant negative (DN) version of *keratin18* was generated by mutating the Arginine to Cysteine at position 93 using the following primers:

forward: 5′-CATGCAGAACTTGAACGACTGTCTGGCCTCCTATCTGGAG −3′

reverse: 5′-CTCCAGATAGGAGGCCAGACAGTCGTTCAAGTTCTGCATG −3′

The template DNA was then digested using dpn1, and the cDNA fragments were cloned into a pDEST plasmid using Gateway cloning. After transformation in NEB 5-alpha *E. coli* strain in a pCS2 plasmid, the clones with the correct sequences were selected using sequencing.

For *keratin8* and *4*, the genomic fragments were isolated from cDNA libraries obtained from RNA of 8 hpf embryos as described above. The following primers were used to isolate specific DNA fragments:

*keratin8* forward: 5′- GCATGGACGAGCTGTACAAGAAGACAGAAAACACACAAGGCAGGATGAGTACG −3′

*keratin8* reverse: 5′- GCTGGTTTTCTTACTATACGTACTCATCCTGCCTTGTGTGTTTTCTGTCTTCTTG −3′

*keratin4* forward: 5′- GGCATGGACGAGCTGTACAAGCTCAAAGACACGGGGATCATGTCGACGCGCTCTATCTCT −3′

*keratin4* reverse: 5′- GTAATACGACTCACTATAGTTCTAGAGGCTTAATAGCGTTTACTGCTGACGGTGG −3′

The PCR products were then integrated to generate entry vectors via recombining with pDONR(P1-P2) (Lawson#208) and the entry clone was further recombined with pCS-N-term-mEmerald (Lawson #223) or pCS-N-term-mCherry (Lawson #362) destination vector (*krt4-mcherry, krt8-mcherrry, krt4-mEmerald, krt8-mEmerald*) or p3E mNeonGreen, pCS2-Dest (Lawson #444) for C-terminal tagging (*DNkrt18*).

### mRNA and *morpholino* injections

Constructs for obtaining *keratin4*, *8*, and DN*krt*18 mRNA were generated as described above (Cloning of expression constructs). mRNA was transcribed using the SP6 mMessage mMachine Kit (Ambion). For injections, glass capillaries (30–0020, Harvard Apparatus) were pulled using a needle puller (P-97, Sutter Instruments) and mounted on a microinjection system (PV820, World Precision Instruments). Injections at the one-cell stage were performed into the one cell embryo as previously described^65^. YSL injections were performed at high-stage (3.3 hpf) by injecting directly into the newly formed YSL through the yolk. 25 pg of *krt*4*-mcherry* mRNA were injected in one-cell stage embryos. Injecting higher mRNA amounts of single keratin isoforms (>100 pg) led to aberrant keratin network formation. *DNkrt18* injections at 150 pg lead to a marked effect on  keratin network organization  at early stages of keratin network maturation. No toxicity was seen at these concentrations and embryo survival was indistinguisable from the phenol red injected embryos. For lowering actomyosin contraction within the YSL, 100 pg *CAMypt* mRNA together with 0.2% phenol red was injected into the YSL. As controls, embryos were injected with 0.2% phenol red alone into the YSL^10,11^. For increasing actomyosin contraction within the YSL, 0.5–1 pg *CARhoA* together with 2 pg *H2A*-mCherry mRNA were injected into marginal blastomeres at the 128-cell stage as previously described^11^. Keratin intensity and density analyses were restricted to the regions within the YSL where mCherry labeled nuclei were clearly detectable. For generating clones of EVL cells with reduced contractility, 100 pg *CAMypt* mRNA together with 0.2% phenol red were injected into a single blastomere of 64–128-cell staged embryos. The clones were identified by the localization of phenol red. Keratin intensity in the clone was compared to a similar sized region of interest (ROI) within the EVL at least 2 cells away from the boundary of the clone.

### For *morpholino* (MO) injections, keratin (*krt)4, 8, and 18* MOs were designed as previously described^37^

*krt4* MO: AGACCTGGTTGACATGATGCCTGTG

*krt8* MO: GGTTTTCTTGCTGTAGGTGGACATC

*krt18* MO: TGTAGCTTCTTCTCAGACTCATGGT

1 ng of each MO together with 0.2% phenol red was injected either at the one-cell stage (for uniform knock-down) or at the high-stage (3.3 hpf) (for YSL-specific knock-down). As control, 2 ng of a human *beta globin* MO (5′ - ‘CCTCTTACCTCAGTTACAATTTATA’ − 3′, Gene Tools) was injected.

### Morpholino rescue

To rescue the *keratin8* morphant phenotype, we co-injected a modified *keratin8* mRNA construct along with the *morpholino* (MO). To reduce binding of the MO to the ATG sequence of the mRNA, the sequence of mRNA was modified such that the amino acids at position two to four (Ser, Thr, Tyr and Ser) of the coding sequence with alternate codons that code for the same amino acids with different sequences:

forward MO insensitive *krt8:* gcatggacgagctgtacaagATGAGTACGTATAGTAAGAAAACC

reverse MO insensitive *krt8*: TTCTTACTATACGTACTCATcttgtacagctcgtccatgcc

The modified mRNA was cloned into a pCS2 plasmid and verified by sequencing.

Embryos were injected at one-cell stage with 1 ng of *keratin8* MO or control MO together with 0.2% phenol red and 100 pg of *keratin8* mRNA for the rescue.

### Embryo mounting and imaging

For inverted embryo imaging, embryos were dechorionated and mounted in 0.3%–0.5% low melting point (LMP) agarose in E3 (Invitrogen) on glass bottom dishes (MatTek) and then imaged on a Nikon CSU W1 with a CFI Plan Apo VC 60x WI/NA 1.2/WD 0.28-0.31 mm/Water, CFI Plan Apo λ 40x air/NA 0.95/0.17–0.25 mm or Leica Stellaris 8 (pipette aspiration experiments). For upright imaging, molds were made using 3% agarose with wells in which the embryos were mounted in a lateral position and covered with 0.5%-0.6% low-melting point agarose. Samples were then imaged on a Leica SP5 or Leica SP8 microscope with a HC FUOTAR L 25x/0.95 W (# 15506374), WD = 2.5 mm, wide angle (41°) objective. Fixed samples were mounted in 0.5–1% LMP agarose and put into prepared agarose moulds (2%) for upright imaging. Live embryos were imaged at 28.5 °C ± 1 °C.

### Analysis of EVL spreading

To determine EVL progression throughout development, the margin of the EVL was tracked along the orthogonal section of laterally imaged embryos, allowing to accurately track EVL movement over the yolk surface. The interval time was set to be 15 min for the acquisitions. In case of shorter acquisition time intervals, the EVL progression was interpolated to gain tracking intervals of 15 min. The speed measured by this method was identical with the speed measured by Particle image velocimetry (PIV) flows described below (analysis of actomyosin flows)^71^.

### UV cell ablation and cortical actin laser cutting

A Nikon CSU W1 SoRa+NIR spinning disk microscope with a home-built UV laser ablation system was used for ablating cells and monitoring the wound healing response. Ablations were performed by a single UV-laser ablation line over at least 4–5 cells with the intensity of the UV laser set at a level causing spontaneous cell delaminations. Z-stacks of 5–10 μm were acquired at 30 s intervals. EVL cells were segmented with Cellpose^68,69^ as described below (segmentation and tracking of EVL movements). Tissue flow velocity was measured using PIV with PIVlab^71^ and the cell segmentations were used to determine the orientation of the flows. Recoil flow analysis of the wound edge directly following the ablation was completed before actin accumulated on the wound edge. Wound closure flows were measured when actin had accumulated on the wound edge and recoil movements had ceased.

For the actin network hydrodynamic length measurements within the YSL, the actin network at the apical surface of the YSL was cut using the same UV-laser setup as described above. Cuts were performed at an intensity such that the cortex was ablated without eliciting a wound healing response, as evidenced by the formation of an actomyosin cable closing the wound site. ROI spanning 40 µm on each side of the cut were used to calculate the flow fields using PIV tacking. The analysis of actin recovery in the cut was performed using a manual line ROI over the cut in Fiji and plotted in Python using matplotlib and pandas. The hydrodynamic length of these flows was determined by averaging the flow fields on each side of the cut and fitting the decay of the averaged flows to an exponential decay using scipy.

### Pipette aspirations

Pipette aspirations were performed to measure the viscosity of the EVL as previously described. In short, pipette aspirations were carried out on embryos mounted in 3% methylcellulose in E3 on an inverted Leica SP5 or a Leica Stellaris 5 confocal microscope equipped with the micropipette aspiration system. For creep recovery experiments to measure viscosity, fire-polished and with heat-inactivated FBS passivated micropipettes (Biomedical Instruments) with an inner diameter of 60 μm, 30° bent positioned by micromanipulators (TransferMan Nk2, Eppendorf), and with a blunt end were placed on the EVL around 3–4 cells away from the EVL margin. A negative pressure was applied with an increment of 10 Pa using a Microfluidic Flow Control System Pump (Fluiwell, Fluigent) and Dikeria micromanipulation software^70^. Images were acquired every second. A negative pressure of 200 mbar was applied until the aspirated tissue flowed in the pipette with constant velocity, followed by pressure release. The length of the aspirated tissue was measured using a custom-made Fiji macro and plotted over time to calculate the aspiration and retraction speed. The viscosity was calculated as previously described^42,43^. Pipette aspirations were also used to study the mechanosensitive response of the keratin and actin cytoskeleton within the EVL (keratin and actin) and the YSL (actin). To this end, the EVL of Tg*(acbt2:Utrophin-mcherry,krt18:Krt18GFP)* or Tg*(krt18:Krt18GFP)* embryos was aspirated under constant pressure, and the mean intensities of keratin and/or actin at the midplane of the pipette and a region of 30 µm around the aspiration site were recorded. For determining keratin mechanosensitivity within the EVL when the actin cortex is disassembled, Tg*(acbt2:Utrophin-mcherry,krt18:Krt18GFP)* embryos were exposed to 25 nM Cytochalasin D 10 min before the start of aspiration. For the YSL aspirations, a z-stack of 50 µm was taken and the actin intensity in Tg*(acbt2:Utrophin-mcherry,krt18:Krt18GFP)* embryos was measured on the YSL surface in intervals of 30 s.

### Analysis of YSL actomyosin flows

To measure actin flow velocities within the YSL, Tg*(actb2:Utrophin-mcherry)* or Tg*(actb2: Lifeact-GFP)* embryos were mounted on a Nikon CSU W1 spinning disc microscope in 0.5% LMP agarose and imaged every 30 s. A 500 × 500-pixel ROI within the YSL was selected with maximum intensity projections centered along the AV axis close to the margin of the EVL. The respective ROI was then used for PIV analysis using PIVlab^71^ and post-processed by a custom-made Python script. Flow velocities represented in the kymographs were averaged over three consecutive time points. Alignment of these flows was analyzed by measuring the angular mean of the flow vectors in the ROI as described above.

### Segmentation and tracking of EVL cells

To visualize EVL cells, Tg*(actb2:Utrophin-mcherry)* or Tg*(acbt2:Utrophin-mcherry, krt18:Krt18GFP)* transgenic embryos were used to mark apical cell junctions. Maximum intensity projections of Z-stacks covering approximately 150 µm depth (for movies acquired with a SP8 confocal microscope) or 30 µm depth (for movies acquired on a Nikon spinning disc microscope) were generated for subsequent analysis. Segmentation of the EVL cells was performed using a “human in the loop” pipeline using the Cellpose segmentation software. Initial segmentations were generated using models trained on manually annotated datasets. These models were then employed to make further segmentations. Post-processing involved manually correcting any segmentation errors and using these corrections to further refine the segmentation models. Heightmaps were generated with the Local Z-projector plug-in in Fiji in order to correct for the curvature of the embryo. Deproj functions were used to integrate the segmentations and the heightmaps to measure EVL cell area and alignment. Data were processed and plotted using custom Python scripts.

### Keratin network segmentation

For analyzing the keratin cytoskeleton in EVL cells, Tg*(krt18:Krt18GFP)* embryos were imaged at 63× magnification as described above (embryo mounting and imaging). As most of the keratin network within EVL cells formed a single sheet at the apical surface, Z-projections of the keratin network over the entire EVL cell were used. First, a Gaussian filter and denoising were applied to obtain the filamentous skeleton. Next, the images were thresholded using an adaptive median threshold and the area fraction covered by the network as an estimate of network density was determined using Fiji. For junctional keratin accumulation, the cell margins were outlined using EVL segmentations as described above (segmentation and tracking of EVL cells) with a thickness of about three pixels to obtain a skeleton junction image. The skeleton junction image was then used to segment the junctional pool of keratin in each cell and the intensity of the junctional pool was measured in the segmented image. The total intensity of keratin per cell was measured using the whole apical segmented area inside the junctional segmentations.

### Fluorescence in situ hybridization

WT or Tg*(krt18:Krt18GFP)* embryos at 9 hpf were fixed in 2% paraformaldehyde at RT for 1h, followed by stepwise fixation with and subsequent storage in methanol at −20 °C. After overnight fixation in methanol the embryos were stepwise rehydrated to PBST (phosphate buffered saline, 0.1% Tween-20 pH 7.3) followed by incubation in hybridization buffer (10% Dextran sulfate, Formamide 10%, tRNA 1 mg/ml, SSC 2x, BSA 0.02%, Vanadyl-ribonucleoside complex (NEB S1402S) 2 mM) at 30 °C. Stellaris RNA FISH probes for *keratin8* mRNA were designed from zebrafish mRNA sequences using LGC Biosearch Technologies’ Stellaris® RNA FISH Probe Designer and 30 probes per probe-set were ordered. The embryos were incubated in this probe-set overnight at 30 °C. The hybridized embryos were washed with wash-buffer 3 times before mounting in gold mounting media and imaged using an inverted Leica SP5 or a Leica Stellaris 5 confocal microscope as described above.

### Statistics and reproducibility

The statistical analyses were performed using scipy stats package in Python. The number of transplants or embryos (*n*) and experimental replicates (*N*) analyzed are indicated in the figure legends. At least three independent experiments were performed in all cases whose data is provided in the Source Data file. Error bars are indicated as ribbons, whiskers or as applicable and described in each figure legend. Sample sizes were not determined using statistical tests. The statistical tests performed are collected in a Jupyter notebook available on github. Each legend describes the statistical tests performed along with the associated *p* values. To compare more than two groups, ANNOVA was performed. To increase statistical power, a correction for multiple comparisons was used. To compare two groups paried and unpaired *t*-test and Mann-Whitney test were used.

### Reporting summary

Further information on research design is available in the Nature Portfolio Reporting Summary linked to this article.

## Supplementary information

Supplementary information

Description of Additional Supplementary Files

Supplementary Movie 1: Keratin expression within the EVL during epiboly

Supplementary Movie 2: Keratin expression within the EVL in embryos with reduced YSL pulling force

Supplementary Movie 3: Keratin network maturation in EVL cells of embryos with reduced YSL pulling force

Supplementary Movie 4: Keratin expression within the EVL of embryos with enhanced YSL pulling force

Supplementary Movie 5: Keratin network maturation in EVL cells of embryos with enhanced YSL pulling force

Supplementary Movie 6: Keratin expression within the EVL of embryos with reduced keratin type II expression

Supplementary Movie 7: Keratin network maturation in EVL cells of embryos with reduced keratin type II expression

Supplementary Movie 8: Keratin expression within the EVL of embryo with reduced keratin8 expression and rescue with modified mRNA

Supplementary Movie 9: Changes in keratin expression upon EVL aspiration

Supplementary Movie 10: Changes in keratin expression during EVL wound closure after cell ablation

Supplementary Movie 11: Wound closure in control and keratin-deficient embryos

Supplementary Movie 12: E-cadherin expression in control and keratin-deficient embryos

Supplementary Movie 13: Occludin-b expression in control and keratin-deficient embryos

Supplementary Movie 14: Jup-a expression in control and keratin-deficient embryos

Supplementary Movie 15: Simulated EVL wound closure in model with and without keratin feedback onto mechanics

Supplementary Movie 16: Simulated EVL tissue undergoing epiboly

Supplementary Movie 17: Simulated recoil of EVL tissue upon wound formation

Supplementary Movie 18: Effect of normal and reduced YSL pulling force on control and keratin-deficient simulated EVL tissues

Reporting Summary

Transparent Peer Review file

## Source data

Source data

## Data Availability

The authors declare that the minimum dataset that is necessary to interpret, verify, and extend the research in this article is included in the supplementary information, the source data, and the archived data repository (10.15479/AT-ISTA-21137). This is also available on GitHub at https://github.com/Suyash-Naik/2026-Keratinepithlialspreadingcoordinate-Data. Source data are provided with this paper.

## Code availability

The framework to develop the vertex models used in this paper are available online on GitHub and archived in the source data provided. Custom scripts used for analysis of imaging and simulation data are provided along with data files for all panels in the source data for this manuscript on GitHub and in data repo (10.15479/AT-ISTA-21137). Framework for the vertex model is available at https://github.com/yketta/cells. Code for analysis is available on GitHub https://github.com/Suyash-Naik/2026-Keratinepithlialspreadingcoordinate-Data.
