# Supplementary material for: Keratins coordinate tissue spreading by balancing spreading forces with tissue material properties
Source: Nat Commun. 2026 May 16;17:6499. doi: 10.1038/s41467-026-72366-z (PMC13377027; doi:10.1038/s41467-026-72366-z)
Supplement: Supplementary file 1 — Supplementary information [file 41467_2026_72366_MOESM1_ESM.pdf]

Keratins coordinate tissue spreading by balancing spreading forces with tissue material properties
Supplementary Information

1 Supplementary experimental figures

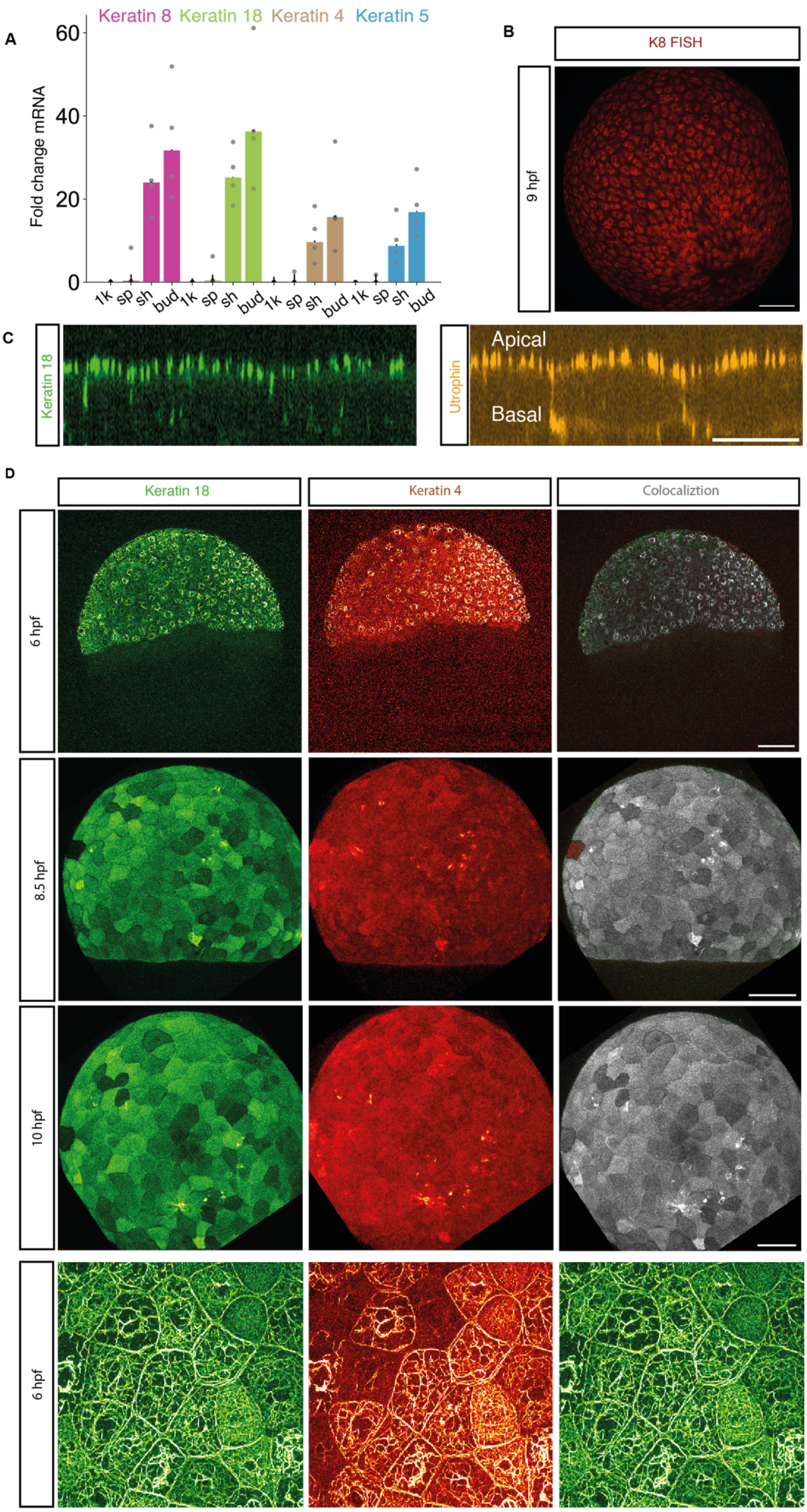
A
Keratin 8 Keratin 18 Keratin 4 Keratin 5
Fold change mRNA
60
40
20
0
1k sp sh bud 1k sp sh bud 1k sp sh bud 1k sp sh bud
B
K8 FISH
9 hpf
C
Keratin 18
Utrophin
Apical
Basal
D
Keratin 18
Keratin 4
Colocaliztion
6 hpf
8.5 hpf
10 hpf
6 hpf

Supplementary Figure 1: **Keratin expression and localization during epiboly.**

(A) Bar plot with scatter of fold change of *krt 18*, *krt 8*, *krt 4*, and *krt 5* expression in embryos at 1K (3.3 hpf), 30% epiboly (4.5 hpf), shield (6 hpf) and bud (9 hpf) stages measured by qPCR. (N = 4, n = 4 samples each with ¿40 embryos, one way ANNOVA pvalues: 0.000021 *krt 18*, 0. 000118 *krt 4*, 0.000007 *krt 5*, 0.000027 *krt 8*, 8.953314e-20 stages all).

(B) Maximum intensity projection images of *krt8* mRNA fluorescence via in situ hybridization in WT embryos at 9 hpf. Scale bar: 25 µm.

(C) Z-plane reslice image of a cross-section view of the EVL in Tg(*actb2: Utrophin-mcherry, krt18:Krt18GFP*) embryos at shield stage (6 hpf). Scale bar: 25 µm.

(D) Maximum intensity projection images of keratin network in Tg(*krt18: Krt18GFP*) embryos at shield (6 hpf), 75% epiboly (8.5 hpf) and bud (10 hpf) stages injected of 50 pg *krt4-mcherry* mRNA at the one-cell stage. Left column, krt18 (green); middle column, krt 4 (red); right column, krt 4 and 18 colocalization (white; co-localization index R below threshold 0.005). Scale bar: 100 µm (top, second and third row) and 25 µm (bottom).

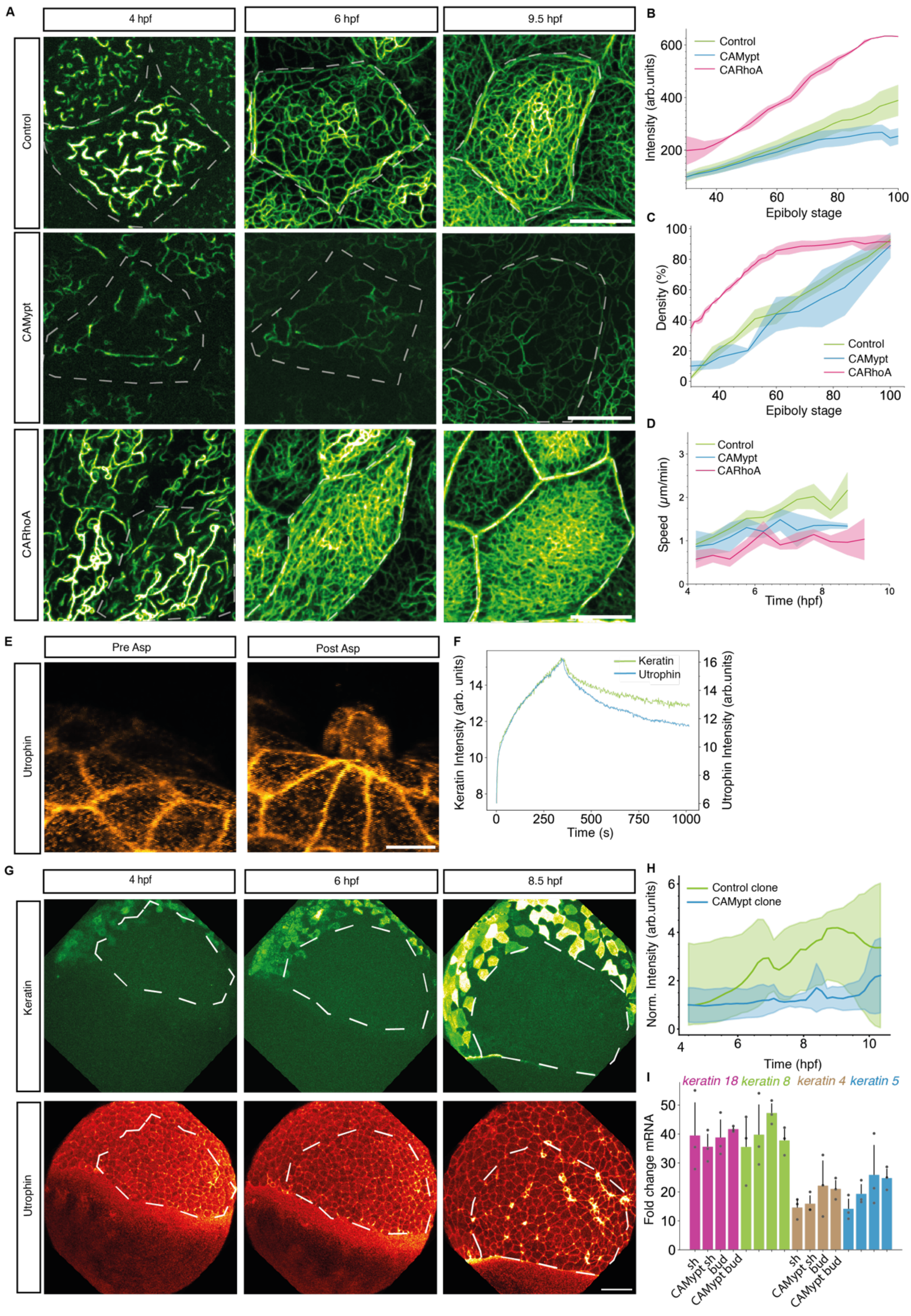
A
4 hpf
6 hpf
9.5 hpf
Control
CAMypt
CARhoA
B
Control
CAMypt
CARhoA
Intensity (arb.units)
Epiboly stage
C
Density (%)
Epiboly stage
D
Speed (μm/min)
Time (hpf)
E
Pre Asp
Post Asp
Utrophin
F
Keratin
Utrophin
Keratin Intensity (arb. units)
Utrophin Intensity (arb.units)
Time (s)
G
4 hpf
6 hpf
8.5 hpf
Keratin
Utrophin
H
Control clone
CAMypt clone
Norm. Intensity (arb.units)
Time (hpf)
I
keratin 18 keratin 8 keratin 4 keratin 5
Fold change mRNA
sh
CAMypt sh
bud
CAMypt bud

Supplementary Figure 2: **Tension-dependent regulation of keratin expression within the EVL.**

(A) Maximum intensity projection images of keratin expression in Tg(*actb2: Utrophin-mcherry, krt18:Krt18GFP*) embryos at sphere (4 hpf, left column), shield (6 hpf, right column), and 90% epiboly (9.5 hpf, right column) stages injected with 0.2% phenol red (control, top row), 100 pg *caMypt* mRNA (middle row), or 50 pg *caRhoA* mRNA (bottom row) directly into the YSL at 3.3 hpf (control, *caMypt*) or into marginal cells at 3 hpf (*caRhoA*). Scale bar: 25 µm.

(B) Plot of average keratin intensity as a function of epiboly stages in Tg(*krt18:KrtGFP*) control (green, N = 3, n = 5 embryos), *caMypt* (blue, N = 3, n = 5 embryos) and *caRhoA* mRNA injected embryos (pink, N = 3, n = 4 embryos) as described in (B). Error bars as ribbons SD of mean.

(C) Plot of average density of keratin network as a function of epiboly stages in Tg(*actb2:Utrophin-mcherry, krt18:Krt18-GFP*) control (green, N = 3, n = 6 embryos), *caMypt* (blue, N = 3, n = 4 embryos) and *caRhoA* mRNA injected embryos (pink, N = 3, n = 3 embryos) as described in (B). Error bars as ribbon SD of mean of individual cells per replicate.

(D) Plot of EVL epiboly movement speed as a function of time (hpf) during epiboly starting at sphere stage (4 hpf) until late epiboly stages (9 hpf) in Tg(*actb2: Utrophin-mcherry, krt18:Krt18GFP*) control (green, N = 3, n = 6 embryos), *caMypt* (blue, N = 3, n = 4 embryos) and *caRhoA* mRNA injected embryos (pink, N = 3, n = 3 embryos). Error bars as ribbon SD of mean of individual cells per replicate.

(E) Maximum intensity projection images of actin localization within the pipette before (right) and after (left) EVL micropipette aspiration in Tg(*actb2: Utrophin-mcherry, krt18:Krt18GFP*) embryos at 6.5 hpf. Scale bar: 15 µm.

(F) Plot of representative keratin (green; left y-axis) and actin (Utrophin; blue; right y-axis) intensities within the pipette as a function of time during EVL micropipette aspiration in Tg(*actb2: Utrophin-mcherry, krt18:Krt18GFP*) embryos at 6.5 hpf.

(G) Maximum intensity projection images of keratin (top row) and actin (bottom row) expression in Tg(*actb2: Utrophin-mcherry, krt18:Krt18GFP*) embryos at sphere (4 hpf, left column), shield (6 hpf, right column), and 90% epiboly (9.5 hpf, right column) stages injected with 100 pg *caMypt* into a single blastomere at 128 cell stage. The extent of the clone is marked with a dashed white line. Scale bar: 100 µm.

(H) Average keratin intensity measured as a function of time during development from sphere stage (4 hpf) to the end of epiboly (10.5 hpf) in Tg(*actb2: Utrophin-mcherry, krt18:Krt18GFP*) embryos stages injected with 100 pg *caMypt* into a single blastomere at 128 cell stage (CAMypt clone, blue, N = 3, n = 5 embryos) and a similar sized area in the uninjected region (control clone,green, N=3, n = 5 embryos) of the EVL.

(I) Bar plot with scatter of fold change of *keratin 18*, *keratin 8*, *keratin 4*, and *keratin 5* expression at shield (6 hpf) and bud (9 hpf) stages in embryos injected with 0.2% phenol red (control, sh and bud, N=3 n = 36 embryos) or with 100 pg CAMypt mRNA (CAMypt sh and CAMypt bud, N = 3 n= 36 embryos) into the YSL at high stage (3.5 hpf) measured by qPCR. (Three-way ANNOVA not significant pvalues: C(treatment)= ns at 9.871e-01, *krt 18* 0.638, *krt 4* 1.00 , *krt 5* 1.00, *krt 8* 0.922827).

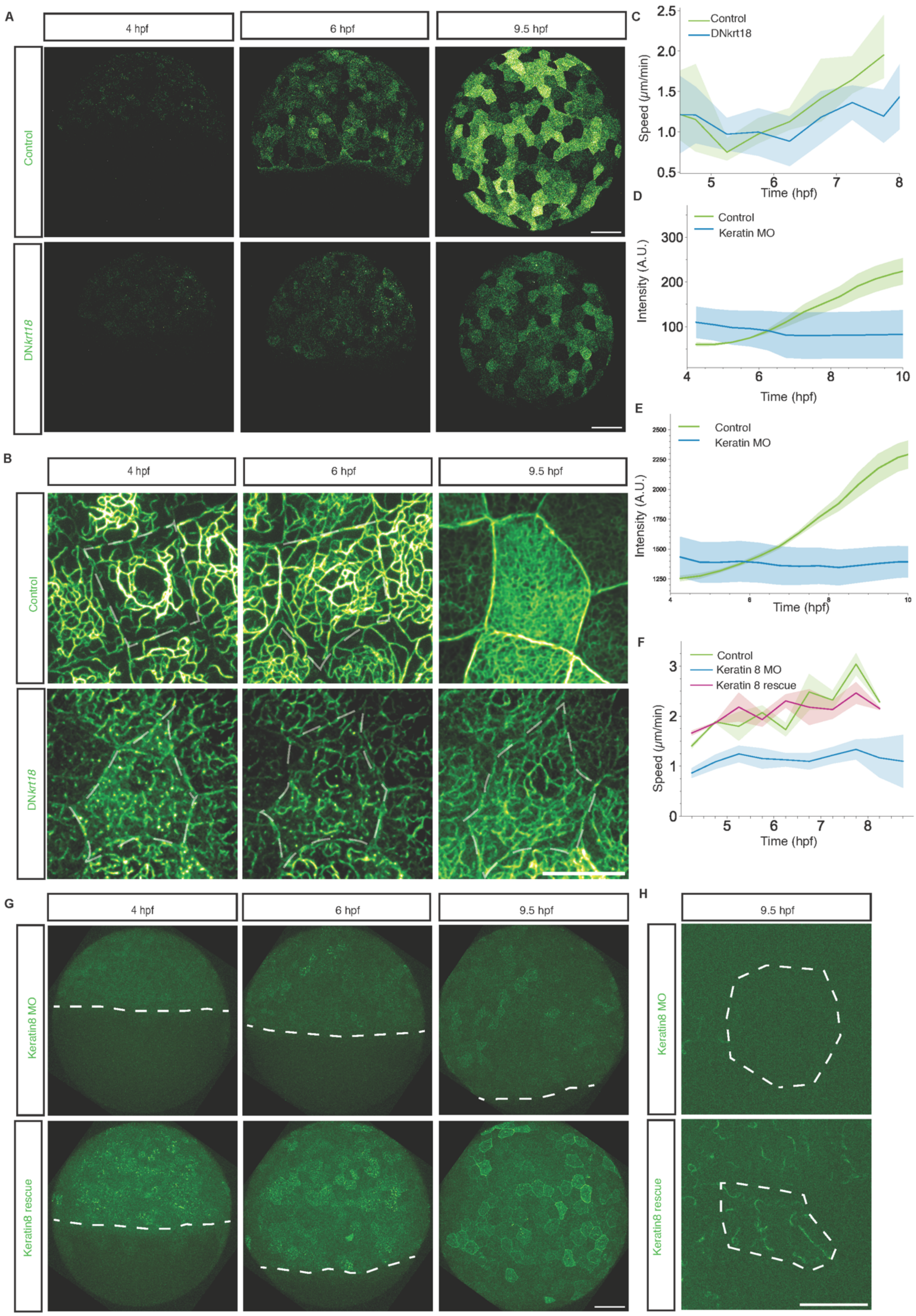
A
4 hpf
6 hpf
9.5 hpf
Control
DNkrt18
B
4 hpf
6 hpf
9.5 hpf
Control
DNkrt18
C
Control
DNkrt18
Speed (µm/min)
Time (hpf)
D
Control
Keratin MO
Intensity (A.U.)
Time (hpf)
E
Control
Keratin MO
Intensity (A.U.)
Time (hpf)
F
Control
Keratin 8 MO
Keratin 8 rescue
Speed (µm/min)
Time (hpf)
G
4 hpf
6 hpf
9.5 hpf
Keratin8 MO
Keratin8 rescue
H
9.5 hpf
Keratin8 MO
Keratin8 rescue

Supplementary Figure 3: **Effect of dominant negative keratin 18 expression on keratin network formation and EVL epiboly and keratin 4/8 morpholinos on occludin and plakoglobin expression within the EVL; rescue of the keratin 8 morphant phenotype.**

(A) Maximum intensity projection images of keratin expression in Tg(*actb2: Utrophin-mcherry, krt18:Krt18GFP*) at sphere (4 hpf, left column), shield (6phf, middle column) and bud (10 hpf, right column) stages injected with 0.2% phenol red (control, top row) or 150 pg *dnkrt18* mRNA (bottom row) at one-cell stage. Scale bar: 100 µm.

(B) Maximum intensity projection images of keratin expression in Tg(*actb2: Utrophin-mcherry, krt18:Krt18GFP*) at sphere (4 hpf, left column), shield (6phf, middle column) and bud (10 hpf, right column) stages injected with 0.2% phenol red (control, top row) or 150 pg *dnkrt18* mRNA (bottom row) at one-cell stage. Scale bar: 25 µm.

(C) Plot of EVL epiboly movement speed as a function of time (hpf) during epiboly starting at sphere (4 hpf) until late epiboly (9 hpf) stages in Tg(*actb2: Utrophin-mcherry, krt18:Krt18GFP*) embryos injected with 0.2% phenol red (control, green) or 150 pg *dnkrt18* mRNA (blue) at one-cell stage. (N = 2, n = 6 embryos).

(D) Plot of average Occludin-b intensity in as function of time in control MO (green, N = 3, n = 7 embryos) and 1 ng *keratin 4* plus 1 ng *keratin 8* MO (blue, N = 3, n = 8 embryos) injected Tg(oclnb-GFP) embryos. Error bars as ribbons SD of mean.

(E) Plot of average junctional plakoglobin A intensity as function of time in control MO (green, N = 2, n = 6 embryos) and 1 ng *keratin 4* plus 1 ng *keratin 8* MO (blue, N = 2, n = 5 embryos) injected Tg(*her4.1:jupa-EGFP*) embryos. Error bars as ribbons SD of mean.

(F) Plot of EVL epiboly movement speed as a function of time (hpf) during epiboly starting at sphere stage (4 hpf) until late epiboly stages (9 hpf) in Tg(*actb2: Utrophin-mcherry, krt18:Krt18GFP*) embryos injected at the one-cell stage with 1 ng control MO and 100pg *keratin 8* mRNA (green, N = 3, n = 3 embryos), 1 ng *keratin 8* MO (blue, N = 3, n = 3 embryos), or 1 ng *keratin 8* MO and 100pg *keratin 8* mRNA (pink, N = 3, n = 4 embryos). Error bars as SD of the mean of replicates.

(G) Maximum intensity projection images of keratin expression in Tg(*actb2: Utrophin-mcherry, krt18:Krt18GFP*) embryos at sphere (4 hpf, left column), shield (6 hpf, middle column) and late epiboly stages (9.5 hpf) injected with 1ng *keratin 8* MO (top row) or 1ng *keratin 8* MO and 100 pg *keratin 8* mRNA (Keratin rescue, bottom row). Scale bar 100 µm.

(H) Maximum intensity projection images of keratin expression in Tg(*actb2: Utrophin-mcherry, krt18:Krt18GFP*) embryos at late epiboly stages (9.5 hpf) injected with 1ng *keratin 8* MO (top row) or 1ng *keratin 8* MO and 100 pg *keratin 8* mRNA (Keratin rescue, bottom row). Scale bar 25 µm.

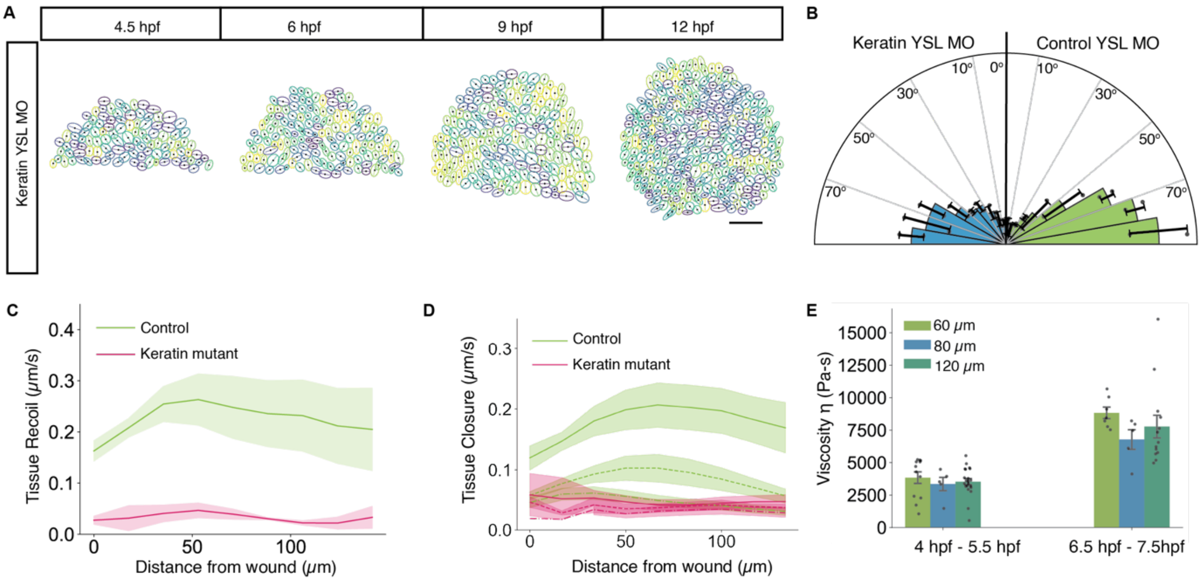

Supplementary Figure 4: **EVL cell alignment and flow along the axis of tissue spreading is independent of keratin expression within the YSL.**

(A) Exemplary plots of EVL cell orientations with ellipses representing shape descriptors (long and short axis) of individual EVL cells with the line in the middle marking the orientation of the long axis at consecutive stages during epiboly (4.5, 6, 9, 12 hpf) in Tg(*actb2:Utrophin-mcherry, krt18:Krt18GFP*) embryos injected with 1 ng *keratin 4* plus 1 ng *keratin 8* MO at high stage (3.3 hpf) directly into the YSL to specifically interfere with YSL keratin network formation. Each cell is colour-coded according to the orientation of the axis (hsv) as shown in the colour bar at the bottom (Green: AV axis orientation, blue: dorsoventral/DV orientation). Scale bar: 100 µm.

(B) Rose plot of EVL cell orientations in Tg(*actb2:Utrophinmcherry, krt18:Krt18GFP*) embryos at 6 hpf, injected at high stage (3.3 hpf) with 2 ng control MO (B; N = 4, n = 93 cells) or 1 ng *keratin 4* plus 1 ng *keratin 8* MO (C; N = 4, n = 84 cells) directly into the YSL.

(C) Average EVL tissue recoil velocity directly following EVL cell ablation plotted as a function of distance from the wound center (0 µm) immediately after cell ablation in Tg(*actb2:Utrophinmcherry*) embryos injected with 2 ng control MO (green, N = 4, n = 23 embryos) or TraCr *keratin 4* and *keratin 8* gRNA (*keratin 4/8* crispant F0; pink, N = 2, n = 6 embryos).

(D) Average EVL tissue flow during wound closure after EVL cell ablation plotted as a function of distance from the wound center (0 µm) in Tg(*actb2:Utrophinmcherry*) embryos injected with 2 ng control MO (green, N = 4, n = 23 embryos) or TraCr *keratin 4* and *keratin 8* gRNA (*keratin 4/8* crispant F0; pink, N = 2, n = 6 embryos) at successive timepoints after the ablation (20 secs, solid; 60 secs, dotted; 120 secs, dotted line with points).

(E) Bar plots with scatter of tissue viscosity measured at the EVL margin using micropipette aspiration with pipette ID 60 µm (N=5, n=33 embryos), 80 µm (N = 2, n = 8 embryos) and 120 µm (N = 2, n = 12 embryos) at 4-6 hpf and 6-7.5 hpf in Tg(*actb2: Utrophin-mcherry, krt18:Krt18GFP*) embryos. (One-way ANNOVA ns pvalues early = 0.712378 and late = 0.388632).

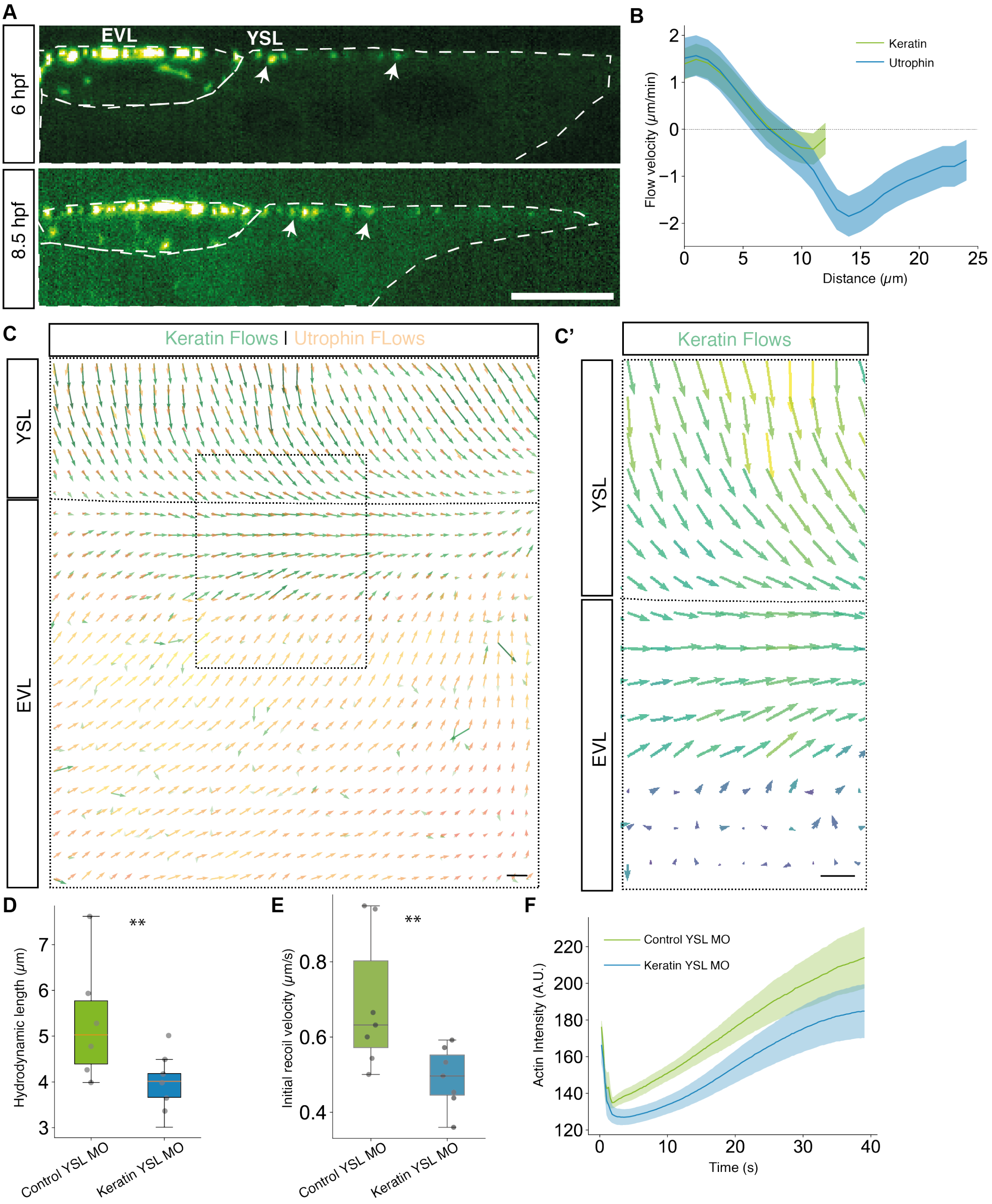
A
6 hpf
8.5 hpf
EVL
YSL
B
Keratin
Utrophin
Flow velocity (μm/min)
Distance (μm)
C
Keratin Flows | Utrophin FLows
YSL
EVL
C'
Keratin Flows
D
Hydrodynamic length (μm)
**
Control YSL MO
Keratin YSL MO
E
Initial recoil velocity (μm/s)
F
Control YSL MO
Keratin YSL MO
Actin Intensity (A.U.)
Time (s)

Supplementary Figure 5: **Actin-keratin flows within the YSL.**

(A) Z-plane reslice image of a cross-sectional view of the YSL at the EVL margin in Tg(*actb2:Utrophinmcherry, krt18:Krt18GFP*) embryos. EVL and YSL are outlined by a white dotted line, and keratin filaments within the YSL are marked by white arrows. Scale bar: 15 µm.

(B) Plot of average flow velocity of keratin (green) and actin (orange) within the YSL in Tg(*actb2:Utrophinmcherry, krt18:Krt18GFP*) embryos measured using PIV as a function of distance from the EVL-YSL boundary.

(C) Plot of a representative particle image velocimetry flows of keratin filaments (virdis) and actin cortex (plasma) at the EVL-YSL boundary in Tg(*actb2:Utrophinmcherry, krt18:Krt18GFP*) embryos measured using PIV. The dotted box outlines the region of interest represented in (C') showing a high-magnification view of the keratin flow field (viridis) at the EVL-YSL boundary. Scale bar: 1 µm/min.

(D) Box plot of the hydrodynamic length of the YSL actin network measured by UV-laser cutting of the actin cortex in Tg(*actb2:Utrophinmcherry, krt18:Krt18GFP*) embryos at shield stage (6.5 hpf) injected with control MO (green, N = 4, n = 15 embryos) or 1 ng *keratin 4* plus 1 ng *keratin 8* MO (orange, N = 4, n = 17 embryos) into the YSL at high stage (** ¿ 0.05 Mann-Whitney test pvalue: 0.009696).

(E) Box plot of initial recoil velocities of the actin cortex after laser ablations in Tg(*actb2:Utrophinmcherry, krt18:Krt18GFP*) embryos injected with control MO (green, N = 4, n = 15 embryos) or 1 ng *keratin 4* plus 1 ng *keratin 8* MO (orange, N = 4, n = 17 embryos) in the YSL at high stage (**¿0.05 Mann-Whitney test pvalue: 0.01107).

(F) Plot of actin intensity recovery after UV-laser cutting as a function of time measured within a region of interest at the cut within the YSL actin cortex in Tg(*actb2:Utrophinmcherry, krt18:Krt18GFP*) embryos at shield stage (6.5 hpf) injected with control MO (green, N = 4, n = 15 embryos) or 1 ng *keratin 4* plus 1 ng *keratin 8* MO (blue, N = 4, n = 17 embryos) into the YSL at sphere stage (3.3 hpf).

2 Experimental measurements and visco-elastic Maxwell model

Pipette aspiration experiments are performed on the gastrulating tissue as follows: The tissue is initially at rest. At time $t = 0$ a constant pressure differential ($\Delta P_{\text{ext}} = 2\,\text{mbar}$) is applied within the pipette (diameter $d = 60\,\mu\text{m}$), leading to a constant pulling force $F^0_{\text{ext}} = (\pi/4)\,\Delta P_{\text{ext}}\,d^2 \approx 0.57\,\mu\text{N}$, causing the tissue to move upwards. Then at a later time, the pressure differential is removed, *i.e.* $\Delta P_{\text{ext}} = 0$, causing the tissue to retract and move downwards in the second phase.

Supplementary Figure 6A-B shows the height of the aspirated tissue and the corresponding keratin expression for tissues at different times post fertilisation. Tissues show viscous behaviour at long times, *i.e.* deformation at constant velocity.

During our pipette experiment approximately 5 cells are aspirated into the pipette, and move with a typical velocity in the 0.1-1 $\mu\text{m}\,\text{s}^{-1}$ range. The standard model of inferring tissue viscoelasticity from pipette aspiration [3, 4] involves approximately 100 cells at a typical velocity of 0.01 $\mu\text{m}\,\text{s}^{-1}$. We will therefore in contrast assume that our experiment probes the mechanical behaviour of *individual cells* rather than the whole tissue – it is consistent with junction cutting experiments which probe cell-level elasticity and report velocities in the 0.2-0.5 $\mu\text{m}\,\text{s}^{-1}$ range [1]. Epiboly however involves again $\gtrsim$ 500 cells [2] with a typical velocity in the 0.01-0.03 $\mu\text{m}\,\text{s}^{-1}$ range [1], and so we will use our results here to set the *individual cell* parameters for a vertex model of epiboly (see Section 3).

Modified Maxwell model

To account for these observations, we introduce a simple model for a stressed viscoelastic tissue, namely a Maxwell model with additional internal tension, coupled with friction on a substrate (Supplementary Figure 6D). Let ℓ be the length of the material, which we identify with the height of the aspirated tissue in the pipette experiment. We write the constitutive equations

$$\zeta\dot{\ell} = -k(\ell - \ell_0) - T\Theta(l) + F_{\text{ext}}, \qquad \triangleright \text{ overdamped harmonic oscillator} \tag{S.1a}$$

$$\dot{\ell}_0 = \frac{1}{\tau}(\ell - \ell_0), \qquad \triangleright \text{ rest length relaxation} \tag{S.1b}$$

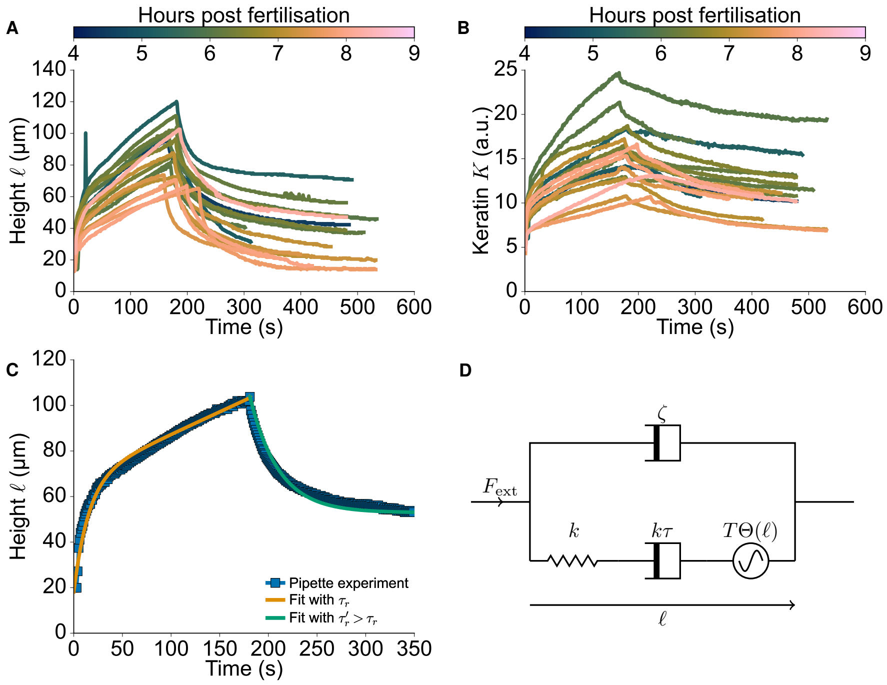

Supplementary Figure 6: (A) Height of the aspirated tissue as function of time in a normal tissue with the corresponding (B) keratin expression level in arbitrary units. (C) Fit of a sample experimental curve to the functional form equation (S.4). (D) Diagram of the Maxwell model coupled to substrate friction.

where ζ is the substrate friction coefficient, k is the elastic constant of the tissue, τ its internal relaxation time of the tissue, T is the internal tension in the tissue, Θ is the Heaviside function introduced to avoid unphysical $\ell < 0$, and F_{ext} the external applied force from the pipette. These lead to the following equation of motion for length ℓ

$$\ddot{\ell} + \frac{1}{\tau_r}\dot{\ell} = \frac{1}{\zeta\tau}\left(F_{\text{ext}} - T\Theta(l)\right) + \frac{1}{\zeta}\left(\dot{F}_{\text{ext}} - T\frac{\mathrm{d}}{\mathrm{d}t}\Theta(l)\right), \tag{S.2a}$$

$$\tau_r = \frac{\zeta\tau}{\zeta + k\tau} < \tau. \tag{S.2b}$$

Consistent with the pipette experiment, we consider F_{ext} to be a piecewise constant function. Assuming that F_{ext} and/or $\Theta(l)$ are discontinuous at time t, we compute the inital condition to solve (S.2a) at times $t' > t$ [5]

$$\ell(t^+) - \ell(t^-) = 0, \tag{S.3a}$$

$$\dot{\ell}(t^+) - \dot{\ell}(t^-) = \frac{1}{\zeta}\Big([F_{\text{ext}}(t^+) - F_{\text{ext}}(t^-)] - T[\Theta(\ell(t^+)) - \Theta(\ell(t^-))]\Big), \tag{S.3b}$$

and the form of the solution under these assumptions is

$$\ell(t) = (\tau_r(\dot{\ell}(0^+) - v_\infty) + \ell(0^+))\left[1 - \frac{\tau_r(\dot{\ell}(0^+) - v_\infty)}{\tau_r(\dot{\ell}(0^+) - v_\infty) + \ell(0^+)}e^{-t/\tau_r}\right] + v_\infty t, \tag{S.4a}$$

$$v_\infty = \frac{F_{\text{ext}} - T}{\zeta + k\tau}, \tag{S.4b}$$

provided that $\ell(t > 0) > 0$. Note that this form of the solution is identical to that of Guevorkian *et al.* [3]. However the physical assumptions at the basis of our model are different. Ref. [3] models the tissue as a three-dimensional, spherical object with a surface tension, where energy dissipation arises from viscous flow into the pipette. In contrast, we modelled the embryo as an effectively two-dimensional sheet. In this framework, the surface tension corresponds to an in-plane tension within the sheet, and dissipation occurs via friction against the pipette walls (captured by the ζ-term) as well as internal tissue rearrangements and deformations (captured by the τ-term). Additionally, we incorporated a baseline tissue tension through a constant additive term. Our approach also leads to different continuity relations (it guarantees a continuous height function contrarily to the former approach) and it allows for a straightforward parametrisation of the internal elasticity and viscous relaxation through parameters k and τ, and the substrate dissipation through parameter ζ, thus simplifying the link to the vertex model of Section 3. Despite these differences, and thanks to the identical form of solution (S.4), we are able to provide a mapping between the parameters of the two models in equations (S.6).

We now have four parameters (ζ, k, τ, and T) which could have time-dependent values because of the internal effect of increasing levels of keratin. We simplify the fitting problem by assuming that ζ, k, and T are constant on the timescale corresponding to the pipette experiment, leaving only τ variable. The effective relaxation time scale τ_r (S.2b) characterises how the velocity responds to the externally applied force. In the pipette experiment, we expect the effect of keratin to be stronger during the second phase (release) than during the first phase (aspiration). Therefore, we separately fit both phases to functions of the form (S.4) and extract the corresponding relaxation time scales τ_r and τ_r' in the first and second phases respectively (see Supplementary Figure 6C). Supplementary Figure 7A shows $\tau_r' > \tau_r$ by a factor ≈ 2. Possible interpretations may be that the effect of keratin is to decrease the elastic constant k, which is counter-intuitive, or that it increases the relaxation time scale τ (*i.e.* the effective friction coefficient $k\tau$). We will consider the second case. Consequently we formulate these final hypotheses:

1. the relaxation time in the aspiration phase $\tau_r = \zeta\tau/(\zeta + k\tau)$ is related to a first internal relaxation time τ,
2. the velocity before release $\dot{\ell}(t^-_{\text{release}}) = \tau_r'(F^0_{\text{ext}} - T)/\tau'\zeta$ is related to a second relaxation time τ'.

Taken together these allow us to uniquely infer the parameters of (S.2a) from the the measured relaxation

time scales and velocities (Supplementary Figure 7A-B):

$$\tau = \frac{\tau_r \tau_r'}{\tau_r' - \tau_r \left(1 - \frac{\dot{\ell}(t_{\text{release}}^-)}{\dot{\ell}(0^+)}\right)}, \tag{S.5a}$$

$$\tau' = \frac{\dot{\ell}(0^+)}{\dot{\ell}(t_{\text{release}}^-)} \tau_r', \tag{S.5b}$$

$$k = \frac{F_{\text{ext}}^0}{\tau_r'(\dot{\ell}(t_{\text{release}}^-) - \dot{\ell}(t_{\text{release}}^+))} \left(1 - \frac{\dot{\ell}(t_{\text{release}}^-)}{\dot{\ell}(0^+)}\right), \tag{S.5c}$$

$$\zeta = \frac{F_{\text{ext}}^0}{\dot{\ell}(t_{\text{release}}^-) - \dot{\ell}(t_{\text{release}}^+)}, \tag{S.5d}$$

$$T = F_{\text{ext}}^0 \left(\frac{\dot{\ell}(0^+)}{\dot{\ell}(t_{\text{release}}^-) - \dot{\ell}(t_{\text{release}}^+)} - 1\right). \tag{S.5e}$$

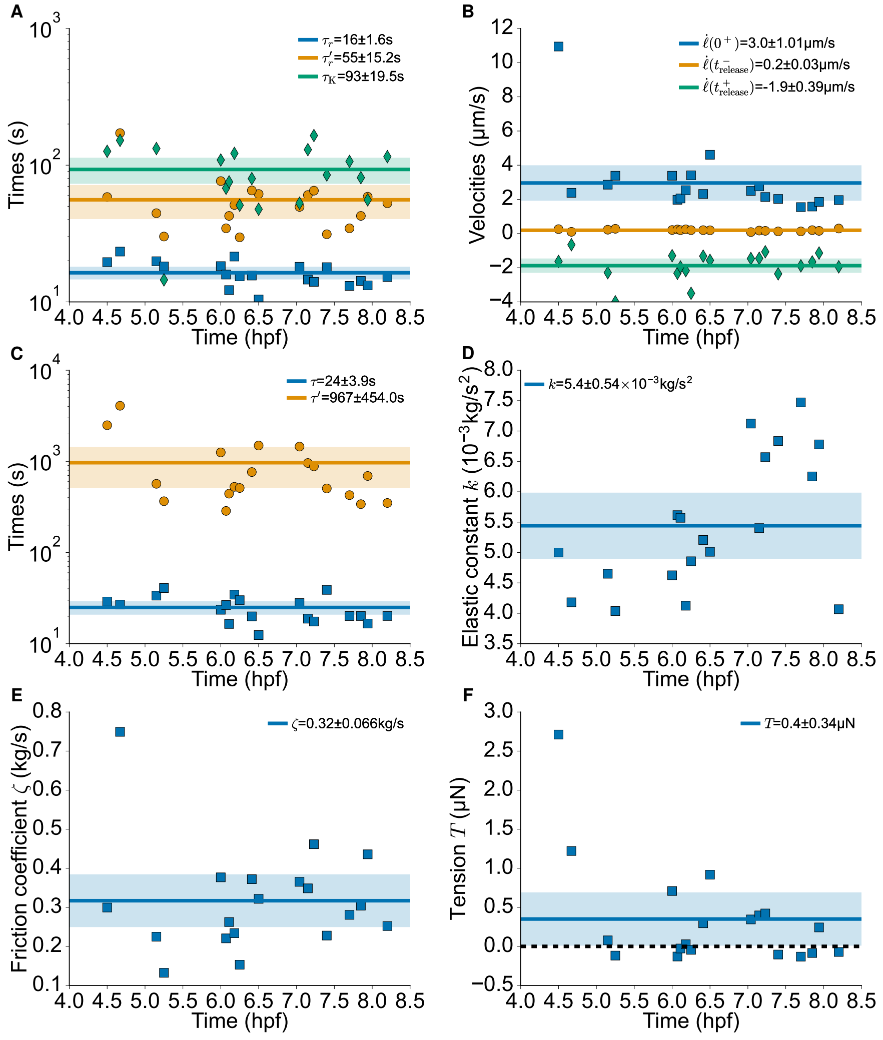

Supplementary Figure 7: (A) Measured time scales τ_r and τ_r' for the relaxation of the velocities $\dot{\ell}(t)$ (Supplementary Figure 6A) in the aspiration and release phases respectively, and measured time scale τ_K for the relaxation of the keratin $k(t)$ (Supplementary Figure 6B). (B) Measured velocities as extracted by linear fits to the height curves $\ell(t)$ (Supplementary Figure 6A) where t_{release} is the time where the pressure is removed. (C) Computed relaxation time scales τ (S.5a) and τ' (S.5b). (D) Tissue elastic constant k (S.5c). (E) Substrate friction coefficient ζ (S.5d). (F) Internal tissue tension T (S.5e).

We plot these quantities in Supplementary Figure 7C-F.

Relation to the viscous drop model

Within the viscous drop model of Refs. [3, 4], the mechanical properties of the tissues are characterised in terms of surface tension γ, which provides the tension which the aspiration force must overcome, and viscosity η, which accounts for viscous dissipation at the opening of the pipette. Using our notations, these can be computed as follows [4]

$$\gamma = \frac{d\,\Delta P_{\text{ext}}\dot{L}_{\text{ret}}}{4(\dot{L}_{\text{ret}} + \dot{L}_{\text{asp}})} = \frac{T}{\pi d}, \tag{S.6a}$$

$$\eta = \frac{d\,\Delta P_{\text{ext}}}{6\pi(\dot{L}_{\text{asp}} + \dot{L}_{\text{ret}})} = \frac{2(\zeta + k\tau)}{3\pi^2 d}, \tag{S.6b}$$

where we have used the aspiration velocity $\dot{L}_{\text{asp}} = \dot{\ell}(t^-_{\text{release}}) = (F^0_{\text{ext}} - T)/(\zeta + k\tau)$ and the retraction velocity $\dot{L}_{\text{ret}} = -\dot{\ell}(t \gg t_{\text{release}}) = T/(\zeta + k\tau)$.

Keratin time scale

We expect the keratin concentration to respond to the stress within the cell. At the simplest level, *i.e.* without postulating a mechanism, we can extract a characteristic time scale for this response from the experimental curves, which we obtain by fitting their second part (after release) to an exponentially decreasing function $t \mapsto \exp(-t/\tau_K)$. This time scale τ_K is larger than the relaxation time scales τ_r and τ_r' (Supplementary Figure 7A), consistent with the wider curves in the keratin than in the height plot (Supplementary Figure 6).

3 Vertex model and feedback

The results of the pipette experiment show that keratin reacts to stress, and that it also affects the elastic properties of the tissue. Together with the fact that keratin forms a growing network of filaments within cells during epiboly, we propose the following feedback mechanisms for keratin filament concentration:

(i) its concentration in a given cell increases with the mechanical stress on this cell due to stress-dependent assembly and disassembly rates,

(ii) in turn the presence of keratin filaments generates a composite material, affecting the viscoelastic response of the cell by increasing both its stiffness (elasticity) and its relaxation time to external stress (viscosity); this effect is only considered above a minimum keratin concentration which translates the fact that keratin must percolate within the cell,

and implement these mechanisms within a vertex model.

Model formulation

We introduce a vertex model interaction potential with perimeter and area elasticity [6, 7]

$$U = \sum_{\text{cells } i} \left[\frac{1}{2}\frac{\Gamma_i}{A_0}\Big(A_i - A_{i,0}\Big)^2 + \frac{1}{2}\Gamma_i\Big(P_i - s_0\sqrt{A_{i,0}}\Big)^2 \right], \tag{S.7}$$

where A_i and P_i are the area and perimeter of cell i respectively (Supplementary Figure 8), Γ_i is an elastic constant, and $s_0 = P_{i,0}/\sqrt{A_{i,0}}$ is the shape index. We introduce viscous relaxation through the cell-wise relaxation of the target area $A_{i,0}$

$$\tau_i \dot{A}_{i,0} = -(A_{i,0} - A_i) \tag{S.8}$$

while enforcing $A_{i,0} = \max(A_0, A_{i,0})$ with a reference area $A_0 > 0$ in order for cells not to disappear.

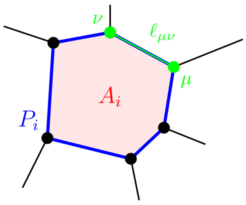

Supplementary Figure 8: A cell in a vertex model.

Mechanism (i), stress-dependent assembly of keratin filaments, is captured generically by the rate equation

$$\tau_K \dot{K}_i = \alpha \max(0, p_i) - K_i, \tag{S.9}$$

where K_i and p_i are the keratin concentration and the stress or in-sheet pressure (S.19) on cell i, and $\alpha > 0$ is the sensitivity of keratin to stress. Under constant stress ($p_i > 0$), the steady state keratin concentration is

$$K_i^{\mathrm{ss}} = \alpha p_i \tag{S.10}$$

which is an increasing function of the stress p_i. Note that we use a sign convention where pulling forces on a cell correspond to positive p_i (see below). We consider initial keratin levels $K_i = 0$ for all cells.

Mechanism (ii), the mechanical effect of keratin, is captured by adjusting stiffness and relaxation according to

$$\Gamma_i = \Gamma\ (1 + \beta \max(0, K_i - K_{\mathrm{th}})), \tag{S.11a}$$

$$\tau_i = \tau\ (1 + \beta \max(0, K_i - K_{\mathrm{th}})), \tag{S.11b}$$

where Γ and τ are the keratin-free elastic constant and relaxation time scale respectively, K_{th} is the threshold in keratin concentration, and β characterises the intensity of the keratin effect on the cell properties. A positive threshold $K_{\mathrm{th}} > 0$ is meant to translate the fact that keratin first needs to percolate through the cell for it to have an effect.

Stress and pressure in the vertex model

In order to define the in-sheet pressure p_i we first need to define the stress tensor. We use the virial stress tensor for a system that is in mechanical equilibrium [8],

$$\underline{\boldsymbol{\sigma}}^T = \frac{1}{2V} \sum_i \sum_{j,\langle ij \rangle} \boldsymbol{r}_{ij} \otimes \boldsymbol{F}_{ij}, \tag{S.12}$$

where $\langle ij \rangle$ indicates that i and j are neighbours such that the sum is now over all contacts of particle i, $\boldsymbol{r}_{ij} = \boldsymbol{r}_j - \boldsymbol{r}_i$, $\otimes$ denotes an outer product, and there is a 2 because of double-counting. We convert our packing into a three-dimensional tiling around point particles, with volumes V_i, and from there define a local stress tensor

$$\underline{\boldsymbol{\Sigma}}^i = \frac{1}{2} \sum_{j,\langle ij \rangle} \boldsymbol{r}_{ij} \otimes \boldsymbol{F}_{ij},\ \underline{\boldsymbol{\sigma}}^i = \frac{\underline{\boldsymbol{\Sigma}}^i}{V_i}. \tag{S.13}$$

Here we have introduced the local force moment tensors $\underline{\boldsymbol{\Sigma}}_i$, which are additive over the system, *i.e.* $\underline{\boldsymbol{\Sigma}}^T = \sum_i \underline{\boldsymbol{\Sigma}}^i$. The total stress tensor is then simply $\underline{\boldsymbol{\sigma}}^T = \sum_i \underline{\boldsymbol{\Sigma}}^i / V$.

We consider a three-dimensional flat tissue, *i.e.* where all forces are applied in the plane, in which each cell conserves its volume $V_i = V_0$ by stretching or shrinking in the dimension orthogonal to the plane. We introduce the area and perimeter forces deriving from the interaction potential (S.7)

$$\boldsymbol{F}_{i\mu} = -\frac{1}{2}\frac{\Gamma_i}{A_0}(A_i - A_{i,0})(\boldsymbol{r}_{(\mu+1)(\mu-1)} \times \hat{\boldsymbol{e}}_z), \tag{S.14a}$$

$$\boldsymbol{T}^i_{\mu\nu} = \Gamma_i (P_i - s_0 \sqrt{A_{i,0}}) \hat{\boldsymbol{r}}_{\mu\nu}, \tag{S.14b}$$

with $\hat{\boldsymbol{r}}_{ij} = \boldsymbol{r}_{ij}/l_{ij},\ l_{ij} = |\boldsymbol{r}_{ij}|$, $\times$ designating a vector cross product, and where Greek indices of vertices around the cell centre are ordered with trigonometric orientation, and thus $\boldsymbol{r}_{(\mu+1)(\mu-1)} \times \hat{\boldsymbol{e}}_z$ is expected to point from cell corner μ towards cell centre i. $\boldsymbol{F}_{i\mu}$ is the force applied to cell centre i by cell corner μ, and $\boldsymbol{T}^i_{\mu\nu}$ is the contribution from cell i to the force applied to cell corner μ by cell corner ν (with μ and ν belonging to cell i).

We may consider cell centres $\boldsymbol{r}_i$ and cell corners $\boldsymbol{r}_\mu$ separately, and define tiling volumes V_i and V_μ separately for them. We can then write for the centres

$$\underline{\underline{\boldsymbol{\sigma}}}^i = \frac{\underline{\underline{\boldsymbol{\Sigma}}}^i}{V_i} = \frac{1}{2V_i} \sum_{\text{corners } \mu, \langle i\mu\rangle} \boldsymbol{r}_{i\mu} \otimes \boldsymbol{F}_{i\mu}, \tag{S.15}$$

where we have assumed that the forces $\boldsymbol{F}_{i\mu}$ are pairwise between cell corners and cell centres; this procedure is harmless since the sum $\sum_\mu \boldsymbol{F}_{i\mu}$, taken over all corners μ of cell cell i, cancels exactly. Meanwhile, for the vertices (cell corners) we have

$$\underline{\underline{\boldsymbol{\sigma}}}^\mu = \frac{\underline{\underline{\boldsymbol{\Sigma}}}^\mu}{V_\mu} = \frac{1}{2V_\mu} \left[\sum_{\text{cells } i, \langle \mu i\rangle} \boldsymbol{r}_{\mu i} \otimes \boldsymbol{F}_{\mu i} + \sum_{\text{corners } \nu, \langle \mu\nu\rangle} \boldsymbol{r}_{\mu\nu} \otimes \sum_{\text{cells } i, (\mu\nu)\in i} \boldsymbol{T}^i_{\mu\nu} \right]. \tag{S.16}$$

As written, this defines an extensive force-moment tensor $\underline{\underline{\boldsymbol{\Sigma}}}^T = \sum_i \underline{\underline{\boldsymbol{\Sigma}}}^i + \sum_\mu \underline{\underline{\boldsymbol{\Sigma}}}^\mu = \sum_i V_i \underline{\underline{\boldsymbol{\sigma}}}^i + \sum_\mu V_\mu \underline{\underline{\boldsymbol{\sigma}}}^\mu$, and then a well defined stress tensor $\underline{\underline{\boldsymbol{\sigma}}}^T = \underline{\underline{\boldsymbol{\Sigma}}}^T / V$.

In order to define a stress tensor that is defined per *cell*, we define $\underline{\underline{\tilde{\boldsymbol{\Sigma}}}}^i = \underline{\underline{\boldsymbol{\Sigma}}}^i + \sum_{\langle i\mu\rangle} \underline{\underline{\boldsymbol{\Sigma}}}^{\mu|i}$, that is the centre part of the stress from (S.15), plus the part of the vertex-stress from all surrounding vertices that is linked to cell i, we have

$$\begin{aligned} \underline{\underline{\tilde{\boldsymbol{\Sigma}}}}^i = V_i \underline{\underline{\tilde{\boldsymbol{\sigma}}}}^i &= \frac{1}{2} \sum_{\text{corners } \mu, \langle i\mu\rangle} \left[2\boldsymbol{r}_{i\mu} \otimes \boldsymbol{F}_{i\mu} + \boldsymbol{r}_{\mu(\mu+1)} \otimes \boldsymbol{T}^i_{\mu(\mu+1)} + \boldsymbol{r}_{\mu(\mu-1)} \otimes \boldsymbol{T}^i_{\mu(\mu-1)} \right] \\ &= \sum_{\text{corners } \mu, \langle i\mu\rangle} \left[\boldsymbol{r}_{i\mu} \otimes \boldsymbol{F}_{i\mu} + \boldsymbol{r}_{\mu(\mu+1)} \otimes \boldsymbol{T}^i_{\mu(\mu+1)} \right]. \end{aligned} \tag{S.17}$$

The factor of 2 comes from the centre forces also being included in (S.16) and action-reaction. This then does give an additive force moment tensor $\underline{\underline{\boldsymbol{\Sigma}}}^T = \sum_i \underline{\underline{\tilde{\boldsymbol{\Sigma}}}}^i = \sum_i V_i \underline{\underline{\tilde{\boldsymbol{\sigma}}}}^i = V \underline{\underline{\boldsymbol{\sigma}}}^T$ with the same value as the one from equation S.12. We write the cell stress tensor

$$\underline{\underline{\tilde{\boldsymbol{\sigma}}}}^i = \frac{1}{V_i} \sum_{\text{corners } \mu, \langle i\mu\rangle} \left[\frac{1}{2} K(A_i - A_0) \boldsymbol{r}_{i\mu} \otimes (\boldsymbol{r}_{(\mu-1)(\mu+1)} \times \hat{\boldsymbol{e}}_z) + \Gamma(P_i - P_0) \boldsymbol{r}_{\mu(\mu+1)} \otimes \hat{\boldsymbol{r}}_{\mu(\mu+1)} \right] \tag{S.18}$$

and cell pressure

$$p_i = \mathrm{Tr}(\underline{\underline{\tilde{\boldsymbol{\sigma}}}}^i) = \frac{1}{V_0} \left[\frac{\Gamma_i}{A_0} (A_i - A_{i,0}) A_i + \Gamma_i (P_i - s_0 \sqrt{A_{i,0}}) P_i \right], \tag{S.19}$$

where we have used $V_i = V_0$ for all i.

What we call pressure in (S.19) is more properly a biaxial shear stress in the plane of the sheet, as all the forces and vectors remain in the plane. If we assume that due to the volume constraint, the incompressible xy sheet simply thins in the orthogonal z direction when pulled, we have $\underline{\underline{\tilde{\boldsymbol{\sigma}}}}^i_{kz} = 0$ for all components k, and $p_i = \sigma^i_{xx} + \sigma^i_{yy}$. With our sign conventions, the pressure or stress is positive for a sheet under outward tension, and negative for a sheet under compression.

The vertex model stress (S.19) contains area and perimeter terms, which partially compensate each other in a typical cell, with tension on the junctions being balanced by area compression. It is highly sensitive to changes in this balance, and in particular, the stress of boundary cells with different shapes and with applied pulling forces can have unexpected geometry / force feedback. Therefore, for numerical stability, we use only the area part of the stress to implement keratin feedback for the outer ring of boundary cells.

Matching parameters and epiboly simulation

We finally write the equation of motion for vertex μ

$$\zeta \dot{\boldsymbol{r}}_\mu = -\frac{\partial}{\partial \boldsymbol{r}_\mu} U + \boldsymbol{F}_\mu^{\text{pull}}, \tag{S.20}$$

where $\boldsymbol{F}_\mu^{\text{pull}}$ is an external pulling force that expands the tissue, simulating the effect of the YSL. We define boundary vertices as the ensemble of vertices at the open outer edge of the tissue. In order to keep the tissue round, we use the following pulling force

$$\boldsymbol{F}_\mu^{\text{pull}} = \begin{cases} \frac{N_{\text{edges}} F_{\text{YSL}}}{\sum_\mu |\boldsymbol{\ell}_{\mu-1\to\mu} + \boldsymbol{\ell}_{\mu\to\mu+1}|} \left(\boldsymbol{\ell}_{\mu-1\to\mu} + \boldsymbol{\ell}_{\mu\to\mu+1}\right) \times \hat{\boldsymbol{e}}_z & \text{if } \mu \text{ is a boundary vertex,} \\ 0 & \text{otherwise,} \end{cases} \tag{S.21}$$

where N_{edges} is the number of boundary vertices, indexed by μ in anticlockwise order, with $\boldsymbol{\ell}_{\mu\to\nu} = \boldsymbol{r}_\nu - \boldsymbol{r}_\mu$.

We estimate the model parameters (Supplementary Table 1) as follows: We use the same elastic constant $k = \Gamma$ and friction coefficient ζ. We use a relaxation time scale $\tau = 500s$ for the vertex model with a value than the fitted pipette time scale $\tau = 24s$ but still smaller than $\tau' \approx 1000s$ of the Maxwell model.

Here it is important to emphasize that biological tissues do not behave as simple, linear viscoelastic materials. Even in the absence of keratin, tissue rheology is inherently complex. Pipette aspiration experiments involve high stress gradients, which induce substantial cell deformations and promote cell rearrangements (see, for example, Tlili et al. in [9]). In contrast, the mechanical stresses generated by the yolk syncytial layer (YSL) during epiboly are more spatially and temporally homogeneous. Under these conditions, we observed minimal cell rearrangements and limited shape deformations.

Consequently, parameters such as the relaxation time τ (and by extension, the viscosity) in the vertex model are expected to differ from those inferred from pipette aspiration. In particular, for a material with a yield stress - such as the tissue studied here, at least with respect to rearrangements - τ can be significantly larger. This consideration and matching the epiboly experiments underlies our choice of choosing $\tau = 500$ in the vertex model.

We fix the shape index $s_0 = 3.72$ so that the tissue is in a solid state in the absence of viscous relaxation [7]. We estimate the reference cell area to $A_0 = 600\,\mu\text{m}^2$ and the reference cell height $h_0 = 30\,\mu\text{m}$ so that the reference cell volume $V_0 = h_0 A_0 = 18 \times 10^3\,\mu\text{m}^3$.

We estimate the coefficient α from the pipette experiment by estimating the response in keratin concentration to a pressure perturbation. From Fig. 6, we see a response of keratin of the order of $\tilde{K}^{ss} \approx 16$. However, pipette and epiboly experiments are imaged differently. In the pipette experiment,

Maxwell model	Vertex model
$\tau = 24.0 \pm 3.9\,\text{s}$<br>$\tau' = 967 \pm 454\,\text{s}$	$\tau = 500\,\text{s}$
$k = 5.40 \pm 0.54 \times 10^3\,\text{kg}\,\text{s}^{-2}$	$\Gamma = 5.4 \times 10^3\,\text{kg}\,\text{s}^{-2}$
$\zeta = 0.320 \pm 0.066\,\text{kg}\,\text{s}^{-1}$	$\zeta = 0.32\,\text{kg}\,\text{s}^{-1}$
$T = 0.40 \pm 0.34\,\mu\text{N}$<br>$F_{\text{ext}} = 0.57\,\mu\text{N}$	$F_{\text{YSL}} = 0.57\,\mu\text{N}$
$\tau_K = 93.0 \pm 19.5\,\text{s}$	$\tau_K = 93\,\text{s}$
	$s_0 = 3.72$
	$A_0 = 600\,\mu\text{m}^2$
	$V_0 = 18 \times 10^3\,\mu\text{m}^3$
	$\alpha \sim \frac{\Delta K^{\text{ss}}}{P_{\text{pipette}}} \approx 1.2 \times 10^5\,\text{kg}^{-1}\,\text{s}^2$
	$\beta = 0$ (keratin loss-of-function), $\beta = 0.005$ (wild type)

Supplementary Table 1: Parameter values of the Maxwell model (Supplementary Figure 7) and the vertex model.

only one confocal slice is considered, while 15 are added in the epiboly imaging, we thus apply a conversion coefficient of about 15, $K^{\rm ss} = 15\tilde{K}^{ss} = 240$; this value is consistent with the range of keratin values observed experimentally. We consider a reference in-sheet pressure $p \sim \Delta P_{\rm ext} = 2\,\rm mbar$, such that using (S.10) we obtain $\alpha \sim K^{\rm ss}/\Delta P_{\rm ext} \approx 1.2 \times 10^5\,\rm kg^{-1}\,s^2$. Then β remains as a free parameter which we can adjust to match the experimental observations.

We generate initial disordered configurations by applying an active Brownian force $\boldsymbol{F}_\mu^{\rm AB}$ on all vertices and an additional boundary tension force $\boldsymbol{F}_\mu^{\rm BT}$ on boundary vertices to keep the tissue round. We define the active Brownian force

$$\boldsymbol{F}_\mu^{\rm AB} = v_0 \begin{pmatrix} \cos\theta_\mu \\ \sin\theta_\mu \end{pmatrix}, \tag{S.22a}$$

$$\dot{\theta}_\mu = \sqrt{2/\tau_p}\,\eta_\mu, \tag{S.22b}$$

where η_μ a Gaussian white noise with variance $\langle \eta_\mu(0)\eta_\nu(t)\rangle = \delta_{\mu\nu}\,\delta(t)$, and the boundary tension force

$$\boldsymbol{F}_\mu^{\rm BT} = \gamma \left(\frac{\boldsymbol{\ell}_{\mu\to\mu-1}}{|\boldsymbol{\ell}_{\mu\to\mu-1}|} + \frac{\boldsymbol{\ell}_{\mu\to\mu+1}}{|\boldsymbol{\ell}_{\mu\to\mu+1}|} \right). \tag{S.23}$$

which only acts on outer boundary vertices. We used the interaction potential (S.7) with parameters $\Gamma_i = 1$, $A_{i,0} = 1$, $s_0 = 3.72$, the active Brownian force (S.22) with parameters $v_0 = 0.1$, $\tau_p = 1$, and the boundary tension force (S.23) with parameter $\gamma = 0.3$. We perform a first initial run to reach steady state, then remove the active Brownian force and perform a second run to reach a force equilibrium between the interaction forces (S.7) and the boundary tension forces (S.23). We use this final configuration to initialise the simulation with the parameters from Supplementary Table 1. We rescale the size of the system to minimise $|\sum_{\text{vertices } \mu} (\partial_{\boldsymbol{r}_\mu} U) \cdot (\boldsymbol{r}_\mu - \boldsymbol{r}^{\rm CM})|$, where $\boldsymbol{r}^{\rm CM} = \sum_{\text{vertices } \mu} \boldsymbol{r}_\mu / N_{\rm v}$ is the position of the centre of mass and N_v the number of vertices. This latter rescaling ensures that the tissue does not initially move unless it is being pulled.

4 Model tissue cell ablation

We prepare model tissue cell ablation simulations by first generating a disordered configuration of the vertex model with periodic boundary conditions. To this effect we use the potential energy (S.7) with parameters $\Gamma_i = 1$, $A_{i,0} = 1$, $s_0 = 3.81$ and the active Brownian force (S.22) with parameters $v_0 = 0.25$, $\tau_p = 1$. We perform a first initial run to reach steady state. We then remove the active Brownian force and rescale the system such that the mean area of the cells is $\overline{A} = 1750\,\rm \mu m^2$, and set the other parameters to the values in Supplementary Table 1. We set the target areas of the cells at $A_{i,0} = A_i/1.41$ but keep $\tau_i = \infty$ in (S.8), and let the system equilibrate to a steady state in keratin intensity. At $t = 0$ we set $\tau_i = 500\,\rm s$, ablate 6 cells, and apply a boundary force (S.23) around the ablated cells with $\gamma = 2\,\rm \mu N$.

5 Keratin feedback regulates velocity

To understand the keratin expression dynamics and edge speeds, we turn to simple mean field dynamical equations. If we return to the dynamics of a single junction, now as part of epiboly, we have

$$\zeta\dot{\ell} = -k(\ell - \ell_0) + F_{\rm YSL}, \tag{S.24a}$$

$$\tau\dot{\ell}_0 = \ell - \ell_0, \tag{S.24b}$$

with keratin dynamics

$$\tau_K \dot{K} = \alpha \max(0, p) - K. \tag{S.25}$$

The keratin then influences the stiffness and relaxation constants equally through

$$k = k_0(1 + \beta\max(0, K - K_{th})), \tag{S.26a}$$

$$\tau = \tau_0(1 + \beta\max(0, K - K_{th})). \tag{S.26b}$$

Here, to match the stress in the vertex model, we write the tissue stress or pressure as

$$p = k\frac{(\ell - \ell_0)\ell}{V_0} \tag{S.27}$$

and we subsequently use the same parameters as in the vertex model.

Supplementary Figure 9 shows the dynamics of this mean field model. As in the main text, we perform a linear force ramp over 11 hours for different values of F_{YSL}, both above and below the expected value of $0.57\mu N$ for a wild-type embryo. Panel B show the keratin dynamics, which is quantitatively close to the full model and the experiment. Panel C shows the rate of junction expansion $\dot{\ell}$. As in the full model, it quickly rises proportional to pulling force, and then has a peak when the feedback kicks in when $K \geq K_{\mathrm{th}}$. Junction expansion rate $\dot{\ell}$ can be related to the edge speed through the scaling $v_{\mathrm{edge}} \approx \sqrt{N}\dot{\ell}$, *i.e.* multiply by a factor of 23. Again, the dynamics is quantitatively similar to the full vertex model, though the peak is sharper. The smoothing is therefore due to the strong heterogeneity in keratin expression in the full simulation. If we turn off the feedback ($\beta = 0$), just as in the vertex model, the rate of junction expansion is larger and increases indefinitely instead (panel D). In summary, the mean field model captures the essential features of the full vertex model.

We can understand the origin of the feedback regulation in this simplified setting: In epiboly, we expect pressure to be positive, *i.e.* a tissue under tension, so we can replace $\max(0, p) = p$. We can then define the new variables $\Delta K = K - K_{th}$ and $u = \ell - \ell_0$, the current deformation from the mechanical equilibrium value. For $\Delta K > 0$ we then write

$$\dot{u} = -\left[\frac{k_0}{\zeta}(1 + \beta\Delta K) + \frac{1}{\tau_0(1 + \beta\Delta K)}\right] u + \frac{F_{\mathrm{YSL}}}{\zeta} \tag{S.28a}$$

$$\Delta\dot{K} = \frac{\alpha k_0 a}{V_0}(1 + \beta\Delta K)u - \frac{1}{\tau_K}(\Delta K + K_{th}) \tag{S.28b}$$

and the same equation with $\beta = 0$ for $\Delta K < 0$. We have used $\ell\,(\ell - \ell_0) \approx a\,u$, where $a = \mathcal{O}(\ell)$ is a constant length scale in the model, under the assumption that ℓ is slowly varying. This slight simplification makes the model tractable.

We are mostly interested in the long-term steady-state evolution set by the fixed points of the dynamics, where $\dot{K} = 0$ and $\dot{u} = 0$ and so the tissue expands at constant keratin concentration. The fixed point (and there is only one) is set by the intersections of the nullclines, *i.e.* the lines where separately $\dot{u} = 0$ (u-nullcline) and $\dot{K} = 0$ (K-nullcline). Their equations are given by:

$$u_{\dot{u}=0} = \frac{F_{\mathrm{YSL}}\tau_0}{\zeta}\frac{1 + \beta\Delta K}{1 + \frac{k_0\tau_0}{\zeta}(1 + \beta\Delta K)^2} \tag{S.29a}$$

$$u_{\dot{K}=0} = \frac{\Delta K + K_{th}}{\frac{\alpha k_0 a}{V_0}(1 + \beta\Delta K)} \tag{S.29b}$$

for $\Delta K > 0$ and the same with $\beta = 0$ for $\Delta K < 0$. The edge speed is given by $\dot{\ell} = \dot{u} + \dot{\ell}_0 = -\frac{u^*}{\tau(\Delta K^*)}$, where the last equality is valid at the fixed point (K^*, u^*).

Supplementary Figure 9A shows the simulated dynamics replotted in $(\Delta K, u)$ coordinates. We have added the nullclines of the keratin dynamics ΔK (solid lines) and of the junction dynamics u (dashed lines), both plotted for the end values of the simulations for F_{YSL} and a. Here the junction nullcline moves up and left with increasing pulling force. The keratin nullcline depends only slightly on ℓ, in contrast.

The following picture emerges: At low pulling forces, *i.e.* either early in the simulation or if F_{YSL} is small, the junction nullcline intersects the keratin nullcline for $\Delta K < 0$, where it is linearly increasing as a function of ΔK. This corresponds to the combined upwards ramp of keratin and junction speed that we observe at early times, and to a model with no feedback. At higher force, the junction nullcline intersects the keratin nullcline at $\Delta K > 0$, where the curve becomes nearly flat. Thus we find the characteristic dynamics of feedback: Independent of pulling force, we recover approximately the same u^* at the fixed point, while ΔK^* keeps increasing with pulling force. This translates to reducing $\dot{\ell}$ with pulling force as $\dot{\ell} = -\frac{u^*}{\tau(\Delta K^*)}$ and τ increases with ΔK^*.

In contrast, when we turn off feedback ($\beta = 0$), $\dot{\ell}$ keeps increasing indefinitely with pulling force, and one can easily compute that $\dot{\ell} = \frac{F_{\mathrm{YSL}}}{\zeta + k_0\tau_0}$ at the fixed point.

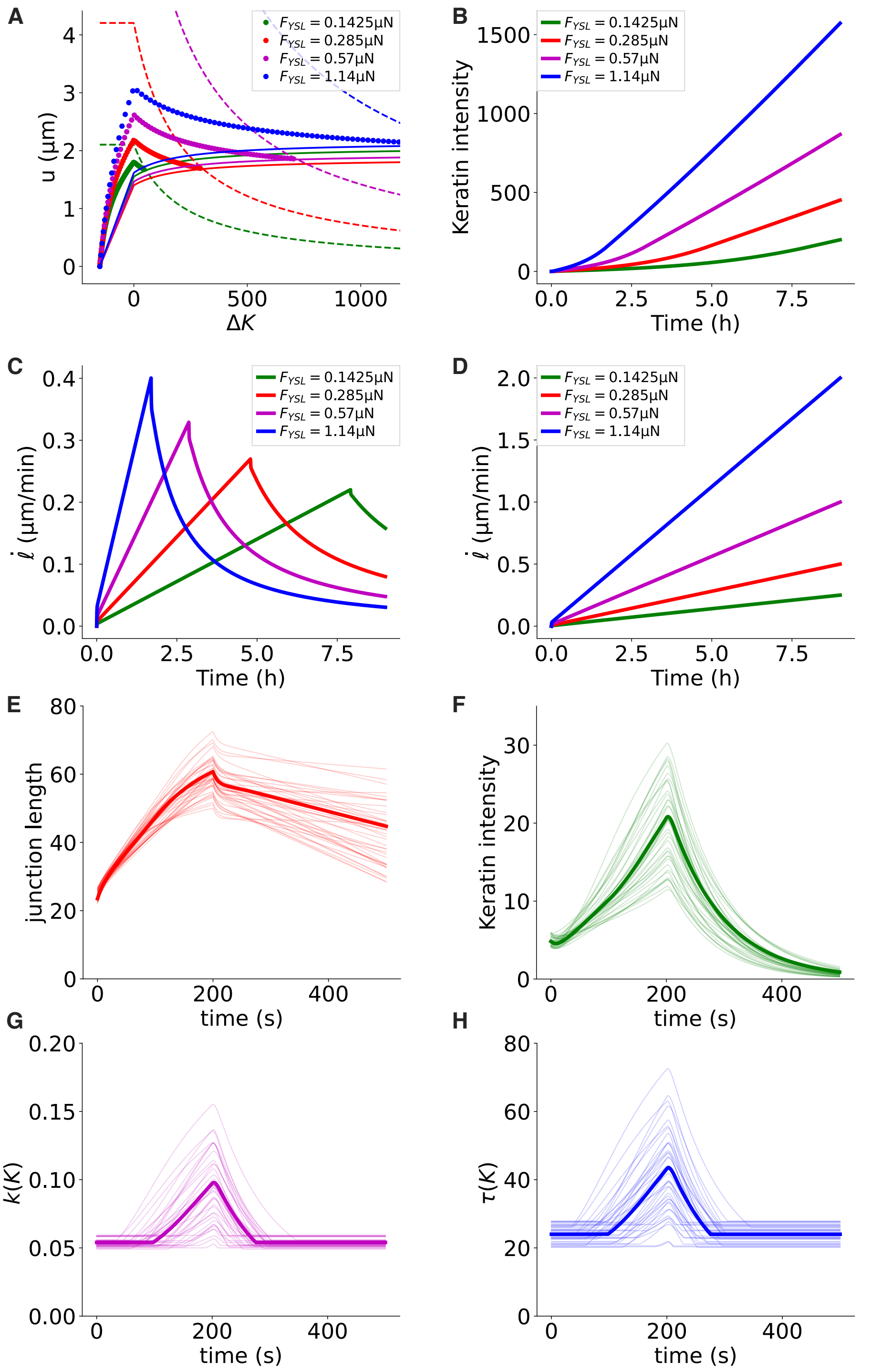

Supplementary Figure 9: Mean-field model dynamics. (A-D): Epiboly simulation. As in the main text, we perform a linear force ramp for four values of F_{YSL}. (A) Reduced variable phase space $(\Delta K, u)$. Simulations with feedback (wild type, $\beta = 0.005$) are shown as dots, together with the nullclines of ΔK (solid) and of $u = \ell - \ell_0$ (dashed). (B) Keratin dynamics and (C) junction length expansion speed $\dot{\ell}$ as a function of time. (D) For the 'mutant' keratin loss-of-function model with no feedback ($\beta = 0$), $\dot{\ell}$ is not regulated and increases with F_{YSL}. (E-H): Pipette pulling simulation. We apply the pipette force $F = 0.57\mu N$ from $t = 0$ to $t = 200s$, releasing the system afterwards. Thick lines correspond to the estimated model parameters, thin lines correspond to 40 samples within error bars. (A) Junction length, (B) keratin intensity (rescaled by $c = 15$ to account for the imaging differences), (C) stiffness k and (D) relaxation time τ.

6 The pipette experiment in the mean field model

The fitting of the pipette curves with the visco-elastic Maxwell model allowed us to extract estimates of the elastic, viscous and relaxation scales. It separately gave us a very rough estimate of α, the responsiveness of keratin expression to stress. It did not constrain β, the strength of the mechanical response to keratin. We therefore now return to the pipette experiment using the mean field model, to check for internal consistency and to develop the next order approximation of the model for the pipette.

As before, starting from a resting tissue under internal tension T, we apply a pressure of $p = 2\,\text{mbar}$ to the tissue, and then remove it at $t = 200\,\text{s}$. We use the fitted values of parameters ζ, τ, k and τ_K as well as T from Supplementary Table 1, and pick 40 uniformly random samples within the error bars of these parameters in addition to the mean value. For each of these values, we ran one simulation with values of $\alpha = 1.2 \pm 0.2 \times 10^5\,\text{s}^2\,\text{kg}^{-1}$ and $\beta = 0.005 \pm 0.002$ chosen within their own estimated error bars. The results agree within reason with the pipette observations, see Supplementary Figure 9E-H. We find that stiffness k and time scale τ increase by an approximate factor of 2 under maximum pipette pulling conditions.

We note that it overestimates the decay in keratin when the pipette is released – hinting toward a non-reversible part of the keratin network formation. Since during epiboly, stress only increases, there is no effect on our epiboly model simulations.

7 Keratin and stress heterogeneities in epiboly simulations

We provide supplementary video `stretching.mp4` of an epiboly simulation in the wild-type condition ($\beta = 0.005$) with $F_{\text{YSL}} = 0.57\,\mu\text{N}$, showing both the cell-level keratin expression K_i on the left and the stress p_i on the right. This shows qualitatively that both the keratin concentration and the stress first increase at the edge of the tissue where it is being stretched and then radiate inwards towards the animal pole at the centre of the model tissue.

We make this observation quantitative by computing the averages of K_i and p_i over cells within given bins of radii from the tissue centre (see Fig. 10A-B). We define the scaled radius of each bin at each time as the average radius in the bin divided by the maximum distance between a cell and the centre of the tissue at that same time, such that 0 corresponds to the tissue centre and 1 corresponds to the tissue edge. Both of these quantities show a clear gradient from the centre of the tissue to its outer boundary, consistently with tissue having a net zero velocity at its centre and being pulled outward at its boundary. It is noteworthy that the similar behaviour of K_i and p_i derives from the fact that these quantities are highly correlated (see Fig. 10C). This correlation in turn derives from the small keratin relaxation time ($\tau_K = 93\,\text{s}$, see Supplementary Table 1) compared to the relaxation time of the target area ($\tau = 500\,\text{s}$) and given the slow extension of the tissue over several hours; it follows that K_i relaxes fast to the value prescribed by the stress p_i following (S.10).

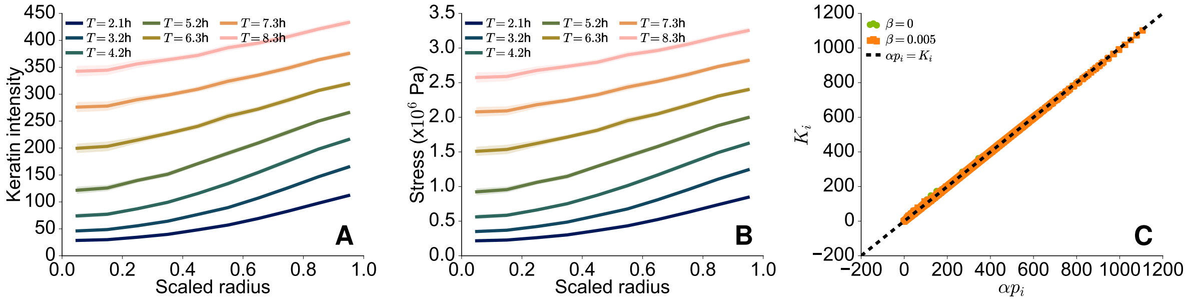

Supplementary Figure 10: Binned averages of (A) keratin concentration K_i and (B) stress p_i as functions of the distance of cells from the tissue centre. The scaled radius is the cell distance from the tissue centre divided by its maximum value at a given time. These are averaged over different realisations of epiboly (stretching) simulations at identical times T since the initial $K_i = 0$ state. (C) Scatter plot of keratin concentration K_i and stress p_i measured over stretching simulations at $\beta = 0$ and 0.005.

8 Enhancing keratin heterogeneities

We established that the heterogeneity of keratin expression is related to the mechanical heterogeneity of the tissue. It is noteworthy that we can enhance keratin heterogeneities, resulting in wider keratin distributions, by either altering the mechanical properties of the system (see Fig. 11) or by adding an additional source of heterogeneities, *e.g.* in mechanosensitivity (see Fig. 12).

We present in Fig. 11 snapshots of epiboly simulations in the wild-type scenario using different values of the shape index s_0. Systems with $s_0 \geq 3.81$ are not (linearly) rigid and have stress-free floppy modes. Pulling still leads to stress in the system, but without notable heterogeneities, and the keratin response is nearly homogeneous. The opposite is true for $s_0 < 3.81$: states of self-stress with heterogeneous patterns become prominent, which is also visible in the keratin response, and is in fact amplified by the keratin-stress feedback loop.

We present in Fig. 12 snapshots of epiboly simulations using either $\alpha = 1.2 \times 10^5$ for all cells (homogeneous α) or values of α randomly distributed among cells (heterogeneous α) according to a normal distribution of mean $m = 1.2 \times 10^5$ and standard deviation $s = 0.2m$ (we also set $\alpha = 0$ when the randomly picked value is negative). This heterogeneity in the sensitivity of keratin to stress translates the likely intercellular variability in the actual biological system. With this enhancement, we recover

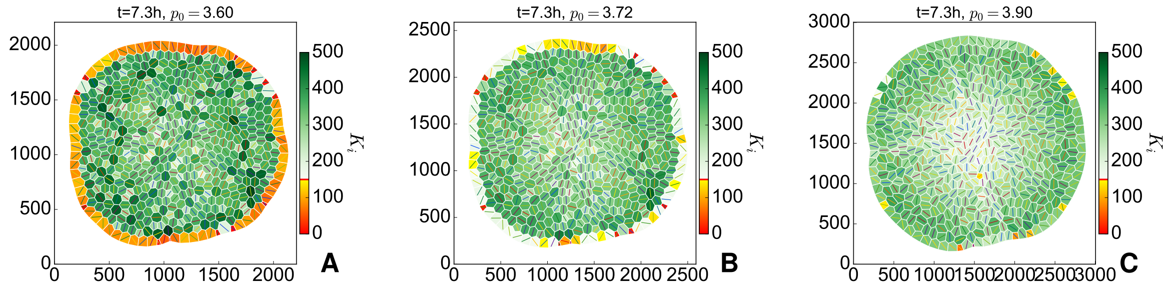

Supplementary Figure 11: Epiboly simulation snapshots in the wild-type scenario ($\beta = 0.005, \alpha = 1.2 \times 10^5$) showing keratin expression levels K_i in each cell. $s_0 =$ (A) 3.6 (B) 3.72 (C) 3.9.

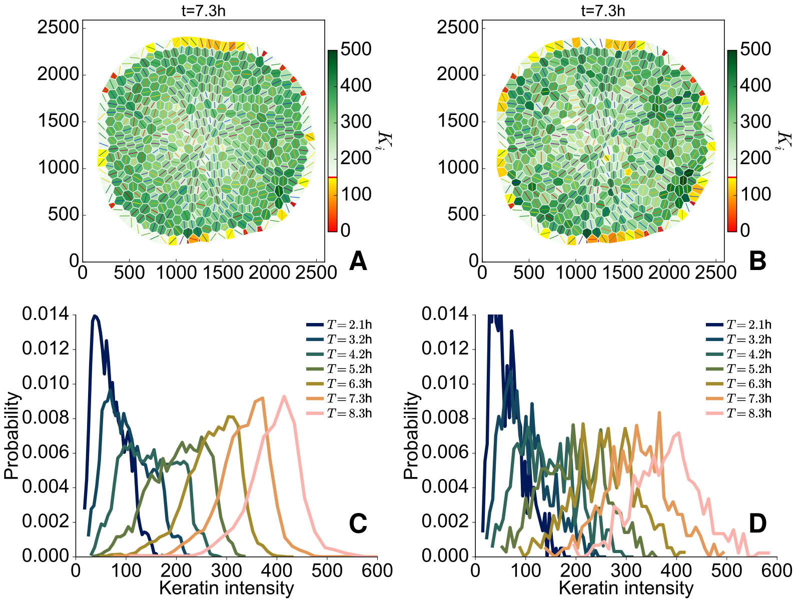

Supplementary Figure 12: (A, B) Epiboly simulation snapshots in the wild-type scenario ($\beta = 0.005$, $\overline{\alpha} = 1.2 \times 10^5$) showing keratin expression levels K_i in each cell. (C, D) Keratin level distributions at different times. (A, C) Homogeneous and (B, D) heterogeneous α.

keratin distributions closer to our experimental measurements.

References

[1] Martin Behrndt, Guillaume Salbreux, Pedro Campinho, Robert Hauschild, Felix Oswald, Julia Roensch, Stephan W. Grill, and Carl-Philipp Heisenberg. Forces Driving Epithelial Spreading in Zebrafish Gastrulation. *Science*, 338(6104):257–260, October 2012.

[2] Pedro Campinho, Martin Behrndt, Jonas Ranft, Thomas Risler, Nicolas Minc, and Carl-Philipp Heisenberg. Tension-oriented cell divisions limit anisotropic tissue tension in epithelial spreading during zebrafish epiboly. *Nature Cell Biology*, 15(12):1405–1414, December 2013.

[3] Karine Guevorkian, Marie-Josée Colbert, Mélanie Durth, Sylvie Dufour, and Françoise Brochard-Wyart. Aspiration of Biological Viscoelastic Drops. *Physical Review Letters*, 104(21):218101, May 2010.

[4] K. Guevorkian and J.-L. Maître. Micropipette aspiration. In *Methods in Cell Biology*, volume 139, pages 187–201. Elsevier, 2017.

[5] Danton Gutierrez-Lemini. *Engineering Viscoelasticity*. Springer US, Boston, MA, 2014.

[6] Alexander G. Fletcher, Miriam Osterfield, Ruth E. Baker, and Stanislav Y. Shvartsman. Vertex Models of Epithelial Morphogenesis. *Biophysical Journal*, 106(11):2291–2304, June 2014.

[7] Daniel L. Barton, Silke Henkes, Cornelis J. Weijer, and Rastko Sknepnek. Active Vertex Model for cell-resolution description of epithelial tissue mechanics. *PLOS Computational Biology*, 13(6):e1005569, June 2017.

[8] Silke Henkes and Bulbul Chakraborty. Statistical mechanics framework for static granular matter. *Physical Review E—Statistical, Nonlinear, and Soft Matter Physics*, 79(6):061301, 2009.

[9] Sham L Tlili, François Graner, and Hélène Delanoë-Ayari. A microfluidic platform to investigate the role of mechanical constraints on tissue reorganization. *Development*, 149(20):dev200774, 2022.
