# Supplementary material for: Keratins coordinate tissue spreading by balancing spreading forces with tissue material properties
Source: Nat Commun. 2026 May 16;17:6499. doi: 10.1038/s41467-026-72366-z (PMC13377027; doi:10.1038/s41467-026-72366-z)
Supplement: Supplementary file 2 — Description of Additional Supplementary Files [file 41467_2026_72366_MOESM2_ESM.pdf]

Title: Supplementary Movie 1:

Description: Keratin expression within the EVL during epiboly: Time-lapse of keratin expression (right) and network organization (left) in Tg*(krt18:Krt18GFP)* embryos during epiboly (4 - 9.5 hpf). Lateral view. Frame rate 11.25 min/frame. Scale bar: 100 (left) and 30 µm (right).

Title: Supplementary Movie 2

Description: Keratin expression within the EVL in embryos with reduced YSL pulling force: Time-lapse of keratin expression within the EVL upon reduced pulling forces from the YSL in a representative Tg*(krt18:Krt18-GFP)* control embryo injected with 0.2% phenol red (left) and an embryo injected with 100 pg *CAMypt* into the YSL (right) imaged from from 4.5-11 hpf. Lateral view. of the embryos Frame rate 10 min/frame. Scale bar: 100 µm.

Title: Supplementary Movie 3

Description: Keratin network maturation in EVL cells of embryos with reduced YSL pulling force: Time-lapse of keratin network maturation in EVL cells upon reduced pulling forces from the YSL in a representative Tg*(krt18:Krt18-GFP)* control embryo injected with 0.2% phenol red (left) and an embryo injected with 100 pg *CAMypt* into the YSL (right) imaged from 4.5-7.1 hpf. Lateral view near the EVL-YSL boundary. Frame rate 10 min/frame. Scale bar: 25 µm.

Title: Supplementary Movie 4

Description: Keratin expression within the EVL of embryos with enhanced YSL pulling force: Time-lapse of keratin expression upon increased pulling forces from the YSL in representative Tg*(krt18:Krt18-GFP)* control embryo injected with 0.2% phenol red (left) into the YSL and 50 pg *CARhoA* into marginal blastomeres at 3.3 hpf (right) imaged from 4.5-10.3 hpf. Lateral view. Frame rate10 min/frame. Scale bar: 100 µm.

Title: Supplementary Movie 5

Description: Keratin network maturation in EVL cells of embryos with enhanced YSL pulling force: Time-lapse of keratin network maturation upon increased pulling forces from the YSL in a representative Tg*(krt18:Krt18-GFP)* control embryo injected with 0.2% phenol red (left) and an embryo injected with 50 pg *CARhoA* into marginal blastomeres at 3.3 hpf (right) imaged from 4.5-7.5 hpf. Lateral view near the EVL-YSL boundary. Frame rate 10 min/frame. Scale bar: 25 µm.

Title: Supplementary Movie 6

Description: Keratin expression within the EVL of embryos with reduced keratin type II expression: Time-lapse of keratin expression in representative Tg*(krt18:Krt18-GFP)* embryos injected at the one-cell stage either with 2 ng control MO (control, left) or 1 ng *keratin 4* plus 1 ng *keratin 8* MO (right) imaged from 4-15.75 hpf. Lateral view. Frame rate 10.25 min/frame. Points of rupture in keratin morphant embryos are marked with asteriks. Scale bar: 100 µm.

Title: Supplementary Movie 7

Description: Keratin network maturation in EVL cells of embryos with reduced keratin type II expression: Time-lapse of keratin expression in representative Tg*(krt18:Krt18-GFP)* embryos injected at the one-cell stage either with 2 ng control MO (control, left) or 1 ng *keratin 4* plus 1 ng *keratin 8* MO (right) imaged from 4.5-8.4 hpf. Lateral view. Frame rate10 min/frame. Scale bar: 25 µm.

Title: Supplementary Movie 8

Description: Keratin expression within the EVL of embryo with reduced *keratin8* expression and rescue with modified mRNA: Time-lapse of keratin expression (top row) and actin (bottom row) in representative Tg*(actb2:Utrophin-mcherry, krt18:Krt18-GFP)* embryos injected at the one-cell stage either with 1 ng control MO (control, left) or 1 ng *keratin 8* MO (keratin8 MO, middle) or 1 ng *keratin 8* MO plus 100ng *keratin 8* mRNA (keratin8 rescue, right) imaged from 4-15.75 hpf. Lateral view. Frame rate 28.3 min/frame. Points of rupture in keratin morphant embryos are marked with asteriks. Scale bar: 100 µm.

Title: Supplementary Movie 9

Description: Changes in keratin expression upon EVL aspiration: Time lapse of EVL aspiration in a representative Tg*(keratin 18:Keratin 18-GFP)* embryo using a 60 µm pipette imaged by brightfield (left) and confocal (keratin, green, right) microscopy. Z-plane in the centre of the pipette. Frame rate 1 sec/frame. Scale bar: 25 µm.

Title: Supplementary Movie 10

Description: Changes in keratin expression during EVL wound closure after cell ablation: Time lapse of EVL in a representative Tg*(actb2:Utrophin-mcherry, keratin 18:Keratin 18-GFP)* embryo imaged before (pre) and after (post) UV laser-mediated cell ablation showing keratin (right, green) and actin (left, orange). Frame rate 20 sec/frame. Scale bar: 25 µm.

Title: Supplementary Movie 11

Description: Wound closure in control and keratin-deficient embryos: Time lapse of EVL response in representative Tg*(actb2:Utrophin-mcherry)* embryo imaged before (pre) and after (post) UV laser-mediated cell ablation injected at the one-cell stage either with 2 ng control MO (control, left) or 1 ng *keratin 4* plus 1 ng *keratin 8* MO (right). Frame rate 20 sec/frame. Scale bar: 25 µm.

Title: Supplementary Movie 12

Description: E-cadherin expression in control and keratin-deficient embryos: Time lapse of E-cadherin expression in Tg*(cdh1-YFP)xt17* embryos injected at the one-cell stage either with 2 ng control MO (control, left) or 1 ng *keratin 4* plus 1 ng *keratin 8* MO (right) starting at the beginning of epiboly (4 hpf) to failure of EVL integrity (14 hpf). Frame rate 10 min/frame. Scale bar: 25 µm.

Title: Supplementary Movie 13

Description: Occludin-b expression in control and keratin-deficient embryos: Time lapse of Occludin b expression in Tg*(oclnb-GFP)pd1126* embryos injected at the one-cell stage either with 2 ng control MO (control, left) or 1 ng *keratin 4* plus 1 ng *keratin 8* MO (right) starting at the beginning of epiboly (4 hpf) to the end of epiboly (10 hpf). Frame rate 10 min/frame. Scale bar: 25 µm.

Title: Supplementary Movie 14

Description: Jup-a expression in control and keratin-deficient embryos: Time lapse of Jup-a expression in Tg*(her4.1:jupa-EGFP)* embryos injected at at the one-cell stage either with 2 ng control MO (control, left) or 1 ng *keratin 4* plus 1 ng *keratin 8* MO (right) starting at the beginning of epiboly (4 hpf) to the end of epiboly (10 hpf). Frame rate 10 min/frame. Scale bar: 25 µm.

Title: Supplementary Movie 15

Description: Simulated EVL wound closure in model with and without keratin feedback onto mechanics: Time lapse of simulated wound closure following ablations in EVL model tissues without keratin mechanical feedback ('keratin deficient', top) and with keratin mechanical feedback ('control', bottom). Keratin concentration K_i in the cell is shown by colour (K_i: 0-150 red-yellow, K_i: 150-350 green). Tensile stress in the tissue t_i from the junctional tension of the cells around the wound is shown by the colour of the cell edges (rainbow t_i: 0.0 – 0.6). Orientation of the cell elongation is shown as a line through the cell center, and total displacement vectors since wound initiations are shown as arrows.

Title: Supplementary Movie 16

Description: Simulated EVL tissue undergoing epiboly: Time lapse of simulated EVL model tissue being stretched by radially outward forces increasing with time applied on the margin of the tissue, simulating the forces generated by the YSL on the EVL edge in the embryo. Keratin intensity K_i shown as the colour of the cells (left) increases with time in response to the stress experienced by the tissue under this force, starting from unpercolated levels ($K_i < K_{th}$, red-yellow) and progressing in each cell over the threshold keratin levels to percolated levels ($K_i > K_{th}$, green). Elevation of keratin levels in each cell over the threshold level is stochastic due to the heterogeneity in the mechanical effects on the disordered tissue. Tensile stress in the tissue t_i is shown by the colour of the cell edges (rainbow t_i: 0.0 - 0.6).

Title: Supplementary Movie 17

Description: Simulated recoil of EVL tissue upon wound formation: Time lapse of simulated wound recoil following ablations in EVL model tissues without keratin mechanical feedback ('keratin deficient', top) and with keratin mechanical feedback ('control', bottom). Keratin concentration K_i in the cell is shown by colour (K_i: 0-150 red-yellow, K_i: 150-350 green). Tensile stress in the tissue t_i from the junctional tension of the cells around the wound is shown by the colour of the cell edges (rainbow t_i: 0.0 – 0.6). Orientation of the cell elongation is shown as a line through the cell centre, and total displacement vectors since wound initiations are shown as arrows.

Title: Supplementary Movie 18

Description: Effect of normal and reduced YSL pulling force on control and keratin-deficient simulated EVL tissues: Time lapse of simulated EVL model tissue being stretched by radially outward forces increasing with time applied on the margin of the tissue, simulating the forces generated by the YSL on the EVL edge in the embryo. Keratin intensity K_i shown as the colour of the cells (left) with unpercolated levels ($K_i < K_{th}$, red-yellow) and percolated levels ($K_i > K_{th}$, green). Top left: Pulling force $F_{YSL} = 0.285$, no feedback $\beta = 0$. Top right: Pulling force $F_{YSL} = 0.285$, feedback $\beta = 0.005$. Bottom left: Pulling force $F_{YSL} = 0.57$, no feedback $\beta = 0$. Bottom right: Pulling force $F_{YSL} = 0.57$, feedback $\beta = 0.005$.
