# Supplementary material for: Keratins coordinate tissue spreading by balancing spreading forces with tissue material properties
Source: Nat Commun. 2026 May 16;17:6499. doi: 10.1038/s41467-026-72366-z (PMC13377027; doi:10.1038/s41467-026-72366-z)
Supplement: Supplementary file 22 — Transparent Peer Review file [file 41467_2026_72366_MOESM22_ESM.pdf]

nature portfolio

Peer Review File

Keratins coordinate tissue spreading by balancing spreading forces with tissue material properties.

Corresponding Author: Professor Carl-Philipp Heisenberg

This manuscript has been previously reviewed at another journal. This document only contains information relating to versions considered at Nature Communications.

This file contains all reviewer reports in order by version, followed by all author rebuttals in order by version.

Version 1:

Reviewer comments:

Reviewer #2

(Remarks to the Author)
This manuscript shows that keratin intermediate filaments function as a mechano-adaptive module during zebrafish epiboly: EVL keratins mature under stress to increase tissue viscosity and prevent rupture, while YSL keratins are essential for actomyosin contraction and flow that generate pulling forces. The study combines in vivo imaging and perturbations, micropipette-based analysis, and a vertex model with mechanochemical feedback, thereby linking cytoskeletal remodelling to tissue-scale material properties during epithelial spreading. The dual role of keratins in both the pulled (EVL) and pulling (YSL) tissues offers a compelling, systems-level perspective on epiboly mechanics and extends previous research on keratins in epithelial mechanics and extra-embryonic tissue expansion. This is an excellent manuscript, and only a few criticisms remain.

Major points

The added F0 CRISPR phenocopy strengthens specificity, but, in my opinion, a targeted rescue would definitely address MO concerns. Even a partial, cell-type-specific rescue (EVL or YSL) would be beneficial.

The authors argue that the sheet model is mathematically equivalent (for constant-pressure aspiration) to the spherical formalism. I would suggest including a brief supplementary figure that overlays fits from both formalisms on one dataset, which would help non-experts.

The YSL-specific krt4/8 MO data indicate a reduction in actomyosin flow; it would be beneficial to quantify (i) YSL actin/myosin PIV metrics and hydrodynamic length, and (ii) EVL edge velocity under YSL-only vs. EVL-only keratin perturbations, side-by-side. This approach would provide a clearer understanding of the dual role.

The actin dependency during aspiration suggests post-transcriptional or assembly regulation. I would like to read more about hat, and the authors could consider discussing potential molecular links (e.g., plectin/plakins, 14-3-3-dependent keratin remodelling, interaction with RhoA/ROCK),

Finally, the authors note that “keratins are absent in insects.” However, keratin-like proteins were reported in specific contexts.

Minor points

Report injected amounts/penetrance variability for DN-K18 and any toxicity controls.

In the main text/ figure legends, state numbers of embryos/biological replicates alongside plots for key assays (aspiration, wound, YSL-specific perturbations).

Check Video 15 - I was not able to watch it.

In summary, this is a strong study that meaningfully advances how we think about epithelial spreading by integrating cytoskeletal remodelling with tissue deformation and force generation. It will be a valuable resource for both zebrafish and epithelial mechanics communities. I am enthusiastic about the direction and look forward to the revision.

(Remarks on code availability)
N/A

Reviewer #4

(Remarks to the Author)
The manuscript reports on interesting work on the role of keratin networks, their mechano-sensitivity and their specific role in zebrafish development. The experimental results are quite impressive and insightful, but given my expertise, I will focus my comments on the modelling: In principle the methodology (vertex model) is convincing, but I am a bit doubtful about the match with experiments and whether much insight is gained from the model.

Some specific points:

The wound healing essay: Simulations (Fig 5H, I, J) & Experiments (Fig 4D-H).
I am missing quantitative evaluations of the simulations w.r.t. Cell alignment (Fig 4F') and tissue recoil (Fig 4G). The only checked prediction is the closure speed, which is probably driven by the acto-myosin cable.

Epiboly: Simulations (Fig 5K-P) & Experiments
- My first question is why in Fig 5K the most outwards cells have the least amount of keratin? This might just be a misunderstanding on my side, but I was still curious.
- It isn't clear to me why the keratin mutants/knock-downs are modelled via \beta=0 (no feedback of keratin on cells), and not via \alpha=0/small (less keratin production)
- The profile comparison Fig 5L to 5M is not convincing.
- Fig 5N: On a qualitative level, these predictions are quite obvious: More force -> feedback to keratin production --> higher keratin intensity. A quantitative comparison seems tricky, given that the initial states and precise applied forces in the experimental set-up aren't clear.
- Fig 5O: As the authors state, the predictions differs qualitatively from the experiments. They do suggest a potential reason (coupling of keratin to pulling force), but there appears to be no implementation and further simulations for this.

Another comment: For me Video 15 didn't work.

Overall, I think the modelling at this point doesn't lead to much insight, but in principle could, given more work.

(Remarks on code availability)

Reviewer #5

(Remarks to the Author)
The authors studied how spreading epithelia reconcile the need for large-scale deformability with the requirement to maintain structural integrity, focusing on keratin intermediate filaments as mechanosensitive regulators. They used various experimental techniques nicely combined with vertex simulations. They analyzed expansion of the enveloping cell layer (EVL) over the yolk in early zebrafish embryos in vivo and found that stresses accumulating during tissue spreading promote maturation of the keratin network within EVL cells.
The authors show that stress-driven keratin maturation increases EVL viscosity and thereby prevents tissue rupture. In addition, they demonstrate that keratins in the yolk cell are necessary for mechanosensitive actomyosin contraction and flow, which generate the pulling forces that drive EVL expansion. The work is sound and timely. The mechanical studies using a micropipette are, however, difficult to interpret especially in terms of continuum models such as the SLS or Maxwell solid. The authors might want to try larger pipette diameters to see whether their data are consistent. Alternatively parallel-plate compression could be helpful. But I also understand that this might be asking too much.

(Remarks on code availability)

Version 2:

Reviewer comments:

Reviewer #2

(Remarks to the Author)
The authors thoroughly and convincingly addressed my main points. They conducted an appropriate molecular rescue of the krt8 MO phenotype, demonstrating partial recovery of keratin network formation and EVL epiboly, which supports specificity.

They clarified the rheological modelling by explicitly mapping the sheet and spherical formalisms and explaining their assumptions.

They extended the experimental analysis of the dual EVL and YSL roles of keratin by adding new EVL mosaic clone data, revealing increased divisions and extrusions in keratin-deficient cells. This offers a biologically meaningful explanation for the variability in EVL expansion and explains why clean velocity comparisons are challenging.

All reporting and technical issues, including injected amounts, replicate numbers, and the faulty video, were properly addressed.

(Remarks on code availability)

Reviewer #4

(Remarks to the Author)
Thank you. My remarks have been adressed.

(Remarks on code availability)

Reviewer #5

(Remarks to the Author)
I have no further concerns; the authors have satisfactorily addressed my comments.

(Remarks on code availability)

Open Access This Peer Review File is licensed under a Creative Commons Attribution 4.0 International License, which permits use, sharing, adaptation, distribution and reproduction in any medium or format, as long as you give appropriate credit to the original author(s) and the source, provide a link to the Creative Commons license, and indicate if changes were made.
In cases where reviewers are anonymous, credit should be given to 'Anonymous Referee' and the source.
The images or other third party material in this Peer Review File are included in the article's Creative Commons license, unless indicated otherwise in a credit line to the material. If material is not included in the article's Creative Commons license and your intended use is not permitted by statutory regulation or exceeds the permitted use, you will need to obtain permission directly from the copyright holder.
To view a copy of this license, visit https://creativecommons.org/licenses/by/4.0/

Rebuttal letter - NCOMMS-25-36029A

Reviewer #2

This manuscript shows that keratin intermediate filaments function as a mechano-adaptive module during zebrafish epiboly: EVL keratins mature under stress to increase tissue viscosity and prevent rupture, while YSL keratins are essential for actomyosin contraction and flow that generate pulling forces. The study combines in vivo imaging and perturbations, micropipette-based analysis, and a vertex model with mechanochemical feedback, thereby linking cytoskeletal remodelling to tissue-scale material properties during epithelial spreading. The dual role of keratins in both the pulled (EVL) and pulling (YSL) tissues offers a compelling, systems-level perspective on epiboly mechanics and extends previous research on keratins in epithelial mechanics and extra-embryonic tissue expansion. This is an excellent manuscript, and only a few criticisms remain.

We thank the referee for his/her expert and constructive feedback.

Major points

The added F0 CRISPR phenocopy strengthens specificity, but, in my opinion, a targeted rescue would definitely address MO concerns. Even a partial, cell-type-specific rescue (EVL or YSL) would be beneficial.

Following the reviewer's advice, we performed rescue experiments by co-injecting *krt8* morpholinos with *krt8* mRNA that is modified to not be recognized by the *krt8* morpholino, and subsequently assessed EVL epiboly progression (Figure S3 F-H). These experiments revealed a partial rescue of both keratin filament-bundled network formation and EVL epiboly movements in the co-injected embryos compared with embryos injected with the morpholino alone. These results support the specificity of the morphant phenotype and are mentioned in the main text (line 211ff) and included as Figure S3 F-H in the revised manuscript.

The authors argue that the sheet model is mathematically equivalent (for constant-pressure aspiration) to the spherical formalism. I would suggest including a brief supplementary figure that overlays fits from both formalisms on one dataset, which would help non-experts.

Since the two models are mathematically equivalent, they will generate identical curves. To be more specific: the difference between both models is that we assume that the pipette aspiration probes the local rheology of the tissue at the scale of a few cells, while the spherical formalism assumes that one probes the global rheology at the level of the whole tissue spheroid. The standard model would require the assumption that the embryo, including the EVL, YSL and deep cells, is well modeled by a homogenous viscoelastic ball. We explain these assumptions in section 1 of the SI file and in the main text (line 377ff), and show the explicit mapping between parameters of both models in equations S6a-b in the SI file.

The YSL-specific krt4/8 MO data indicate a reduction in actomyosin flow; it would be beneficial to quantify (i) YSL actin/myosin PIV metrics and hydrodynamic length, and (ii) EVL edge

velocity under YSL-only vs. EVL-only keratin perturbations, side-by-side. This approach would provide a clearer understanding of the dual role.

We had previously quantified PIV metrics and the hydrodynamic length scale of actin flows within the YSL, as well as EVL edge velocities, in both wild-type and YSL-specific *krt4/8* morphant embryos (Figure 7D and Figure S7D–F). In addition, we have now analyzed the behavior of *krt4/8* morphant cell clones within the EVL. This analysis revealed that morphant EVL cells tend to undergo more frequent cell divisions and cell extrusions compared with neighboring wild-type EVL cells. As a consequence, the expansion rate of morphant cells was highly variable, likely reflecting the increased incidence of divisions and extrusions, which makes a robust quantitative comparison between morphant and wild-type cells challenging. We have included these new data as Referee Figure 1, as we remain cautious about their conclusiveness, but would be happy to move them to the Supplementary Information if the referee considers this more appropriate.

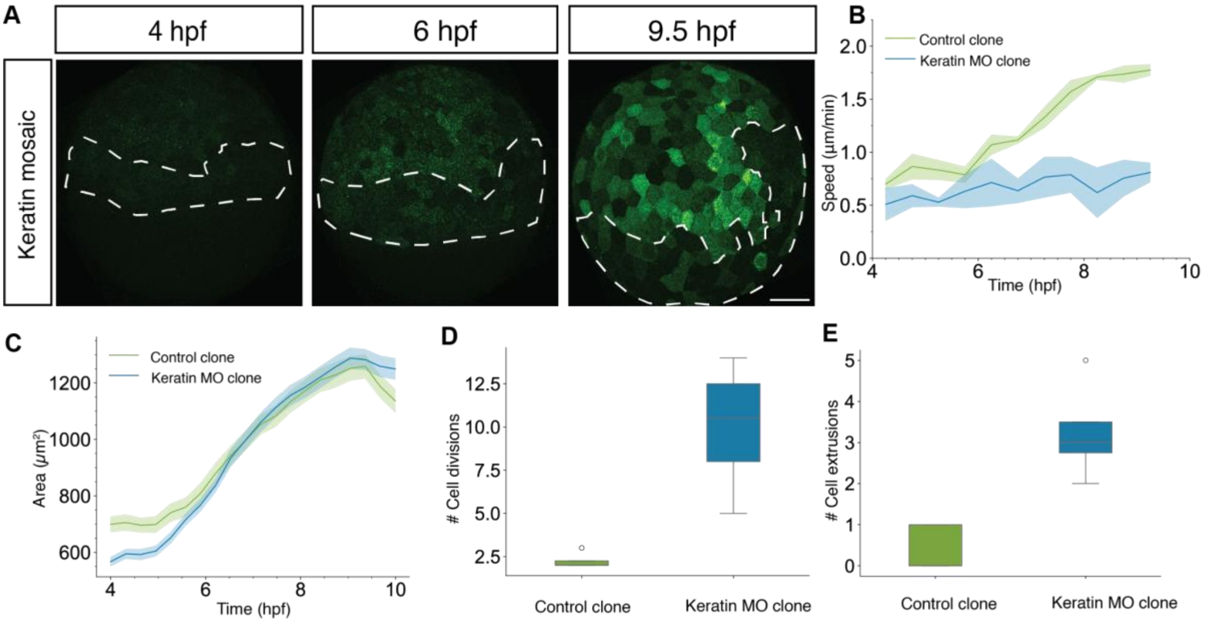

Referee Figure 1

(A) Maximum intensity projection images of keratin expression in Tg(krt18:Krt18-GFP) (keratin) embryos, injected at the 128-cell stage into a single blastomere with1 ng krt4 plus 1 ng krt8 MO, at 4 (left), 6 (middle) and 9.5hpf (right), showing the progression of keratin expression in injected/morphant versus control EVL cells. The outline of the morphant EVL cell clone is marked by the dashed line. Control EVL cell clones of similar size were chosen in the proximity of the morphant clones.

(B) Plot of EVL clonal expansion speed as a function of time (hpf) during epiboly, starting at sphere stage (4 hpf) until late epiboly stages (9.5 hpf) for the conditions described in (A).

(C) Plot of average EVL clonal area as a function of time (hpf) for the conditions described in (A). Error bars as SD of the mean of replicates.

(D) Boxplot of number cell divisions in EVL clones for the conditions described in (A).

(E) Boxplot of number cell extrusions in EVL clones for the conditions described in (A).

Error bars (A-C) as SD of the mean of replicates. (N=3; n=4). Scale bar (A), 100µm.

The actin dependency during aspiration suggests post-transcriptional or assembly regulation. I would like to read more about that, and the authors could consider discussing potential molecular links (e.g., plectin/plakins, 14-3-3-dependent keratin remodelling, interaction with RhoA/ROCK),

We have added a more detailed discussion on this topic in the revised manuscript (line 512ff).

Finally, the authors note that “keratins are absent in insects.” However, keratin-like proteins were reported in specific contexts.

We have corrected this in the revised manuscript (line 528ff).

Minor points

Report injected amounts/penetrance variability for DN-K18 and any toxicity controls.

We have added this information in the revised manuscript.

In the main text/ figure legends, state numbers of embryos/biological replicates alongside plots for key assays (aspiration, wound, YSL-specific perturbations).

We have added this information in the revised manuscript.

Check Video 15 - I was not able to watch it.

We have added a new version of this movie (libx264 format) in the revised manuscript.

In summary, this is a strong study that meaningfully advances how we think about epithelial spreading by integrating cytoskeletal remodelling with tissue deformation and force generation. It will be a valuable resource for both zebrafish and epithelial mechanics communities. I am enthusiastic about the direction and look forward to the revision.

Reviewer #4

The manuscript reports on interesting work on the role of keratin networks, their mechano-sensitivity and their specific role in zebrafish development. The experimental results are quite impressive and insightful, but given my expertise, I will focus my comments on the modelling: In principle the methodology (vertex model) is convincing, but I am a bit doubtful about the match with experiments and whether much insight is gained from the model.

We thank the referee for his/her expert and constructive feedback. We have carried out further work to improve the match with the experimental data, which we hope will convince the referee that there is some important and meaningful insight coming from the model. We provide a point-by-point response below.

Some specific points:

The wound healing essay: Simulations (Fig 5H, I, J) & Experiments (Fig 4D-H).
I am missing quantitative evaluations of the simulations w.r.t. Cell alignment (Fig 4F') and tissue recoil (Fig 4G). The only checked prediction is the closure speed, which is probably driven by the acto-myosin cable.

We thank the referee for pushing us to carry out further comparisons between experimental data and simulations of EVL cell displacements surrounding the ablation site, both during the initial tissue recoil and during subsequent wound closure.

For the new simulations of tissue recoil, we modeled a wound *without* an added contractile cable and measured the early-time dynamics (10s after ablation). For simulating wound closure, the cable was introduced immediately after ablation. As the wounds are anisotropic due to the linear cut (as in the experiment), we characterized the tissue as a function of the distance of the edge, measured in cell rows where row 1 is composed of the cells directly adjacent to the wound, 2 are their neighbors and so on.

The cell alignment analysis turned out to be very sensitive to the exact wound geometry and shape of the surrounding cells and did not produce reliable and comparative data between experiments and simulations. We therefore decided to instead compare cell flow patterns towards the ablation site during the wound closure phase.

We have now split the model results into two new figures (Figures 5 and 6), showing the model development and pipette results, and the wound and epiboly simulations, respectively.

New Figure 6E-F shows the recoil velocity in both control and Keratin deficient embryos. We found, as in the experiment, that the recoil velocity in the control embryos is higher than in the Keratin deficient embryos, and that it spreads further into the tissue. The reason for this is the higher tension in the tissue, which increases the contractile forces experienced by the wound edge and tissue interior (see new supplementary movie S16). We would also like to point out that in the revision we discovered an incorrect unit conversion for the experimental data. The revised Figure 4E-F' and Figure 6F - I shows that experimental and simulation velocities are indeed comparable.

In new Figures 6G and 6H, we show the displacement of cell centroids in the tissue during the different stages of wound closure for control and Keratin deficient simulations, respectively. The differences between these conditions are shown in Figure 6I, demonstrating that motion during wound closure is larger close to the wound for keratin deficient embryos, but spreads more widely into the tissue for the control embryos, similar to our experimental observations (reproduced as Referee Figure 2 below).

In addition, we have replaced the panels previously shown in Figure 5H and 5I with snapshots taken closer to the end of the simulated wound closure, when stress propagation into the tissue is more apparent, and have highlighted the displacements of each individual cell centroid (Figure 6A-D).

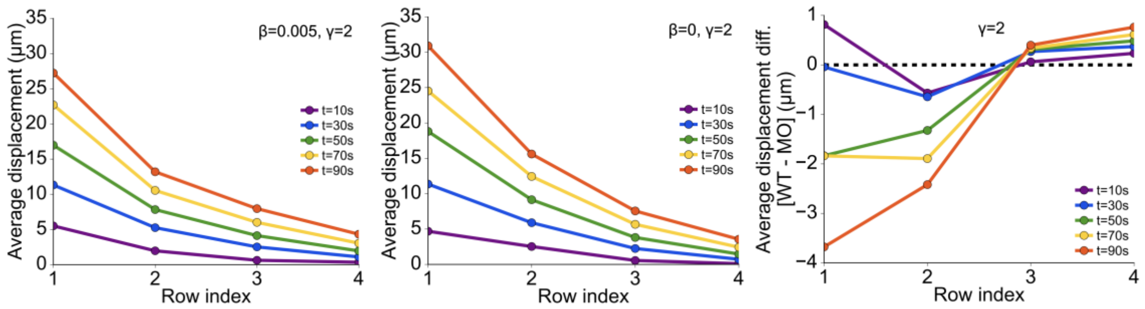

Referee Figure 2
Displacements of cell centroids averaged within cell rows, in closure simulations (γ=2), in both the (left) wild-type (β=0.005) and (center) keratin-deficient (β=0) scenarios. (right) Difference from the displacements in the keratin-deficient to the wild-type scenarios (Figures 6G -I).

Collectively, these comparisons show that, in both experiments and simulations, cell displacements during recoil and closure are detectable at greater distances from the ablation site in wild-type embryos than in keratin-defective embryos, further supporting our conclusion that efficient force propagation within the EVL tissue depends on keratin expression and network formation.

Epiboly: Simulations (Fig 5K-P) & Experiments
- My first question is why in Fig 5K the most outwards cells have the least amount of keratin? This might just be a misunderstanding on my side, but I was still curious.

The lower keratin values in the outer ring of cells are due to our boundary conditions in the numerical model: The keratin-stress feedback implementation requires that we calculate the stress tensor of individual cells in the vertex model. The resultant expression (line 304ff of the revised manuscript, SI eq. S19) contains contributions from both area and perimeter terms, typically of opposite sign. Cells on the boundary have additional tension from the outward pulling force and bounding cable, leading to modified cell shapes and stresses. This leads to feedback nonlinearities and ultimately numerical instability when using the full expression. We therefore used only the area contribution of the stress for those cells, leading to lower stresses and therefore lower keratin values. This change affects only the behavior of the outer boundary cells and does not have a qualitative effect on the heterogeneity of stress and keratin distributions within the tissue, nor on the tissue-edge velocity as a function of the pulling force. For more details, please also see supplementary information section 2.

- It isn't clear to me why the keratin mutants/knock-downs are modelled via \beta=0 (no feedback of keratin on cells), and not via \alpha=0/small (less keratin production)

Indeed, using α=0 (no keratin production) instead of β=0 (no keratin feedback) is a conceptually better representation of the situation in the keratin mutants. The mechanical effects of both choices, however, are mathematically identical: when no keratin is produced for α=0, K=0 and therefore the keratin contribution term to the mechanics, $\beta\Delta K=\beta max(0, K\text{-}K_{th})$ =0 just as much as when β=0. To avoid any misunderstandings, we have mentioned this in the revised version of the manuscript (line 339ff). The reason for keeping with β=0 is that this allows us to simulate keratin expression in the absence of mechanical feedback, something currently not possible through experiments.

To illustrate this, we compared in Referee Figure 3 below different conditions, with the total simulated tissue area on the left, and the mean keratin concentration on the right. Specifically, we compared the mutant scenario in the paper (α=120000, β=0) to α=0, β=0.005 (corresponding to no keratin production but feedback), α=0, β=0 (corresponding to no keratin production and no feedback), and $\alpha = 120000, \beta = 0.005$ (corresponding to both keratin production and feedback, i.e. the wild type condition). The total area evolution of the three mutant scenarios is identical. The mean keratin expression in the $\alpha = 0$ simulated embryos remains 0 while in the $\beta = 0$ simulated mutants, it is in fact higher, showing the nontrivial couplings introduced by the mechanochemical feedback.

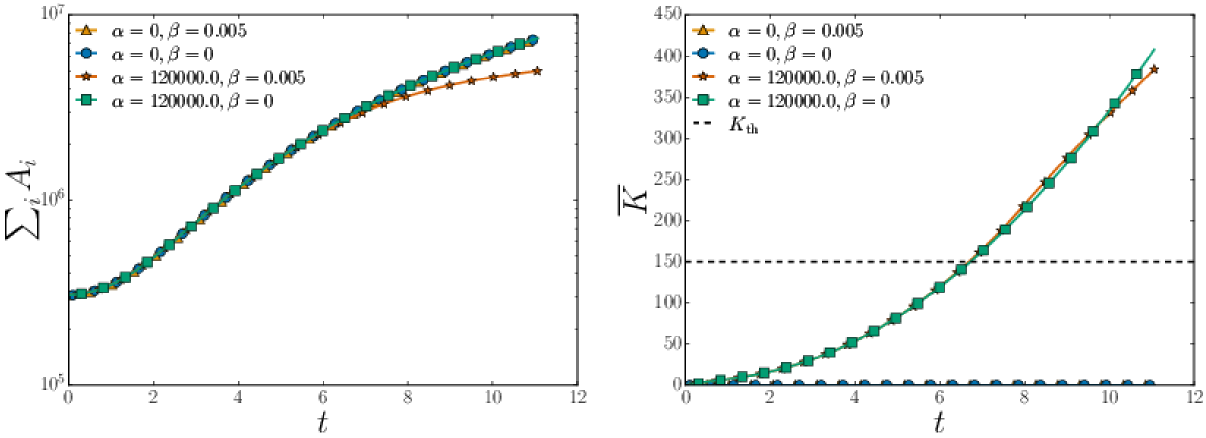

Referee Figure 3
Total simulated epiboly tissue area (left) and mean keratin expression (right) as a function of time for the three keratin deficient (mutant) cases and the wild-type.

- The profile comparison Fig 5L to 5M is not convincing.

The relation between keratin heterogeneity and mechanics is subtle: The disordered packing geometry leads to heterogeneous stresses, but the amount depends sensitively on the shape factor $s_0 = \frac{P_0}{\sqrt{A_0}}$ (see Referee Figure 4). Systems with $s_0 \geq 3.81$ are not (linearly) rigid and have stress-free floppy modes. Pulling still leads to stress in the system, but without notable heterogeneities, and the keratin response is nearly homogenous. The opposite is true for $s_0 < 3.81$: states of self-stress with heterogeneous patterns become prominent, which is also visible in the keratin response, and is in fact amplified by the keratin-stress feedback loop.

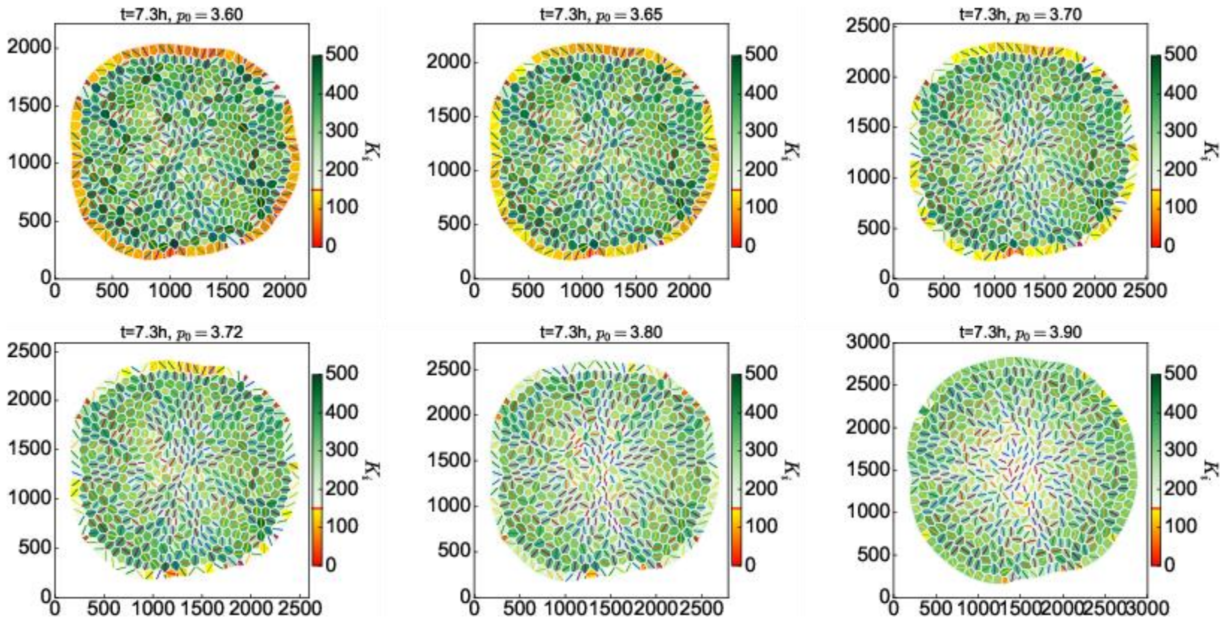

Referee Figure 4
Epiboly simulations at the same time point and using the same initial configuration for different shape factors s_0. The heterogeneity decreases from high to low s_0, together with the disappearance of states of self stress.

We chose $s_0 = 3.72$ in the original version of the manuscript, based on it being a stress-free state for an ordered packing, but there is considerable latitude (though little experimental information) for choosing a lower value.

Separately, another source of mechanical heterogeneity is the subcellular scale. Our results suggest that the actomyosin network acts as a scaffold or environment for keratin filament assembly, and that this is the main source of the stress sensitivity. Both networks are heterogeneous, and not identical from cell to cell. In our model, this would be represented by heterogeneity in either α or β coupling coefficients.

To illustrate this, we chose to introduce a moderate 20% heterogeneity in α in the simulated cells (Referee Figure 5, left), and compared it to the original simulation with homogenous α (Referee Figure 5, right). The heterogeneity does not only lead to considerably wider keratin distributions as expected, it is also tightly coupled to the mechanical feedback and cell sizes become visibly more heterogeneous, like in the experiments. This suggests that intracellular heterogeneity is a more realistic representation of the biological system.

We now discuss the different origins of mechanical heterogeneity in the main text (line 390ff). We have also included a new section 'Enhancing keratin heterogeneities' as section 7 of the supplementary information of the revised manuscript, which includes versions of Referee Figures 4 and 5 as Figures S6 and S7.

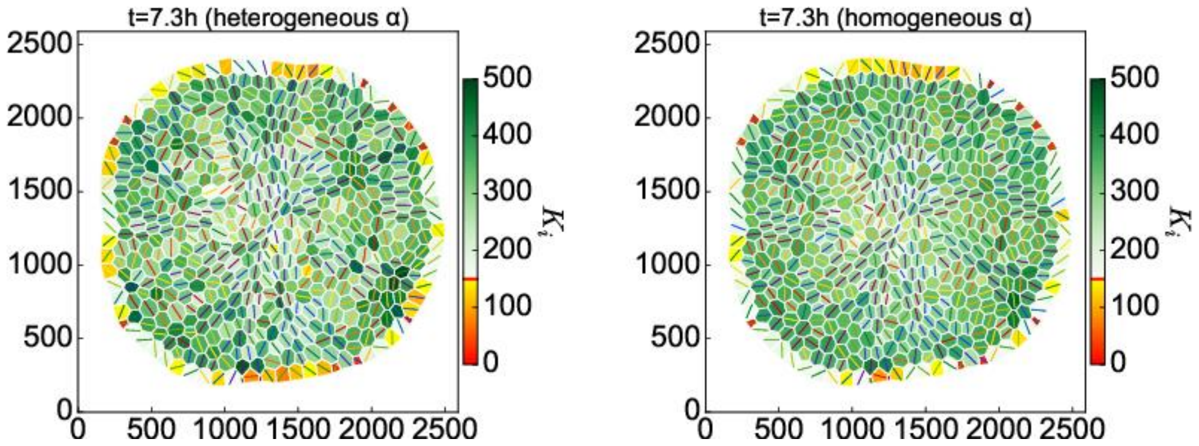

Referee Figure 5

Epiboly simulation snapshots in the wild-type scenario ($\beta = 0.005$, $\alpha = 1.2 \times 10^5$) showing keratin expression levels Ki in each cell. Left: 20% heterogeneous α, right homogeneous α .

- Fig 5N: On a qualitative level, these predictions are quite obvious: More force -> feedback to keratin production --> higher keratin intensity. A quantitative comparison seems tricky, given that the initial states and precise applied forces in the experimental set-up aren't clear.

The predictions of the model are not necessarily obvious:

There is a separation of length and of time scales between the action of the feedback at the scale of a cell and on the time scale of a minute, and the effect on epiboly at the scale of several hundred cells and on the time scale of hours.

The quantitative comparison is indeed tricky, which is why we employ the pipette experiment as a proxy to measure the feedback dynamics at the local cell scale and time scales of minutes. We went through considerable crosschecks, including the mean field model and the wound simulations, to assure that the quantitative mapping is as appropriate as we can make it. The fact that we obtain a semi-quantitative match to the epiboly experiment, not simply a qualitative one, is proof that we can successfully bridge this gap in scales. The forces exerted by the YSL on the embryo that we use can in fact be considered predictions.

The emergent heterogeneity of keratin expression is a purely emergent consequence of the heterogeneous stresses inside the disordered tissue, a result that could not have been inferred without constructing the full model.

The mechanical effect of the keratin on epiboly itself, in the end, is a subtle one. It appears to allow for an adaptive response by acting as a rheostat, regulating the tissue stiffness and viscosity in such a way that different stress conditions can be buffered. This ultimately becomes clear only in the nullcline analysis of the mean field mode (section 4 of the SI file): we find that the tissue strain rate is nearly independent of the excess keratin value, which itself is set by the applied stress to the tissue (Figure S6A). We have clarified this point in the manuscript.

Of course, more detailed molecular and cellular insight into the *in vivo* condition of EVL tissue expansion during epiboly would be advantageous. It would then also allow us to construct a model that includes more detail on the keratin network and its assembly and the interactions with the cytoskeleton – something, however, which is beyond the scope of this study.

- Fig 5O: As the authors state, the predictions differs qualitatively from the experiments. They do suggest a potential reason (coupling of keratin to pulling force), but there appears to be no implementation and further simulations for this.

This is an important point, which we had addressed through simulations that vary the pulling force for the simulated mutants in the original Figure 5O-P. We have revised the presentation for clarity, with all simulations with feedback (Figure 6N) and all simulations without feedback (Figure 6O) grouped together in the revised manuscript. We have more carefully explained the rheostat point in the main text (line 412ff), and complemented it with new supplementary video 17 that shows epiboly at different pulling forces with and without feedback. The main conclusion is that the experimental observations are consistent with a reduced pulling force from the YSL in the Keratin deficient embryos, consistent with subsequent experiments included in Figure 7.

Another comment: For me Video 15 didn't work.

We have added a new version of this movie (libx264 format) in the revised manuscript.

Overall, I think the modelling at this point doesn't lead to much insight, but in principle could, given more work.

We hope that with our revisions we can convince the referee that there is some important and meaningful insight coming from the model.

Reviewer #5

The authors studied how spreading epithelia reconcile the need for large-scale deformability with the requirement to maintain structural integrity, focusing on keratin intermediate filaments as mechanosensitive regulators. They used various experimental techniques nicely combined with vertex simulations. They analyzed expansion of the enveloping cell layer (EVL) over the yolk in early zebrafish embryos in vivo and found that stresses accumulating during tissue spreading promote maturation of the keratin network within EVL cells.
The authors show that stress-driven keratin maturation increases EVL viscosity and thereby prevents tissue rupture. In addition, they demonstrate that keratins in the yolk cell are necessary for mechanosensitive actomyosin contraction and flow, which generate the pulling forces that drive EVL expansion. The work is sound and timely.

We thank the referee for his/her expert and constructive feedback.

The mechanical studies using a micropipette are, however, difficult to interpret especially in terms of continuum models such as the SLS or Maxwell solid. The authors might want to try larger pipette diameters to see whether their data are consistent. Alternatively parallel-plate compression could be helpful. But I also understand that this might be asking too much.

We have repeated the aspiration experiments using a larger pipette (80 and 120µm inner diameter) and obtained very similar data as the ones obtained with the smaller pipette (60 µm), supporting the validity of our measurements. We have mentioned these findings in the main text (line 229ff) and included them in the revised manuscript as (Figure Supplement 4G).
